# A review of the genus *Muusoctopus* (Cephalopoda: Octopoda) from Arctic waters

**DOI:** 10.1186/s40851-023-00220-x

**Published:** 2023-11-16

**Authors:** Alexey V. Golikov, Gudmundur Gudmundsson, Martin E. Blicher, Lis L. Jørgensen, Ekaterina I. Korneeva, Steinunn H. Olafsdottir, Elena I. Shagimardanova, Leyla H. Shigapova, Denis V. Zakharov, Olga L. Zimina, Rushan M. Sabirov

**Affiliations:** 1 GEOMAR Helmholtz Centre for Ocean Research Kiel, Dusternbrooker Weg 20, 24105 Kiel, Germany; 2 Collections and Systematics Department, Icelandic Institute of Natural History, Urriðaholtsstræti 6–8, 210 Gardabaer, Iceland; 3 NIRAS A/S, Ceres Allé 3, 8000 Aarhus, Denmark; 4 Greenland Institute of Natural Resources, Greenland Climate Research Centre, Kivioq 2, 3900 Nuuk, Greenland; 5 Institute of Marine Research, Tromsø Department, P.O. Box 6606 Langnes, 9294 Tromsø, Norway; 6 Department of Zoology, Kazan Federal University, Kremlyovskaya Str. 18, 420008 Kazan, Russia; 7 Marine and Freshwater Research Institute, Demersal Division, Fornubúðir 5, 220 Hafnarfjordur, Iceland; 8 Kazan Federal University, Extreme Biology Laboratory, Volkova Str. 18, 420021 Kazan, Russia; 9 Zoological Institute of Russian Academy of Sciences, Laboratory of Marine Research, Universitetskaya Nab. 1, 199034 Sankt-Petersburg, Russia; 10 Murmansk Marine Biological Institute, Laboratory of Zoobenthos, Vladimirskaya Str. 17, 183010 Murmansk, Russia

**Keywords:** Biogeography, *COI*, Deep-sea, Ecology, Incirrata, Morphology, North Atlantic, Reproduction, Slope, Taxonomy

## Abstract

We report two Arctic species of incirrate octopods new to science. One is formally described here as *Muusoctopus aegir* Golikov, Gudmundsson & Sabirov **sp. nov.** while the other, *Muusoctopus* sp. 1, is not formally described due to a limited number of samples (all are immature individuals). These two species differ from each other, and from other *Muusoctopus*, especially in: 1) absence of stylets (in *M. aegir* **sp. nov.**); 2) proportions of mantle and head; 3) funnel organ morphology (W-shaped with medial and marginal limbs of equal length in *M. aegir* **sp. nov.**, or medial are slightly longer; V V-shaped with medial limbs slightly longer and broader than marginal in *Muusoctopus* sp. 1); 4) sucker and gill lamellae counts; 5) relative arm length and sucker diameter; and 6) male reproductive system relative size and morphology. Species of *Muusoctopus* now comprise four of 12 known Arctic cephalopods. Additionally, this study provides: a) new data on the morphology and reproductive biology of *M. johnsonianus* and *M. sibiricus*, and a diagnosis of *M. sibiricus*; b) the equations to estimate mantle length and body mass from beak measurements of *M. aegir* **sp. nov.** and *M. johnsonianus*; c) a cytochrome *c* oxidase subunit I gene barcode for *M. sibiricus*; d) new data on the ecology and distribution of all studied species; and e) a data table for the identification of northern North Atlantic and Arctic species of *Muusoctopus*.

**Supplementary Information:**

The online version contains supplementary material available at 10.1186/s40851-023-00220-x.

## Background

The majority of octopods of the suborder Incirrata Grimpe, 1916 [1] are carnivorous benthic animals with fast growth rates and short life cycles. This group is an important component of the seafloor ecosystems from polar to tropical areas, from littoral to bathyal depths [2, 3]. Most incirrate octopods belong to the superfamily Octopodoidea Orbigny, 1840 [4], in which five of six families are benthic [5, 6]. These include the Bathypolypodidae Robson, 1929 [7], Eledonidae Rochebrune, 1884 [8], Enteroctopodidae Strugnell, Norman, Vecchione, Guzik & Allcock, 2014 [9], Megaleledonidae Taki, 1961 [10] and Octopodidae Orbigny, 1840 [4–6]. There are more than 300 species of Octopodoidea, many of which have not been formally described [5, 6]. The commercial importance and catch rates of octopods are increasing worldwide [11], along with an upsurge in other anthropogenic influences on the oceans [12]. This can potentially lead to species extinction outpacing biodiversity assessment and description [13], especially given the global decrease in taxonomic studies [14]. Moreover, these five families of benthic octopods include many deep-sea representatives [3, 5, 15], which are particularly understudied and prone to environmental and anthropogenic stresses, such as those described in [16, 17].

The taxonomy, life histories, and distributions of deep-sea North Atlantic cephalopods are not well known [18]. Deep-sea octopods of the genera *Bathypolypus* Grimpe, 1921 [19] and *Muusoctopus* Gleadall, 2004 [20] (formerly *Benthoctopus* Grimpe, 1921 [19]) and *Graneledone verrucosa* (Verrill, 1881) [21] are the most common incirrate octopods in North Atlantic lower shelf and slope areas [22–25]. All lack an ink sac, and *Bathypolypus* and *Muusoctopus* have biserial suckers, whereas *G. verrucosa* has uniserial suckers [23, 26]. Following Muus [23], three of the five species of *Bathypolypus* from North Atlantic waters occur in Arctic waters: *B. arcticus* (Prosch, 1847) [27], *B. bairdii* (Verrill, 1873) [28] and *B. pugniger* Muus, 2002 [23]. Voss and Pearcy [29] suggested that the holotype of *Benthoctopus piscatorum* (Verrill, 1879) [30], which is the type species of the genus (Grimpe, 1921) [19], belongs to the genus *Bathypolypus*. It was later confirmed to be a junior synonym of *B. bairdii* by Muus [23] and Allcock et al*.* [26]. The transition of *Benthoctopus* to a junior synonym of *Bathypolypus* resulted in all deep-sea non-*Bathypolypus* biserial inkless octopods lacking a valid genus name [20]. Later, most of them were listed in the genus *Muusoctopus* Gleadall, 2004 [31, 32], which currently includes 27 species [33]. Octopods caught in the North Atlantic and Arctic which were previously assigned to the species *Be. piscatorum* became impossible to assign to any known species [22, 34–54]. Two species of *Muusoctopus*, one new and one resurrected, were described from the northeast Atlantic by Allcock et al*.* [26]: *Muusoctopus normani* (Massy, 1907) [37] and *M. johnsonianus* (Allcock, Strugnell, Ruggiero & Collins, 2006) [26]. These species are known from slope areas of the North Atlantic from 38°N (*M. johnsonianus* was recently found at 15°N by Luna et al*.* [55]) to 60°N in the eastern Atlantic, and apparently at about the same latitudes in the western Atlantic and along the Mid-Atlantic Ridge [22, 24–26, 32, 56] [M. Vecchione, pers. comm.; C. Nozères, pers. comm.]. Records in the western Atlantic are largely unpublished, excepting Pratt et al*.* [25]. The northernmost record of *Muusoctopus* spp. in the northwest Atlantic is the entrance of Ungava Bay at about 60°N [C. Nozères, pers. comm.: photos checked by A.V.G.]. Depth records for *M. normani* and *M. johnsonianus* are 500–1843 m and 797–2540 m, respectively [22, 25, 26, 32, 55–57]. Data on the associated bottom temperatures are absent. Also, *M. normani* is sometimes synonymized with *M. januarii* (Hoyle, 1885) [58] [32]. However, only a single individual of *M. januarii* was analysed in Gleadall [32], whose characteristics do not fully fit *M. normani* [25, 32, 58, 59], and no genetic analysis has been performed on *M. januarii*. Moreover, the known geographical range of *M. januarii* is much further south, i.e., from the Gulf of Mexico to Brazil, and the depth ranges of these species do not coincide [25, 32, 58, 59]. Thus, after comparing *M. januarii* from Toll [59] and *M. normani* from Allcock et al*.* [26] and Gleadall [32], the present study treats *M. normani* as a separate species.

The northern distributional limits of both *M. normani* and *M. johnsonianus* are the Canada–Greenland and Greenland–Iceland–Faroe Ridges, which coincide with natural borders between biogeographic provinces of the boreal Atlantic and Arctic deep-seas [60, 61]. Records of these species to the north of that border do not exist. An inventory of accepted *Muusoctopus* from Arctic waters includes *M. sibiricus* (Løyning, 1930) [62], which inhabits the Laptev, East Siberian, Chukchi and Beaufort Seas [49–52, 63], and *M. leioderma* (Berry, 1911) [64], found in areas of the Chukchi Sea, adjacent to the Bering Strait [49, 50, 65–67]. Records of other *Muusoctopus* species from the Pacific Arctic are considered misidentifications: 1) *M. profundorum* (Robson, 1932) [43] from the Chukchi Sea [66, 68] is considered to be *M. sibiricus* [50, 63] [I. G. Gleadall, pers. comm.]; 2) *M. hokkaidensis* (Berry, 1921) [69] from the Chukchi Sea [68, 70, 71] is considered to be *Muusoctopus* sp. or *M. sibiricus* [50, 63], with *Muusoctopus* sp. later considered to be *M. sibiricus* as well [I. G. Gleadall, pers. comm.]; 3) ‘Octopus’ from the Chukchi Sea [72] is considered to be *M. sibiricus* in the present study, as it occurs far from the Bering Strait. *Muusoctopus leioderma* records from the Chukchi Sea by Feder et al*.* [73] are also distant from the Bering Strait. They most likely belong to *M. sibiricus* and are treated as such in this study. Depth records of *M. sibiricus*, 30–220 m, are associated with bottom temperatures of –1.4–1.6 °C [51, 62, 63, 70–72, 74]. Depth records for *M. leioderma*, 38–1760 m (40–80 m in the Arctic) are associated with bottom temperatures of –1.0–4.9 °C [50, 65–67].

Records of what had previously been considered *Be. piscatorum* within the Arctic and Subarctic areas are known along the northern slope of the Greenland–Iceland–Faroe Ridge, in the Faroe–Shetland Channel, along the Norwegian slope, at the deep-sea sides of the Svalbard slope, and at the deep-sea side of the Severnaya Zemlya slope [34–36, 40–47, 49–52]. The depth records are 86–2000 m and are associated with a bottom temperature of –0.9 °C [34, 36, 41, 42, 44, 51, 57]. This species was found to be an undescribed species of *Muusoctopus* [75]. We herein describe this species on the basis of a large collection of individuals (*n* = 37) and present information on its biology, ecology, and distribution. We also report a second Arctic species to the genus *Muusoctopus,* but refrain from describing it further because of limited material (*n* = 4 immature individuals). Additionally, this study provides: a) new data on the morphology and reproductive biology of *M. johnsonianus* and *M. sibiricus*, and a diagnosis of *M. sibiricus*; b) equations for estimation of mantle length (ML) and body mass from beak measurements of the new species of *Muusoctopus* and *M. johnsonianus*; c) a cytochrome *c* oxidase subunit I gene (*COI*), i.e. DNA barcode for *M. sibiricus*; d) new data on the ecology and distribution of all studied species; and e) a table for identifying northern North Atlantic and Arctic *Muusoctopus* species.

## Materials and methods

### Sample collection, fixation and storage

**Fig. 1:**
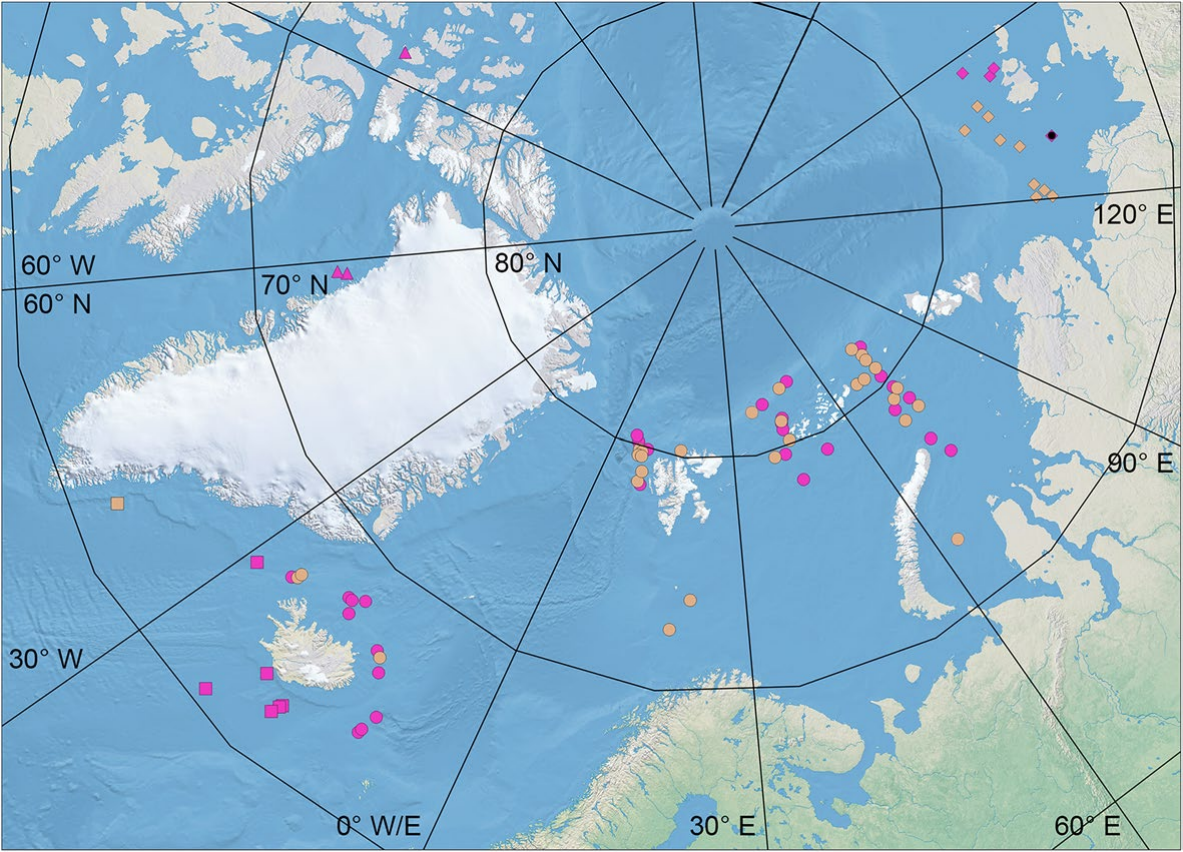
Sampling locations of *Muusoctopus* Gleadall, 2004 [20] in the Arctic and North Atlantic. Circles = *M. aegir* Golikov, Gudmundsson & Sabirov, **sp. nov.**; squares = *M. johnsonianus* (Allcock, Strugnell, Ruggiero & Collins, 2006) [26]; triangles = *Muusoctopus* sp. 1; rhombs = *M. sibiricus* (Løyning, 1930) [62]. Bright magenta color indicates the stations where individuals were analyzed. Pale brown color indicates stations where no individuals were analyzed. The black dot indicates the station where the sample was taken for cytochrome *c* oxidase subunit I gene

Octopuses were collected off Iceland in 1991–2017 by the Icelandic Institute of Natural History, Reykjavik (IINH) and Marine and Freshwater Research Institute, Reykjavik (MFRI); off East and West Greenland in 2016 by the Greenland Institute of Natural Resources, Nuuk (GINR); in the Barents Sea in 2007–2018 by the Institute of Marine Research, Bergen (IMR) and Polar Branch of All-Russian Fisheries Research Institute of Fisheries and Oceanography, Murmansk (PINRO); in the Kara Sea in 2007–2013 by PINRO; and in the Laptev and East Siberian Seas in 2014 by the Murmansk Marine Biological Institute, Murmansk (MMBI) (Fig. 1). Additionally, hitherto unpublished data for one individual referred to *Muusoctopus* sp. from the United States National Museum of Natural History, Smithsonian Institution, Washington (USNM) were provided by A. L. Allcock [A. L. Allcock, unpublished data]. Additional collection acronyms include: Eduard Eversman Zoological Museum of Kazan Federal University, Kazan (ZM KFU); Laboratory of Hydrobiology, Department of Zoology, Kazan Federal University, Kazan (LH KFU); and Zoological Institute of Russian Academy of Sciences, Sankt-Petersburg (ZIAS). Exact locations and associated environmental parameters of stations and details of individuals are provided in ‘Material examined’ sections for each species.

Individuals were collected either as bycatch during annual bottom trawl surveys of ground fish stocks (MFRI and GINR), or during scientific research cruises (IINH, IMR, PINRO, and MMBI). Octopods were fixed in 10% formalin onboard. Tissue sample was taken prior to that for DNA analysis from one individual of *M. sibiricus* (LS-L-3). The tissue sample was kept in 96% ethanol in the freezer (–20 °C).

### Morphological and statistical analyses

Counts, measurements, and indices were made following the general guidelines for cephalopods [76] and previous studies on *Muusoctopus* morphology: in particular, total arm sucker count was employed, not basal half [26, 31, 32, 77]. The beak measurements follow Clarke [78]. The right side was chosen for arms, eyes, and gills, and the left side was used as a substitute in case the right one was unavailable. Values are given as minimum to maximum (mean ± SE), unless otherwise stated. All indices are related to ML unless otherwise stated, and are always detailed when used for the first time. Maturity stages were assigned on a scale modified from Sauer & Lipinski [79] and Nigmatullin et al*.* [80], where:

0 = juvenile (reproductive system so small it can not be seen without stereomicroscope and is not fully developed; translucent in color);

I = early immature (reproductive system still very small, but fully formed, and visible without stereomicroscope; still translucent in color);

II = late immature (reproductive system is larger than on previous stage and not translucent; in females, ovary is full of similar-sized oocytes);

III = early maturing (reproductive system is large, it occupies 1/3 to half of the volume of the mantle cavity; in males, spermatophoric complex has no sperm inside; in females, oocytes of two different size groups present in the ovary);

IV = late maturing (reproductive system large, it occupies more than a half of the volume of the mantle cavity; in males, spermatophoric complex has sperm inside sperm duct and proximal spermatophoric glands, which can be recognized by their whitening, and tentative spermatophores can be present; in females, large vitellogenic oocytes present in the ovary);

V_1_ = pre-mature (reproductive system large, it may be even larger than at the previous stage; up to five normal spermatophores present in males; first ripe oocytes present in females);

V_2_ = mature (reproductive system large, it is proportionally the largest of all the stages; more than five spermatophores present in males; ripe oocytes and possibly post-ovulatory follicles present in females);

V_3_ = pre-spent (gonad is degraded and of reduced size, the size is roughly as in early maturing individuals; number of spermatophores is equal to that in mature males; residual ripe oocytes present in the ovary or oviducts of females);

and VI = spent (gonad is degraded and of reduced size, the size is roughly as in early maturing individuals or even smaller; residual spermatophores can be present in males; only post-ovulatory follicles and resorbing oocytes present in females).

The products of tentative spermatophorogenesis are treated following Nigmatullin et al*.* [80]: 1) spermatophore-like structures resemble fragments of spermatophores and do not contain sperm; and 2) tentative spermatophores remind normal spermatophores but are smaller and with different proportions, and either do not contain sperm at all or have lowered sperm concentration. In the main text, sex and maturity stage of the respective individual are reported as a sign (♀or ♂) and a roman digit.

Radulae were carefully washed with distilled water, dehydrated using ascending ethanol concentrations (70%, 80%, 90%, 96%, and 100%), CO_2_ critical-point dried and examined using a Hitachi TM Series SEM scanning electron microscope at the Department of Zoology, Kazan Federal University, Kazan. A regression analysis was used to find equations fitting our data [81], with *α* = 0.05 regarded as significant. Analyses were performed in PAST 4.02 [82].

### Barcoding *COI* DNA sequences and analyses

Samples for genetic analyses were only available from single individual of *M. sibiricus* (see ‘Species description’ below for individual’s details). Total DNA was extracted from mantle muscle tissue using a QIAamp DNA Mini Kit (Qiagen GmbH, Hilden, Germany) following manufacturer instructions. Primers used for the *COI* barcode were 5′-TAAACTTCAGGGTGACCAAAAAATCA-3′ and 5′-GGTCAACAAATCATAAAGATATTGG-3′ [83]. The PCR mixture included 12.5 μL of Q5 High-Fidelity 2X Master Mix, 1.25 μL of each primer (10 µM), 7 μL of nuclease-free water and 2 μL of DNA template. Amplification included 30 s denaturation at 98 °C followed by 35 cycles each consisting of 10 s denaturation at 98 °C, 15 s of annealing at temperature of 52 °C and 1 min extension at 72 °C. A final extension was carried out at 72 °C for 5 min. PCR products were electrophoresed on 1% agarose along with appropriate negative controls and DNA ladder. After purification using a QIAquick PCR Purification Kit (Qiagen GmbH, Hilden, Germany), amplicons were sequenced by Sanger Sequencing using ABI Prism 3 500 (Applied Biosystems, CA, USA).

A phylogenetic tree was constructed using the neighbor-joining method in MEGA 11 [84]. All sequences of *Muusoctopus*, *Benthoctopus,* and *Vulcanoctopus* available in GenBank (https://www.ncbi.nlm.nih.gov/genbank) and BOLD (https://www.boldsystems.org/) databases on 18 August 2023 were used; *Octopus vulgaris* (accession number MW560654) was used as an outgroup. All accession numbers are provided on a phylogenetic tree (SM.01 Fig. S1). The most suitable evolutionary model for analysis, as determined in MEGA 11 based on the lowest Bayesian information criterion scores, is Tamura–Nei substitution model with gamma distribution (TN93 + G). Bootstrapping was used to verify the validity of trees constructed based on results of multiple sequence alignment with MUSCLE option. Nodes are supported by 100 bootstrap replicates. Because single gene cladograms are unreliable for phylogenetic inferences, e.g. [85], we use them here solely to differentiate the species.

## Results

### Species descriptions

#### Family

Enteroctopodidae Strugnell, Norman, Vecchione, Guzik & Allcock, 2014 [9].

#### Genus

*Muusoctopus* Gleadall, 2004 [20].

#### *Muusoctopus aegir* Golikov, Gudmundsson & Sabirov, sp. nov.

**Fig. 2:**
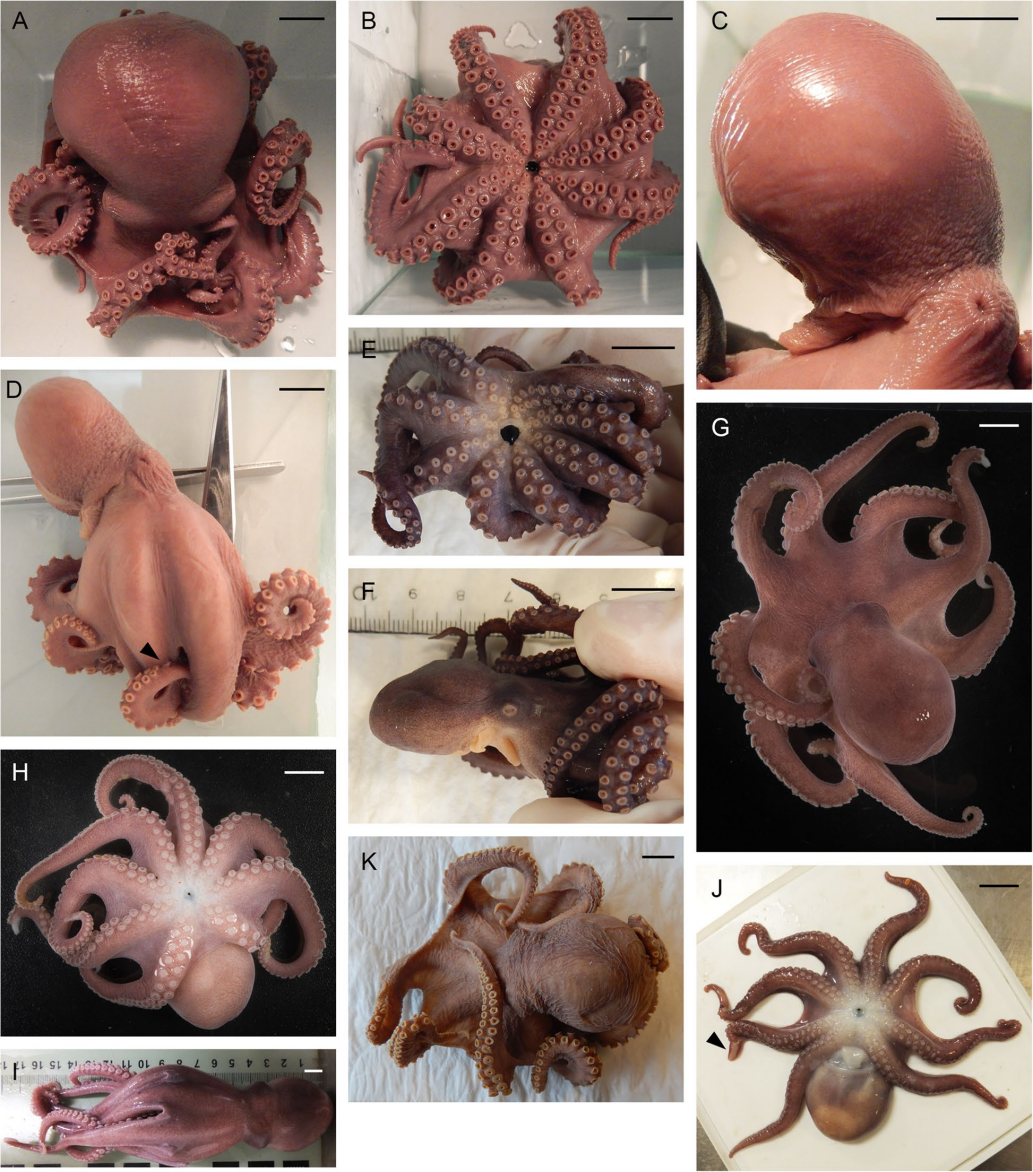
*Muusoctopus aegir* Golikov, Gudmundsson & Sabirov, **sp. nov.** External view. **a–c**, holotype BS-319–2012 (late maturing female, mantle length (ML) 43 mm, off the Barents Sea slope, fixed): dorsal (**a**), ventral (**b**) and lateral (**c**) view; **d**, paratype KS-189–2010 (mature male, ML 30 mm, the Kara Sea, fixed): lateral view; **e**, **f**, paratype ICL-A13-570–2017 (pre-spent female, ML 24 mm, off Iceland, fixed): ventral (**e**) and lateral (**f**) view; **g**, **h**, ICL-A11-640–2016 (not analyzed; off Iceland, fresh): dorsal (**g**) and ventral (**h**) view; **i**, **j**, BS-HH-244–2012 (mature male, ML 36 mm, off Svalbard, fresh (**j**) and fixed (**i**)): dorsal (**i**) and ventral (**j**) view; **k**, BIOICE-2322 (late maturing female, ML 38 mm, off Iceland, fixed): dorsal view. Arrowheads indicate the hectocotylus in males. Scale bars = 10 mm

**Fig. 3:**
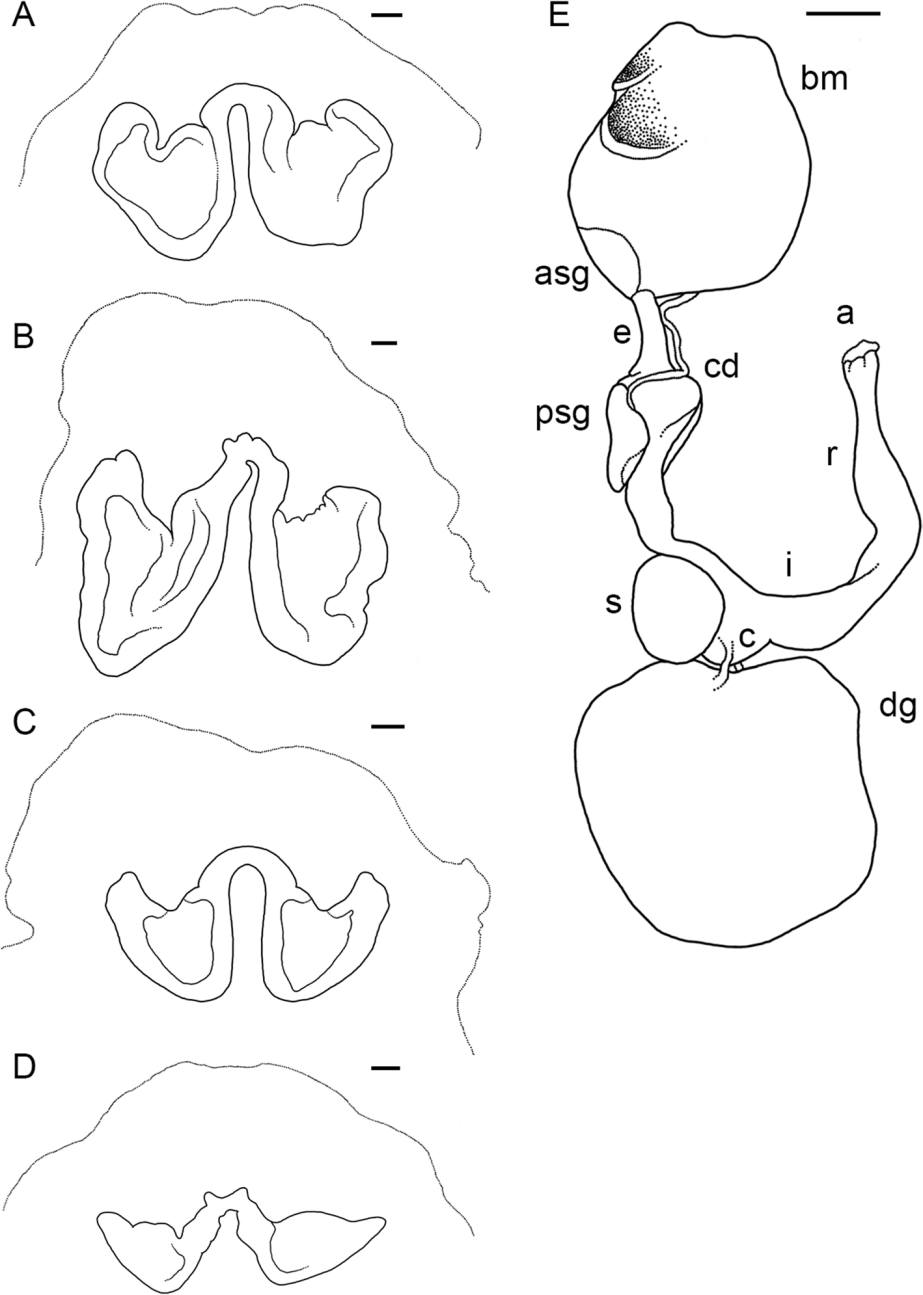
*Muusoctopus aegir* Golikov, Gudmundsson & Sabirov, **sp. nov.** General anatomy. **a**, holotype BS-319–2012 (late maturing female, mantle length (ML) 43 mm, off the Barents Sea slope): funnel organ; **b**, paratype KS-189–2010 (mature male, ML 30 mm, the Kara Sea): funnel organ; **c**, BS-70–2-2018 (late maturing male, ML 24 mm, the Barents Sea): funnel organ; **d**, BIOICE-2516–2 (late maturing female, ML 28 mm, off Iceland): funnel organ; **e**, paratype BS-304–2012 (spent female, ML 26 mm, off the Barents Sea slope): digestive tract, rectum loop untangled. Scale bars: **a**–**d** = 1 mm, **e** = 5 mm. Abbreviations: a, anus; asg, anterior salivary gland; bm, buccal mass; c, caecum; cd, crop diverticulum; dg, digestive gland; e, esophagus; i, intestine; psg, posterior salivary gland; r, rectum; s, stomach

**Fig. 4:**
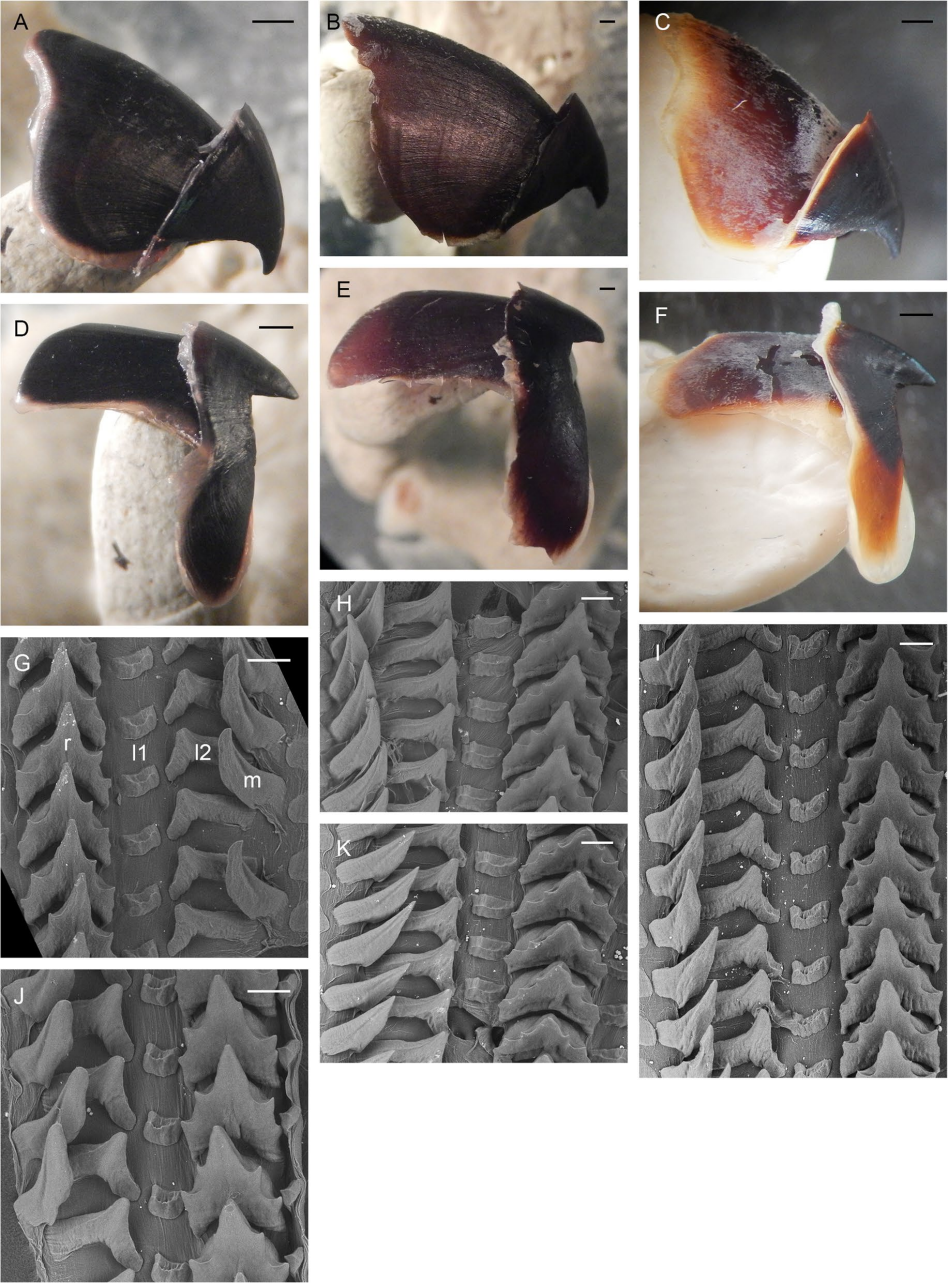
*Muusoctopus aegir* Golikov, Gudmundsson & Sabirov, **sp. nov.** Beak and radula. **a**, **d**, paratype BS-70–1-2018 (pre-mature male, mantle length (ML) 29 mm, the Barents Sea): upper (**a**) and lower (**d**) beaks; **b**, **e**, KS-201–2010 (spent female, ML 52 mm, the Kara Sea): upper (**b**) and lower (**e**) beaks; **c**, **f**, BIOICE-2789 (early maturing male, ML 27 mm, off Iceland): upper (**c**) and lower (**f**) beaks; **g**, **j**, holotype BS-319–2012 (late maturing female, ML 43 mm, off the Barents Sea slope): older (**g**) and younger (**j**) unworn sections of radula; **h**, **k**, BS-255–2010 (mature male, ML 28 mm, off the Barents Sea slope): older (**h**) and younger (**k**) unworn sections of radula; **i**, BIOICE-2516–2 (late maturing female, ML 28 mm, off Iceland): older unworn section of radula. Scale bars: **a–f** = 1 mm, **g–k** = 100 µm. Abbreviations: l1, first lateral tooth; l2, second lateral tooth; m, marginal tooth; r, rachidian tooth

**Fig. 5:**
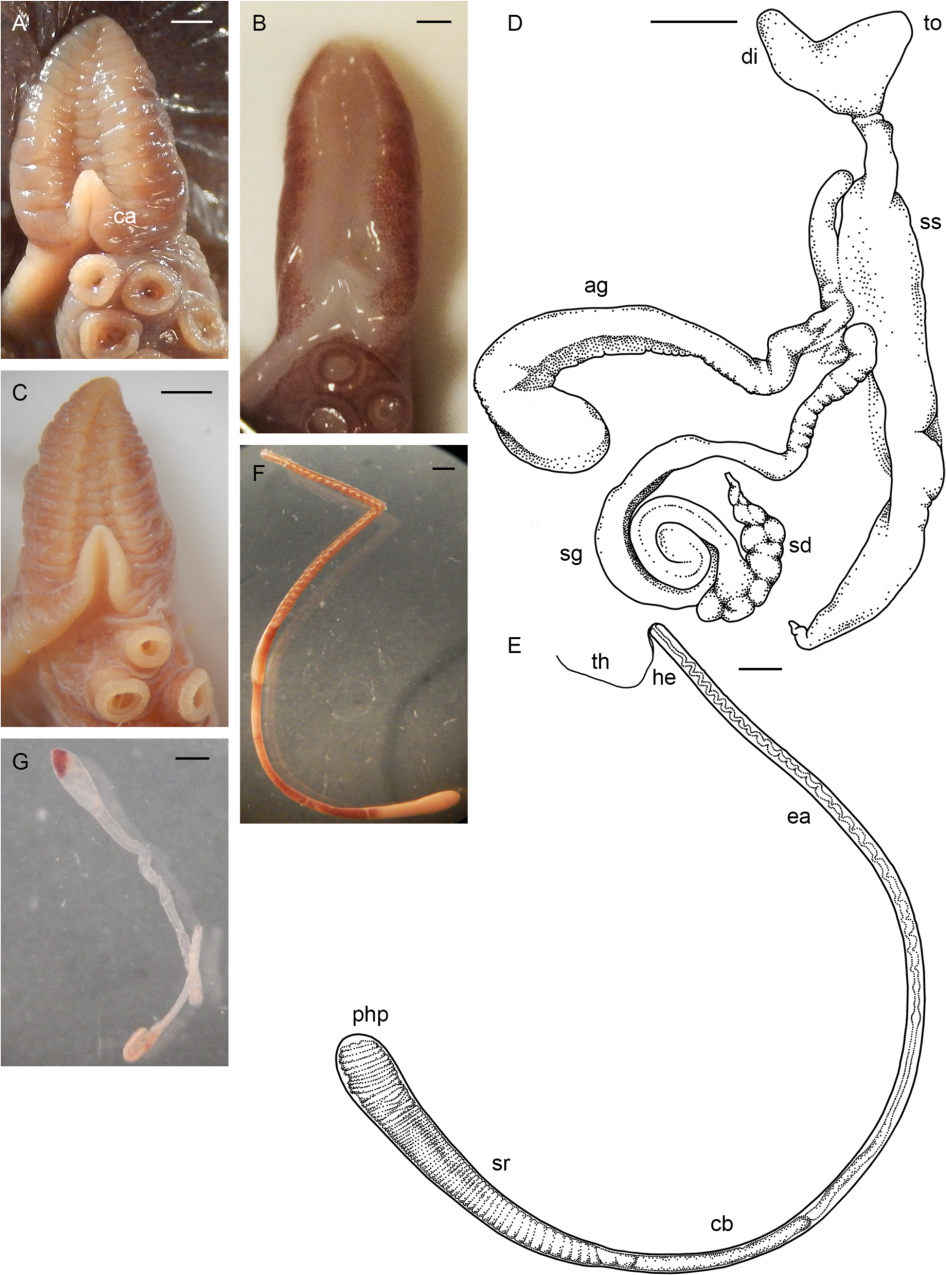
*Muusoctopus aegir* Golikov, Gudmundsson & Sabirov, **sp. nov.** Hectocotylus and male reproductive anatomy. **a**, paratype BS-70–1-2018 (pre-mature male, mantle length (ML) 29 mm, the Barents Sea): hectocotylus; **b**, **d**, **e**, BS-HH-244–2012 (mature male, ML 36 mm, off Svalbard): hectocotylus (**b**), fresh, spermatophoric complex (**d**), dissected, ventral view, and spermatophore (**e**); **c**, BIOICE-2789 (early maturing male, ML 27 mm, Iceland): hectocotylus; **f**, **g**, BS-255–2010 (mature male, ML 28 mm, off the Barents Sea slope): tentative spermatophore (**f**) and spermatophore-like structure (**g**). Scale bars: **a**–**c**, **e**–**g** = 1 mm, **d** = 5 mm. Abbreviations: ag, accessory gland; ca, calamus; cb, cement body, di, diverticulum; ea, ejaculatory apparatus; he, head; sd, sperm duct; sg, spermatophoric glands; sr, seminal reservoir; ss, spermatophoric sac; th, thread; to, terminal organ

**Fig. 6:**
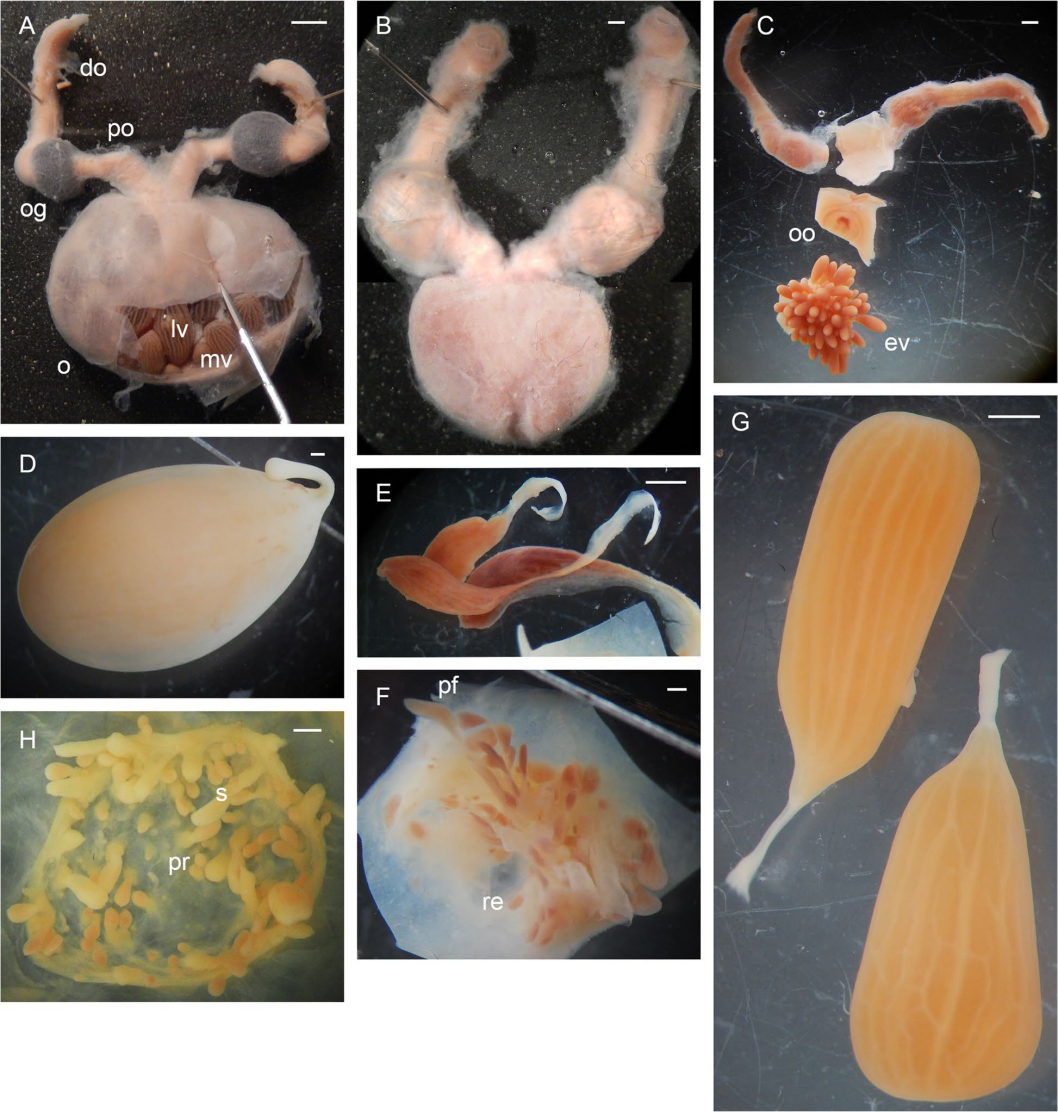
*Muusoctopus aegir* Golikov, Gudmundsson & Sabirov, **sp. nov.** Female reproductive anatomy. **a**, holotype BS-319–2012 (late maturing female, mantle length (ML) 43 mm, off the Barents Sea slope): female reproductive tract; **b**, paratype BS-304–2012 (spent female, ML 26 mm, off the Barents Sea slope): female reproductive tract; **c**, BIOICE-2033 (early maturing female, ML 21 mm, off Iceland): dissected female reproductive tract; **d**–**f**, paratype ICL-A13-570–2017 (pre-spent female, ML 24 mm, off Iceland): ripe ova in shell (**d**), post-ovulatory follicles (**e**) and part of the ovary with post-ovulatory follicles and resorbing oocytes (**f**); **g**, **h**, BIOICE-2516–1 (late maturing female, ML 32 mm, off Iceland): late vitellogenic oocytes (**g**) and part of the ovary with pre-vitellogenic oocytes (**h**). Scale bars: **a** = 5 mm, **b**–**h** = 1 mm. Abbreviations: do, distal oviduct; ev, early vitellogenic oocyte; lv, late vitellogenic oocyte; mv, mid-vitellogenic oocyte; o, ovary; og, oviducal gland; oo, oviduct opening; pf, post-ovulatory follicle; po, proximal oviduct; pr, pre-vitellogenic oocyte; re, resorbing oocyte; s, stalk

(Tables 1, 2, 3, 4 and 5, 9; SM.01 Tables S1, S2; Figs. 2, 3, 4, 5 and 6).

**Table 1:** Data on maturing and mature male individuals of *Muusoctopus aegir* Golikov, Gudmundsson & Sabirov, **sp. nov.** Immature individuals detailed in SM.01 Table S1

**Individual/character**	**BIOICE-** **2789**	**BS-JM-** **539–2009**	**BS-HH-** **244–2012**	**BS-HH-** **264–2012**	**BS-255-** **2010**	**BS-70-** **1-2018**^a^	**BS-70-** **2–2018**	**BS-176-** **2017**	**KS-189-** **2010**^a^	**KS-27-** **1–2007**	**KS-15-** **1–2007**
Area	ICL	BS	BS	BS	BS	BS	BS	BS	KS	KS	KS
Maturity stage	Early maturing (III)	Mature (V_2_)	Mature (V_2_)	Mature (V_2_)	Mature (V_2_)	Pre-mature (V_1_)	Late maturing (IV)	Early maturing (III)	Mature (V_2_)	Mature (V_2_)	Pre-mature (V_1_)
ML, mm	27	50	36	46	28	29	24	22	30	46	32
TL, mm	126	n/a	n/a	n/a	134	152	118	102	167	235	139
Ventral ML, mm	26	49	n/a	n/a	21	26	21	18	28	40	28
Mantle width, mm	28	n/a	n/a	n/a	34	32	29	24	40	41	33
Head length, mm	13	n/a	n/a	n/a	12	14	10.5	9	15.5	18	12
Head width, mm	22	n/a	n/a	n/a	25	24	21	18	23	32	26
Eye diameter, mm	11.0	n/a	n/a	n/a	10.0	9.0	7.5	7.5	12.0	16.0	9.0
Lens diameter, mm	2.5	n/a	n/a	n/a	2.8	3.5	2.2	2.0	4.1	4.3	3.2
Funnel length, mm	11.0	n/a	n/a	n/a	10.0	13.5	12.0	9.0	17.0	18.0	14.0
Free funnel length, mm	6.0	n/a	n/a	n/a	5.3	7.5	6.6	4.5	9.0	10.1	7.4
Web depth, mm (min – max)	n/a	n/a	n/a	n/a	17–28	20–26	19–26	14–20	33–36	32–44	24–29
Web formula	n/a	n/a	n/a	n/a	c > b = d > a > e	b > a > c > d > e	b > c > a > d > e	a = c > b = d > e	a = c > b > d > e	c > b = d > a > e	a > b > d > c > e
Arm length, mm (min – max)	82–86	147^b^	115–117	168–170	89–94	86–104	76–83	67–71	101–111	162–171	86–95
Arm formula	2 = 3 > 1 > 4	n/a	1 > 2 = 3 > 4	1 = 2 > 3 = 4	2 > 1 = 3 > 4	2 > 1 > 3 > 4	1 = 2 > 3 > 4	1 = 2 > 3 > 4	2 > 3 > 1 = 4	1 > 2 > 3 > 4	1 > 2 > 3 > 4
Sucker count (min – max)	94–102	n/a	108–120	n/a	84–90	90–98	84–88	90–98	92–100	100–110	96–100
Sucker diameter (max), mm	3.0	n/a	n/a	n/a	2.5	2.5	2.5	1.7	3.5	3.0	3.3
Gill length, mm	9.0	n/a	n/a	n/a	8.5	9.0	9.0	9.0	10.5	18.0	9.5
Gill lamellae count, outer/inner	8/7	n/a	n/a	n/a	8/7	8/8	8/7	8/7	9/8	8/7	8/7
Hectocotylized arm length, mm	62	113	64	130	63	67	61	50	73	113	71
Hectocotylized arm sucker count	56	n/a	54	n/a	46	54	52	50	54	54	48
Ligula length, mm	5.5	11.0	9.0	11.0	7.0	6.0	5.8	4.0	9.0	12.0	8.0
Ligula width, mm	3.0	n/a	5.0	6.6	4.4	3.8	3.6	1.8	4.8	6.0	3.8
Calamus length, mm	2.2	n/a	3.8	3.9	2.9	1.8	2.1	1.1	3.2	5.2	2.9
Number of spermatophores	Normal spermatophores absent^d^	11	13	22	7^c^	5	Normal spermatophores absent	Spermatophores absent	20	17	5
Spermatophore length, mm (min – max)		39.0^e^	36.0–39.1	41.0–45.4	28.1–38.0	35.3–39.4			30.3–40.5	41.00–48.0	27.9–34.9

*ML* mantle length, *TL* total length, *ICL* Iceland, *BS* Barents Sea, *KS* Kara Sea, *n/a* not analyzed

^a^paratype; ^b^third left arm measured only

^c^also had three tentative spermatophores, not measured

^d^one tentative spermatophore, length 20.9 mm

^e^one spermatophore measured only

^f^one fragmented tentative spermatophore

##### Synonymy

*Octopus piscatorum* Verrill, 1879 [30] – Hoyle, 1886 [34]: 91 (partim); Lønnberg, 1892 [35]: 8 (partim); Appelløf, 1893 [36]: 3 (partim).

**Fig. 7:**
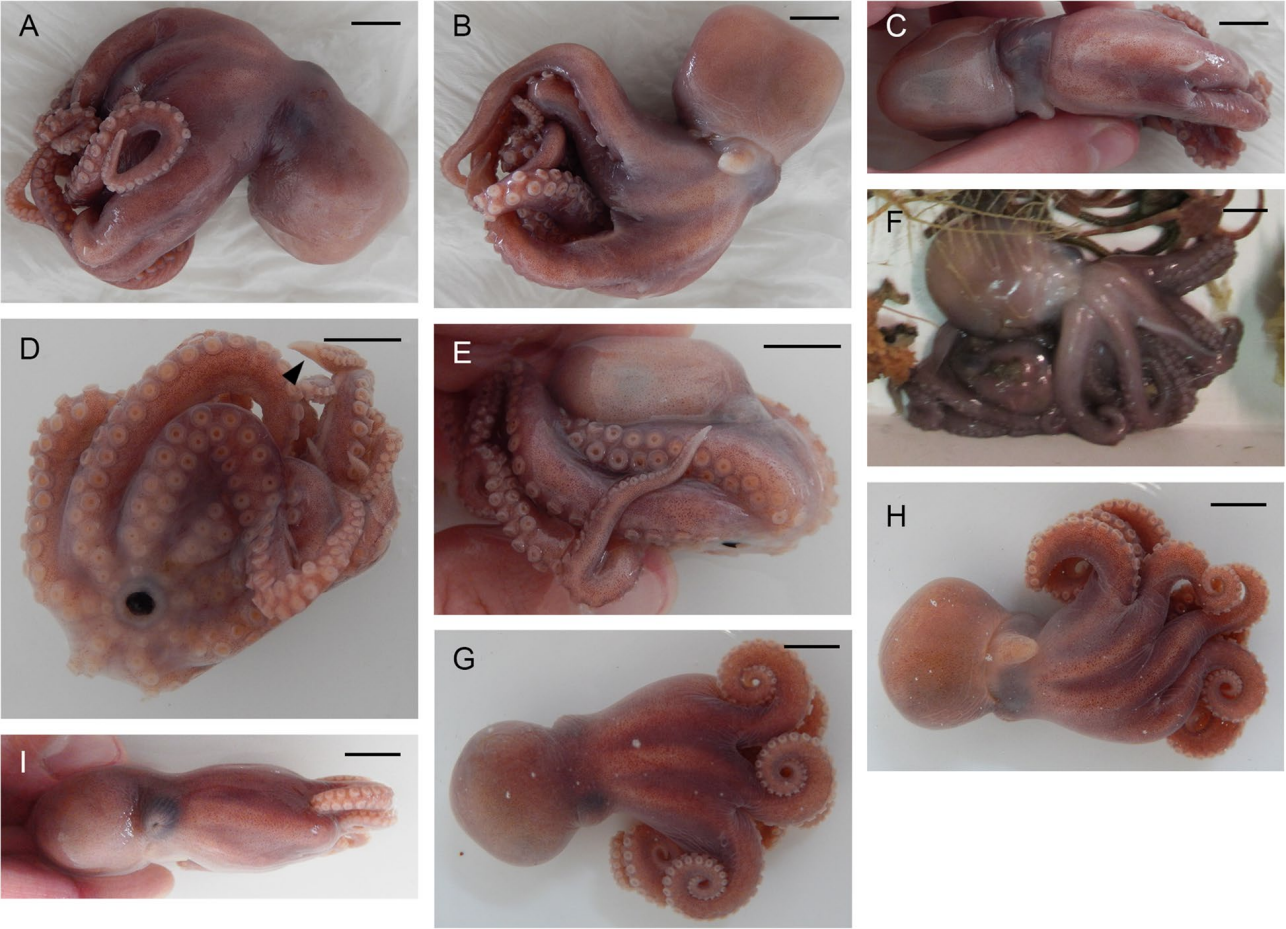
*Muusoctopus* sp. 1. External view. **a**–**c**, GRL-PA-7–20-1–2016 (late immature male, mantle length (ML) 30 mm, northern Baffin Bay, fixed): dorsal (**a**), ventral (**b**) and lateral (**c**) view; **d**, **e**, GRL-PA-7–20-2–2016 (early immature male, ML 23 mm, northern Baffin Bay, fixed): ventral (**d**) and lateral (**e**) view; **f**, GRL-PA-7–20-1–2016 and GRL-PA-7–20-2–2016 in bottom trawl catch, showing fresh coloration; **g**–**i**, GRL-7–117-2016 (early immature female, ML 22 mm, northern Baffin Bay, fixed): dorsal (**g**), ventral (**h**) and lateral (**i**) view. Arrowhead indicates the hectocotylus in male. Scale bars = 10 mm

*Polypus piscatorum* (Verrill, 1879) [30] – Pfeffer, 1908 [40]: 19, Figs 9, 10 (partim); Russel, 1909 [41]: 446 (partim); Russel, 1922 [42]: 7, pl. II Fig 7 (partim).

*Benthoctopus piscatorum* (Verrill, 1879) [30] – Robson, 1932 [43]: 224, figs 31, 34, 35 (partim); Grieg, 1933 [44]: 8 (partim); Grimpe, 1933 [45]: 496 (partim); Stephen, 1944 [46]: 253 (partim); Muus, 1959 [47]: 218, fig. 111 (partim); Nesis, 1987a [49]: 316, figs 84G, 84H (partim); Nesis, 1987b [50]: 124 (partim); Nesis, 2001 [51]: 7, fig. 4 (partim).

*Muusoctopus* sp. – Golikov et al., 2018 [75]: 1; Xavier et al., 2018 [86]: 5; Taite et al. (in press): 8 (in draft).

Not *Octopus piscatorum* Verrill, 1879 [30]: 470; Verrill 1881 [87]: 377, pl. XXXVI figs. 1, 2; Verrill 1884 [88]: 248; Verrill 1885 [89]: pl. XLII fig 5.

Not *Polypus normani* Massy, 1907 [37]: 379.

Not *Polypus piscatorum* (Verrill, 1879) [30] – Massy, 1909 [38]: 13, pl. II figs. 2–4.

Not *Benthoctopus piscatorum* (Verrill, 1879) [30] – Massy, 1928 [39]: 27; Aldrich & Lu, 1968 [48]: 70, pl. 8 figs 1, 2; Nixon, 1991 [53]: 499; O’Shea, 1999 [77]: 192, figs 115A, 115B; Collins et al. 2001 [22]: 112; Nixon & Young, 2003 [54]: 321, figs 31.58, 31.59,31.61, 31.62.

Not *Benthoctopus* sp. – Collins et al*.* 2001 [22]: 112; Barrat et al*.* 2007 [90]: 392.

##### ZooBank

urn:lsid:zoobank.org:act:7110DA04-B684-452D-B285-24B38A63E013.

##### Material examined

Holotype: ZM KFU ZMG SC-9 INV-1 (ЗMиГ КП-9 БП-1): ♀IV, ML 43 mm, BS-319–2012, Stn 319, 82.07°N, 40.42°E, 677 m, bottom temperature (BT) 0.44 °C, 22 September 2012. Paratypes: ZM KFU ZMG SC-9 INV-2 (ЗMиГ КП-9 БП-2): ♀VI, ML 26 mm, BS-304–2012, Stn 304, 80.77°N, 44.02°E, 280.5 m, BT 0.31 °C, 20 September 2012; ZIAS ZIN 1/306 522–2022: ♂V_1_, ML 29 mm, BS-70–1-2018, Stn 70, 79.26°N, 52.32°E, 321 m, BT –0.27 °C, 27 September 2018; ZM KFU ZMG SC-9 INV-3 (ЗMиГ КП-9 БП-3): ♂V_2_, ML 30 mm, KS-189–2010, Stn 189, 81.82°N, 75.92°E, 367 m, BT –1.26 °C, 9 September 2010; IINH 37,493: ♀V_3_, ML 24 mm, ICL-A13-570–2017, Stn 570, 67.80°N, 19.15°W, 793.5 m, 21 October 2017.

##### Other material examined

Iceland: IINH 37,824, ♂III, ML 27 mm, BIOICE Stn 2789, 67.31°N, 18.39°W, 535 m, BT –0.29 °C, 5 August 1995; IINH 37,828, ♂I, ML 12 mm, BIOICE Stn 2369, 64.67°N, 9.57°W, 970 m, 8 May 1993; IINH 37,819, 2♀IV, ML 32, 28 mm, BIOICE Stn 2516, 66.62°N, 25.39°W, 683 m, BT –0.50 °C, 13 July 1993; IINH 37.821, ♀IV, ML 38 mm, BIOICE Stn 2322, 63.92°N, 10.06°W, 628 m, 3 May 1993; IINH 37,820, ♀III, ML 29 mm, BIOICE Stn 2326, 63.73°N, 10.15°W, 563 m, BT –0.48 °C, 3 May 1993; IINH 37,822, ♀III, ML 21 mm, BIOICE Stn 2033, 66.91°N, 13.50°W, 556.5, BT –0.54 °C, 23 July 1991; IINH 37,825, ♀II, ML 19 mm, BIOICE Stn 3124, 68.16°N, 17.99°W, 875 m, BT 0.33 °C, 22 August 1999; IINH 37,823, ♀II, ML 16 mm, BIOICE Stn 3242, 66.22°N, 11.97°W, 418 m, BT –0.20 °C, 14 July 2001; IINH 37,827, ♀I, ML 8 mm, juvenile (sex indet.), ML 4.5 mm, BIOICE Stn 3659, 67.79°N, 19.61°W, 800 m, BT –0.53 °C, 24 July 2004.

Barents Sea (Laboratory of Hydrobiology (LH), Department of Zoology, KFU): ♂V_2_, ML 50 mm, BS-JM-539–2009, Stn 539, 80.22°N, 5.70°E, 704 m, BT –0.55 °C, 15 September 2009; ♂V_2_, ML 36 mm, BS-HH-244–2012, Stn 244, 78.49°N, 9.01°E, 534 m, 23 August 2012; ♂ V_2_, ML 46 mm, BS-HH-264–2012, Stn 264, 80.03°N, 8.44°E, 495 m, 25 August 2012; ♂V_2_, ML 28 mm, BS-255–2010, Stn 255, 81.05°N, 44.23°E, 343 m, BT –0.28 °C, 19 October 2010; ♂IV, ML 24 mm, BS-70–2-2018, Stn 70, 79.26°N, 52.32°E, 321 m, BT –0.27 °C, 27 September 2018; ♂III, ML 22 mm, BS-176–2017, Stn 176, 78.47°N, 44.74°E, 226.5 m, BT 0.67° C, 25 September 2017; ♀VI, ML 25 mm, BS-305–2012, Stn 305, 81.25°N, 44.83°E, 183.5 m, BT 0.34 °C, 20 September 2012; ♀VI, ML 36 mm, BS-246–2010, Stn 246, 79.75°N, 42.70°E, 354 m, 18 September 2010; ♀VI, ML 20 mm, BS-322–2014, Stn 322, 82.84°N, 50.41°E, 529 m, 23 September 2014; ♀V_2_, ML 44 mm, BS-HH-259–1-2012 and ♀II, ML 31 mm, BS-HH-259–2-2012, Stn 259, 79.87°N, 6.76°E, 834 m, 24 August 2012; ♀II, ML 31 mm, BS-HH-269–2012, Stn 269, 80.45°N, 4.80°E, 730 m, 25 August 2012.

Kara Sea (LH KFU): ♂V_2_, ML 46 mm, KS-27–1-2007, ♂II, ML 29 mm, KS-27–2-2007, ♀II, ML 21 mm, KS-27–3-2007, Stn 27, 75.98°N, 71.90°E, 201 m, BT –1.08 °C, 21 September 2007; ♂V_1_, ML 32 mm, KS-15–1-2007, ♂II, ML 19 mm, KS-15–2-2007, Stn 15, 79.64°N, 73.51°E, 423 m, BT –0.28 °C, 17 September 2007; ♂II, ML 27 mm, KS-25–2007, Stn 25, 76.95°N, 70.93°E, 429.5 m, BT 0.07 °C, 20 September 2007; ♀VI, ML 52 mm, KS-201–2010, Stn 201, 78.90°N, 69.95°E, 490.5 m, 11 September 2010; ♀VI, ML 30 mm, KS-28–2009, Stn 28, 80.33°N, 73.47°E, 398 m, BT –0.69 °C, 18 August 2009; ♀II, ML 17 mm, KS-16–2007, Stn 16, 78.80°N, 74.08°E, 383.5 m, BT –0.25 °C, 17 September 2007.

##### Additional material examined

See SM.01.

##### Type locality

Off the Barents Sea slope, Stn 319, 82.07°N, 40.42°E, 677 m, BT 0.44 °C.

##### Etymology

Named after Ægir (Old Norse for ‘sea’, latinized and anglicized to ‘aegir’; noun in apposition, masculine), a sea giant associated with the ocean in Norse mythology [91], because the distribution of this species extends along the slope of Scandinavia and Iceland, and to underscore the senior author’s appreciation of ancient Norse culture, history, and mythology.

##### Diagnosis

Small (maximum ML 52 mm) violet-brown octopods, paler ventrally, and with white area orally. Skin smooth, body rounded, arms ~ 3.1 times ML. Suckers closely set, small, not enlarged in either sex. From 84 to 120 suckers on unmodified arms, and 46–56 suckers on hectocotylus. Ligula moderately large and broad, tapering gradually, without transverse ridges, but with 8–14 low indistinct rugae; calamus large, pointed. Funnel of moderate length, free from ventral surface of head for slightly more than half its length. Funnel organ W-shaped, with medial and marginal limbs of the same length (or medial limbs are slightly longer), and with broad marginal limbs. Gills long, with eight or nine outer and seven or eight inner lamellae per demibranch. Stylets, anal flaps, ink sac, and ink duct absent. Multicuspid rachidian with 5–7 cusps, located asymmetrically with seriation of 4–6. Long and slender spermatophores, up to 22 (mean: 13 ± 2). Female with up to 168 oocytes (mean: 100 ± 7).

##### Description

Counts and measurements for the species are given in Tables 1, 2 and 3 and SM.01 Table S1, and indices are given in Table 9.

Description based on 25 individuals (all studied individuals, excluding the immature ones, i.e., 11 males (♂) and 14 females (♀)), additionally data for 2♂ (late maturing and mature) are from Nesis [51]. Species small, ML 20–52 mm (32.3 ± 1.8 mm), total length (TL) 96–235 mm (141.6 ± 7.8 mm) (Fig. 2; Tables 1, 2, 9); ventral ML 1–10 mm shorter than dorsal ML. Mantle wider than long, appearing round; width 111.3% ± 3.4% ML. Head narrower (71.3% ± 2.2%) than mantle (Fig. 2). Eyes relatively prominent in comparison to other North Atlantic and Arctic *Muusoctopus* (Table 9); diameter 32.5% ± 0.8% ML (Fig. 2). Funnel moderately long (42.1% ± 1.6% ML), tapered. Funnel free from ventral surface of head for slightly more than a half its length (mean 52.9% ± 0.7% funnel length). Funnel organ W-shaped, with medial and marginal limbs of similar length, or with medial limbs slightly longer; marginal limbs broad (Fig. 3a–d). Arms relatively long, ~ 3.1 times ML (Fig. 2), their length subequal, of formula typically 1.2.3.4. Suckers: number 84–120 (95.5 ± 1.5) per arm, biserial from base of arms to arm tips, small (8.9% ± 0.3% ML), closely set (Fig. 2); none enlarged in either sex. Web medium deep (25.5% ± 1.1% longest arm length); all web sectors are approximately subequal, with sectors B and C deepest, and D and E most shallow.

**Table 2:** Data onmaturing, mature and spent female individuals of *Muusoctopus aegir* Golikov, Gudmundsson & Sabirov, **sp. nov.** Immature individuals are detailed in SM.01 Table S1

**Individual/character**	**ICL-A13-** **570-2017**^a^	**BIOICE-** **2516–1**	**BIOICE-** **2516–2**	**BIOICE-** **2322**	**BIOICE-** **2326**	**BIOICE-** **2033**	**BS-304-** **2012**^a^	**BS-305-** **2012**	**BS-246-** **2010**	**BS-322-** **2014**	**BS-HH-** **259–2012-1**	**BS-319-** **2012**^b^	**KS-201-** **2010**	**KS-28-** **2009**
Area	ICL	ICL	ICL	ICL	ICL	ICL	BS	BS	BS	BS	BS	BS	KS	KS
Maturity stage	Pre-spent (V_3_)	Late maturing (IV)	Late maturing (IV)	Late maturing (IV)	Early maturing (III)	Early maturing (III)	Spent (VI)	Spent (VI)	Spent (VI)	Spent (VI)	Mature (V_2_)	Late maturing (IV)	Spent (VI)	Spent (VI)
ML, mm	24	32	28	38	29	21	26	25	36	20	44	43	52	30
TL, mm	101	135	114	171	137	110	133	122	153	96	n/a	173	218	138
Ventral ML, mm	19	26	24	35	28	20	24	21	31	17	n/a	33	48	28
Mantle width, mm	25	28	28	34	27	25	35	31	40	28	n/a	54	51	36
Head length, mm	10	12	12	17	12	10	13	12	11	8	n/a	14	22	10
Head width, mm	12	24	20	29	23	14	25	22	26	20	n/a	32	31	28
Eye diameter, mm	7.0	11.0	8.0	13.0	10.0	7.0	8.0	7.5	9.5	7.0	n/a	12.0	16.5	9.0
Lens diameter, mm	1.5	2.5	2.0	3.0	2.0	1.4	2.5	2.3	3.2	2.2	n/a	4.5	5.5	2.9
Funnel length, mm	9.5	10.5	12.0	14.0	11.0	11.0	14.0	13.0	14.0	8.0	n/a	15.0	15.0	12.0
Free funnel length, mm	5.0	5.0	6.0	8.0	5.5	5.0	7.5	7.2	7.5	4.0	n/a	8.5	8.5	6.2
Web depth, mm (min – max)	7–11	16–26	12–18	22–30	n/a	16–18	25–30	25–28	19–33	14–20	n/a	28–37	34–41	22–26
Web formula	a = b = c > d > e	a = b > c = d > e	a > b = c > d > e	a > b = c > d > e	n/a	a = b > c = e > d	b > a = c > d > e	b > a = c = e > d	b > c > a > d > e	a > b = c = d > e	n/a	a = b = c > d > e	b = c > c > a > e	b = c = d > a > e
Arm length, mm (min – max)	61–67	84–91	79–84	98–116	92–96	70–79	85–91	79–83	98–106	64–68	n/a	107–116	122–144	92–98
Arm formula	2 > 3 > 4 > 1	1 = 2 = 3 > 4	1 > 2 > 3 > 4	2 > 1 > 3 > 4	1 > 2 = 3 = 4	1 > 2 > 3 > 4	1 > 3 > 2 > 4	1 > 2 > 3 > 4	1 > 2 > 3 > 4	1 = 2 > 3 > 4	n/a	1 = 2 > 3 > 4	1 > 2 > 3 > 4	1 = 2 > 3 > 4
Sucker count (min – max)	94–96	88–92	84–90	90–96	92–98	96–100	88–98	86–90	100–104	88–100	n/a	102–106	92–102	84–96
Sucker diameter (max), mm	1.5	2.5	2.5	3.5	3.0	2.0	2.2	2.0	3.1	1.4	n/a	4.0	4.0	3.0
Gill length, mm	8.0	7.0	8.0	11.5	12.0	9.0	9.5	11.0	10.5	8.0	n/a	16.5	12.0	10.5
Gill lamellae count, outer/inner	8/8	8/7	8/7	8/7	8/8	9/8	9/8	8/7	9/8	8/7	n/a	8/7	8/7	8/7
Fecundity	71^c^	121	101	168	112	130	74	89	93	65	n/a	78	73	93

*ML* mantle length, *TL* total length, *ICL* Iceland, *BS* Barents Sea, *KS* Kara Sea, *n/a* not analyzed

^a^paratype

^b^holotype

^c^this is the only individual for which ripe oocyte length is available, = 12.8 mm

Gills long (34.4% ± 1.3% ML), with eight or nine (mode: 8) outer and seven or eight (mode: 7) inner lamellae per demibranch. Stylets absent. Upper beak with hooked rostrum (Fig. 4a–c); lower beak with straight rostrum (Fig. 4d–f); both typically *Muusoctopus*. Anterior salivary glands moderate (21.5% ± 2.6% ML), discoid. Posterior salivary glands large (28.8% ± 2.8% ML), almost triangular. Crop diverticulum well developed (Fig. 3e). Rectum with a loop. Ink sac, ink duct and anal flaps absent (Fig. 3e). Radula with nine elements per transverse row; rachidian with 5–7 cusps, the central largest, with asymmetric lateral cusps with 4 –6 seriation (Fig. 4g–k). Marginal and lateral teeth unicuspid, marginal teeth curved. Marginal plates well developed (Fig. 4g–k).

Male third right arm hectocotylized (Fig. 2d, j), length 233.8% ± 6.5% ML, 71.5% ± 2.2% that of the opposite arm, with 46 to 56 (52.0 ± 1.1) suckers. Ligula moderately large, 8.0–14.1% (10.3% ± 0.5%) of hectocotylized arm length, broad, 45.0–63.3% (55.4% ± 2.1%) ligula length, tapering gradually, with distinct margins and well-marked shallow, narrow groove without transverse ridges, but with 8–14 low indistinct rugae (Fig. 5a–c). Calamus large, 27.5–43.3% (36.8% ± 1.4%) ligula length, pointed (Fig. 5a–c). Spermatophoric complex accessory gland longer than spermatophoric sac (Fig. 5d), both longer than ML. Length of terminal organ with diverticulum 30–56% ML. Spermatophoric sac with 5–22 (12.5 ± 2.4) spermatophores (Tables 1, 3); spermatophores long, 27.9–48.0 mm (39.8 ± 0.5 mm) and 78.0–135.9% (108.1% ± 1.8%) ML, slender (Fig. 5e), of width 0.8–1.4 (1.2 ± 0.04) mm. Sperm cord width 0.1–0.2 mm, forming 65–98 (77.2 ± 2.7) whorls. Seminal reservoir length 26.1–36.4% (31.4% ± 0.6%) spermatophore length (Table 3); ejaculatory tube comprises longest part of spermatophore (Fig. 5e; Table 3). Oviducal glands large (length 17.0% ± 2.0% ML and width 17.8% ± 2.4% ML), broader than long, dark-colored, but paler in spent females (Fig. 6a–c). Fecundity 65–168 (99.5 ± 6.8) oocytes (Tables 2, 4; SM.01 Table S1). Ripe oocyte length 12.5 and 13.0 mm (*n* = 2; in pre-spent female), and 13.0 and 14.0 mm in capsules, respectively (Fig. 6d; Table 4). Large vitellogenic oocytes range from 6.0 to 12.0 mm with 17–24 follicular folds (Fig. 6g; Table 4).

**Table 3:** Spermatophore number and measurements in *Muusoctopus aegir* Golikov, Gudmundsson & Sabirov, **sp. nov.** and *M. sibiricus* (Løyning, 1930) [62]. Values are minimum – maximum (mean ± SE), where applicable

Species/measurement, index or count		*Muusoctopus aegir* sp. nov.	*Muusoctopus sibiricus*
**# spermatophores**		5–22 (12.5 ± 2.4); 7–22 (15.0 ± 2.3) excluding V_1_ males	49 (V_2_), 52 (V_3_)
**SL**	**mm**	27.9–48.0 (39.8 ± 0.5)	46.3–63.5 (57.9 ± 1.1)
	**% ML**	78.0–135.9 (108.1 ± 1.8)	118.7–167.1 (150.6 ± 3.3)
**Spermatophore W**	**mm**	0.8–1.4 (1.2 ± 0.04)	0.6–0.8 (0.7 ± 0.03)
	**% ML**	2.8–3.8 (3.1 ± 0.1)	1.1–1.3 (1.2 ± 0.03)
**Head L**	**mm**	0.7–2.6 (1.5 ± 0.1)	0.8–1.3 (1.1 ± 0.1)
	**% SL**	2.4–6.7 (4.0 ± 0.3)	1.3–2.8 (1.9 ± 0.3)
**Ejaculatory apparatus L**	**mm**	14.0–26.2 (19.4 ± 0.7)	26.3–38.2 (32.6 ± 2.5)
	**% SL**	44.1–58.1 (52.5 ± 0.9)	49.6–61.6 (57.0 ± 2.2)
**Cement body L**	**mm**	2.5–7.6 (4.3 ± 0.3)	5.5–6.8 (6.3 ± 0.2)
	**% SL**	6.7–21.5 (11.7 ± 0.8)	10.4–14.7 (11.2 ± 0.7)
**Seminal reservoir L**	**mm**	9.2–15.4 (11.6 ± 0.4)	11.8–21.0 (16.7 ± 1.3)
	**% SL**	26.1–36.4 (31.4 ± 0.6)	25.5–37.4 (29.5 ± 2.3)
**Seminal reservoir W**	**mm**	0.7–1.3 (1.1 ± 0.04)	0.55–0.70 (0.63 ± 0.03)
	**% SL**	2.5–3.5 (2.9 ± 0.1)	1.0–1.2 (1.1 ± 0.03)
**Seminal reservoir volume, mm**^**3**^		3.6–20.4 (10.9 ± 1.0)	2.8–6.2 (5.2 ± 0.6)
**# of sperm cord whorls**		65–98 (77.2 ± 2.7)	59–92 (79.2 ± 5.8)
**Sperm cord W, mm**		0.10–0.20 (0.16 ± 0.01)	0.15–0.20 (0.19 ± 0.01)
**Posterior hollow part**	**mm**	0.1–0.2 (0.11 ± 0.1)	0.1–0.3 (0.2 ± 0.04)
	**% SL**	0.2–0.5 (0.3 ± 0.02)	0.2–0.5 (0.3 ± 0.1)

*ML* mantle length, *SL* spermatophore length, *L* length, *W* width

**Table 4:** Fecundity and oogenesis in *Muusoctopus aegir* Golikov, Gudmundsson & Sabirov, **sp. nov.**, *M. johnsonianus* (Allcock, Strugnell, Ruggiero & Collins, 2006) [26] and *M. sibiricus* (Løyning, 1930) [62]. Values are minimum – maximum (mean ± SE), where applicable

Species/maturity stages and characters		*Muusoctopus* *aegir* sp. nov.	*Muusoctopus johnsonianus*	*Muusoctopus sibiricus*
**Fecundity (all stages)**		65–168 (99.5 ± 6.8)	227–300 (259.0 ± 21.5)	136
**Realized fecundity, %**		10.8–58.1 (44.7 ± 6.2); 39.4–58.1 (50.4 ± 3.0) if outlier is excluded	No data	No data
**Early** **Immature (I)**	**Fecundity**	96	No data	136
	**Oocytes**	Only pre-vitellogenic, 0.4–0.6 mm		Only pre-vitellogenic, 0.2–0.3 mm
**Late** **immature (II)**	**Fecundity**	98 and 130	250 and 300	No data
	**Oocytes**	Only pre-vitellogenic, 0.4–1.6 mm	Only pre-vitellogenic, 0.3–0.6 mm	
**Early** **maturing (III)**	**Fecundity**	112 and 130	No data	No data
	**Oocytes**	Pre-vitellogenic oocytes: ~ 61%; ~ 57% of them 0.4–0.7 mm and ~ 43% of them 0.9–1.1 mm; Small vitellogenic oocytes: ~ 2%; 2.2–2.5 mm; Medium vitellogenic oocytes: ~ 37%; 2.6–4.5 mm		
**Late** **maturing (IV)**	**Fecundity**	78–168 (117.0 ± 19.1)	227	No data
	**Oocytes**	Pre-vitellogenic oocytes: 0–63%; 18–75% of them 0.2–0.5 mm and 25–82% of them 0.6–1.2 mm; Small vitellogenic oocytes: 7–39%; 2.5–5.0 mm; Medium vitellogenic oocytes: 12–34%; 3.0–7.0 mm; Large vitellogenic oocytes: 0.2–44%; 6.0–12.0 mm; 17–24 folds; Resorbing oocytes: 0.6–28%; 0.5–4.8 mm	Small vitellogenic oocytes: 47%; 2.0–5.0 mm; Medium vitellogenic oocytes: 11%; 10.0–13.0 mm; Large vitellogenic oocytes: 42%; 17.0–22.0 mm; 18–20 folds	
**Pre-mature (V**_**1**_**)**	**Fecundity**	No data	No data	No data
	**Oocytes**			
**Mature (V**_**2**_**)**	**Fecundity**	No data	No data	No data
	**Oocytes**	Analyzed onboard: only ripe oocytes counted (18)		
**Pre-spent (V**_**3**_**)**	**Fecundity**	71	No data	No data
	**Oocytes**	Ripe oocytes: 2.8%; 12.5 and 13.0 mm without capsules, 13.0 and 14.0 mm in capsules; Post-ovulatory follicles: 37%; 1.1–4.2 mm; Resorbing oocytes: 61%; 0.5–3.0 mm		
**Spent (VI)**	**Fecundity**	65–93 (81.2 ± 4.9)	No data	No data
	**Oocytes**	Post-ovulatory follicles: 11–58% (without outlier 44–58%); 2.0–5.1 mm; Resorbing oocytes: 42–89% (without outlier 42–56%); 0.2–2.6 mm		

Skin smooth, without papillae, and minute folds can appear in some fixed individuals (Fig. 2). Live color violet-brown, paler ventrally, and white around the mouth (Fig. 2e–j). Some fixed individuals turn darker, while others turn lighter of which some eventually lose color.

##### Distribution

In the Faroe–Shetland Channel, along the northern slope of the Greenland–Iceland–Faroe Ridge, farther to the east along the Norwegian slope and the continental slopes of the Barents and Kara Seas (Fig. 1): the easternmost location is to the north of the Severnaya Zemlya Archipelago, 96.94°E [34–36, 40–47, 49–52, 57] [the present study]. The species enters the Barents and Kara Seas via deep-sea troughs (Fig. 1). It is not known how far north the distribution of *M. aegir* extends along the East Greenland slope. Habitat depth is 86–2000 m judging from literature, with the associated bottom temperature –0.9° C [34, 36, 41, 42, 44, 51, 57], and 86–2442 m (579.4 ± 52.4 m) and –1.31–6.90 °C (0.41 ± 0.30 °C), respectively, according to our data.

##### Biology and ecology

Among the characters studied, only the following increase without significant correlation to ML in *M. aegir*: relative width of mantle and ligula; relative diameter of eye and sucker; relative length of arm, gill, hectocotylized arm, opposite arm, ligula, and calamus; spermatophore number; and fecundity (SM.01 Table S2).

Pre-mature males of *M. aegir* are found in the Barents and Kara Seas, and both individuals have five spermatophores. Mature males found in the same areas have 7–22 spermatophores. There is an ontogenetic increase in the size of normal spermatophores in *M. aegir*: of six males with spermatophores in both the spermatophoric sac and terminal organ, older spermatophores (in the terminal organ) were smaller than younger spermatophores (in the spermatophoric sac) in five males, while in one male they were of similar size. Thus, the ontogenetic increase in spermatophore length is 0.0–35.2% (18.1% ± 6.3%). Spermatophore width increases by 0.0–25.0% (12.4% ± 5.6%). Among spermatophore parts, the most significant ontogenetic increase is shown by the head (71.4% ± 27.2%), and for other parts the increase is less pronounced: ejaculatory apparatus (19.5% ± 10.0%), cement body (14.6% ± 6.2%), and seminal reservoir (13.6% ± 7.3%). The posterior cavity does not show ontogenetic size changes. The seminal reservoir width and volume show a larger ontogenetic increase than its length, 14.0% ± 6.4% and 48.9% ± 18.6%, respectively.

Tentative spermatophores were found in one early maturing male off Iceland, one late-maturing male, and two mature males from the Barents Sea, in addition to which a spermatophore-like structure was found in one of the mature males. Tentative spermatophores are 25–50% shorter than normal spermatophores, and have a relatively longer cement body and a relatively shorter ejaculatory apparatus and seminal reservoir (Fig. 5f). The latter is semi-translucent, seemingly containing a lower sperm concentration. The spermatophore-like structure is represented by a heavily coiled and largely empty tube (Fig. 5g).

Sperm is present in the oviducal glands of mature, pre-spent and spent females, and is not found in females at the late maturity or earlier. Oogenesis starts synchronously, but two separate portions of different-sized oocytes are clearly visible throughout the ovary from the early maturing stage: one portion remains at the pre-vitellogenic stage with no further development (Fig. 6h), while the other portion is already at least at the small vitellogenic stage and continues development (Table 4). Oocytes from both portions occasionally undergo resorption from the late maturing stage of females (0.6–28% of fecundity), and all remaining oocytes are resorbing in pre-spent and spent females, except for post-ovulatory follicles (Fig. 6e, f) and residual ripe oocytes, if any of the latter remain in the ovary (Table 4). Two residual ripe oocytes in capsules (thus, obviously fertilized) in the ovary of pre-spent female represent an abnormal state, as they should be in the distal oviduct(s). The realized fecundity of the species is 10.8–54.1% (44.7% ± 6.2%). A Barents Sea spent female (BS-322–2014) of ML 20 mm has a fecundity of 65 oocytes, which is the lowest recorded, and it is constituted by seven post-ovulatory follicles and 58 resorbing oocytes with a diameter less than 1 mm, indicating that this female realized less than 11% of its fecundity. If this female is excluded as an abnormal individual, the realized fecundity of the species is 39.4–58.1% (50.4% ± 3.0%).

The equations to estimate ML and body mass of *M. aegir* from upper and lower beak hood lengths are provided in Table 5.

**Table 5:** Equations to estimate mantle length and body mass from upper and lower hood length of the beak in *Muusoctopus aegir* Golikov, Gudmundsson & Sabirov, **sp. nov.** and *M. johnsonianus* (Allcock, Strugnell, Ruggiero & Collins, 2006) [26]

Species/measurement		*Muusoctopus aegir* sp. nov.	*Muusoctopus johnsonianus*
**UHL**	**ML**	ML = 1.92UHL^1.67^ *n* = 28, *r*^2^ = 0.82, ***p* < 0.0001**	ML = 2.61UHL^1.49^ *n* = 6, *r*^2^ = 0.82, ***p* = 0.0179**
	**BM**	BM = 0.03UHL^4.35^ *n* = 28, *r*^2^ = 0.79, ***p* < 0.0001**	BM = 0.03UHL^4.28^ *n* = 6, *r*^2^ = 0.18, *p* = 0.41
**LHL**	**ML**	ML = 7.37LHL^0.97^ *n* = 28, *r*^2^ = 0.63, ***p* < 0.0001**	ML = 3.57LHL^1.61^ *n* = 6, *r*^2^ = 0.92, ***p* = 0.0243**
	**BM**	ML = 0.91UHL^2.57^ *n* = 28, *r*^2^ = 0.63, ***p* < 0.0001**	ML = 0.07UHL^4.67^ *n* = 6, *r*^2^ = 0.93, ***p* = 0.0013**

Significant *p*-values are in bold. *n*, number of individuals; *r*^2^, determination coefficient

*ML* mantle length, *BM* body mass, *UHL* upper beak hood length, *LHL* lower beak hood length

##### Remarks

Muus [23] recognized three species of *Bathypolypus* (*B. arcticus*, *B. bairdii* and *B. pugniger*) from the Arctic Atlantic. These three species differ from *Muusoctopus aegir* in being larger, and in having: 1) different funnel organs (V V- or II II-shaped); 2) proportionally shorter arms with fewer and proportionally smaller suckers (including on the hectocotylized arm); 3) proportionally larger and more prominent eyes; 4) fewer gill lamellae; 5) stylets; 6) different female fecundity; 7) papillose skin and supraocular cirri, with paler ventral mantle pigmentation; 8) different radula characteristics, especially rachidian dentition; 9) ligula morphology; and 10) spermatophore number and morphology [23, 90]. Likewise, *M. aegir* fits the amended diagnosis of *Benthoctopus* (widely recognized as *Muusoctopus* now [31, 32]) from Strugnell et al. [56] in having: 1) a fitting relative arm length; 2) a crop diverticulum and a fitting radula; and 3) a ligula with small indistinct rugae.

The holotype of *Be. piscatorum* has twice been stated to be a synonym of *B. bairdii* [23, 26]. Using the photos of the holotype provided by I. G. Gleadall and in O’Shea [77] and its measurements [30, 77, 87], we again demonstrate it is indeed rather a *Bathypolypus*: it fits *Bathypolypus* in mantle and head proportions, and having large prominent eyes; proportional length of arms and sucker count are lower than in *Muusoctopus*, and suckers are proportionally smaller than in all Arctic and North Atlantic *Muusoctopus*, except for *M. normani*; funnel length and free funnel length are shorter than in all Arctic and North Atlantic *Muusoctopus*, except for *M. normani*; it lacks a crop diverticulum, which is present in Arctic and North Atlantic *Muusoctopus*; and coloration fits *Bathypolypus*. Muus [23] described remains of an eye cirrus (a character of *Bathypolypus*) over the right eye in this individual; and this individual has stylets [A. L. Allcock, pers. comm.]. Unfortunately, Verrill [30, 87] did not provide any measurements of the other *Be. piscatorum* female he described from Northwest Atlantic. The male of *Be. piscatorum* from Verrill [88, 89] also does not have any measurements provided, and its hectocotylus, as estimated from Verrill’s drawing [89] [Pl. XLII, Fig. 5], is small and resembles that of *M. normani*. Another individual referred to as *Be. piscatorum* even after the suggested synonymy [23] is a large female from Placentia Bay [48, 53, 54]. Its large size, mantle and head proportions, proportional web depth and sucker diameter, gill lamellae count, and ripe oocyte size and count fit those of *M. normani*; however, its arms are proportionally shorter, and sucker count, funnel measurements, and funnel organ morphology are not reported.

Individuals from the Faroe–Shetland Channel [34, 41–43] are of globular shape, which is the case for *M. aegir* described here, and are very different from the individuals of Verrill [30, 87–89] and those from Placentia Bay [48, 53, 54]. Moreover, these individuals from the Faroe–Shetland Channel have their mantle and body proportions, hectocotylized arm sucker count, sucker diameter, free funnel length, and gill lamellae count fitting *M. aegir*, and also have a crop diverticulum [34, 41–43]. Funnel organ morphology, W-shaped with medial linb longer, according to Robson [43], can be seen as fitting those of *M. aegir*: in *M. aegir*, medial limbs are also sometimes longer than marginal limbs (but generally are of similar length). The radula illustrated by Robson [43] [Fig. 35] is typical for *M. aegir*, with a rachidian with seven cusps—a character state not reported for other North Atlantic and Arctic species of *Muusoctopus* [26, 63] [this study, *M. johnsonianus* below]. Ligula length from Hoyle [34], reported in Massy [38], and from Robson [43] fit *M. aegir*. Ligula width and ligula rugae count from Russell [41, 42] fit those of *M. aegir*. Ligula length from Russell [41, 42] is shorter, and ligula width from Hoyle [34], reported in Massy [38], is narrower than in *M. aegir*. Small sizes and proportionally longer arms in Russell [41, 42] fit immature *M. aegir* (SM.01 Table S1), which can explain their shorter ligula. Overall, the individuals from the Faroe–Shetland Channel [34, 41–43] are very different from Verrill’s [30, 87–89] *Be. piscatorum* but conform fully to the morphology of *M. aegir*. Finally, ‘*Muusoctopus* sp.’ was recently reported from the Faroe–Shetland Channel and that it did not coincide with any known species (with *COI* barcode, but no morphological description of the species provided) [57]. The locations are very close to the old individuals reported from the area [34, 41–43] (SM.01 Fig. S2). The depths of older (908–1112 m [34, 41–43]) and newer (704–1198 m [57]) records coincide, and are close to mean depth of our Iceland records of *M. aegir* (418–2442, 950.9 ± 173.4 m). Based on the morphological similarity of the old Faroe–Shetland Channel individuals [34, 41–43] and our individuals, and similar depths and location of the older [34, 41–43] and newer [57] Faroe–Shetland Channel individuals, we suppose the latter are also *M. aegir*. As such, *COI* barcode of ‘*Muusoctopus* sp.’ from the Faroe–Shetland Channel reported in Taite et al*.* [57] is applicable to *M. aegir* described here. Biogeography also supports this view particularly well for the Icelandic and Faroese waters: 1) during the extensive sampling around Iceland (579 bottom stations) during BIOICE program, *M. aegir* was only recorded to the north of the Greenland–Iceland–Faroe Ridges. In the extensive sampling reported by Taite et al*.* [57], ‘*Muusoctopus* sp.’ was only found in the Faroe–Shetland Channel, where individuals exhibiting characters fitting those of *M. aegir* were recorded previously [34, 41–43]; and 2) only *M. johnsonianus* was found in the BIOICE samples south of the Greenland–Iceland–Faroe Ridges, but *M. aegir* was absent. In Taite et al*.* [57], *M. johnsonianus* and *M. normani* were found to the south of the Faroe–Shetland Channel, but never ‘*Muusoctopus* sp.’ (SM.01 Fig. S2). It is clear that the Arctic species, *M. aegir*, is separated from the North Atlantic species, *M. johnsonianus* and *M. normani*, by the Canada–Greenland and Greenland–Iceland–Faroe Ridges.

Other historical records of *Be. piscatorum* are discussed below. There is an individual from Massy [37], caught near Ireland and initially described as *Polypus normani* Massy, 1907 [37], but later reconsidered to be *Be. piscatorum* [38, 39]. Measurements of this individual fit *M. normani*, which is shown by Allcock et al*.* [26], who reinstalled this species as *M. normani*. Regarding the Arctic records of *Be. piscatorum*: in Appelløf [36], one individual off Ranen fits *M. aegir* and individuals off Isfjord fit *Bathypolypus*; in Grieg [44], all Svalbard individuals fit *M. aegir*, except for the large female from North Atlantic, which is rather one of the Atlantic species of *Muusoctopus*; and high Arctic individuals from Nesis [51] fit *M. aegir* so well, that they were used in species description here, to complement our samples. To summarize, studies that reported *Be. piscatorum* in the North Atlantic and Arctic [22, 34–54] were in fact mixing Verrill’s *Bathypolypus*, two Atlantic species of *Muusoctopus* (*M. johnsonianus* and *M. normani*), and Arctic *M. aegir*, which reaches the Faroe–Shetland Channel as a southernmost part of its range. This means that the published records of *Be. piscatorum* in the Faroe–Shetland Channel, along the northern slope of the Greenland–Iceland–Faroe Ridge, farther to the east along the Norwegian slope and the continental slopes of the Barents and Kara Seas [34–36, 40–47, 49–52] are in fact *M. aegir*.

No other known *Muusoctopus* species occur within the recognized distribution of *M. aegir*. *Muusoctopus aegir* differs from both *M. johnsonianus* and *M. normani* most notably in: 1) being smaller and in having a more rounded mantle; 2) having relatively more of the funnel free from the ventral surface of head than in *M. normani*, and less than in *M. johnsonianus*; 3) having proportionally shorter arms with fewer, larger suckers (including hectocotylized arms), and fewer gill lamellae than *M. johnsonianus* (but largely overlapping with *M. normani*); 4) lacking stylets; 5) having a rachidian with 5–7 cusps, with differing seriation (vs. five cusps in the other two species); 6) and differing in coloration (Table 9) [26, 32] [this study, section on *M. johnsonianus* below]. Proportionally, the ligula of *M. aegir* is longer than that in either of the other species, and broader than that in *M. johnsonianus* (no data are available for *M. normani*); the calamus is shorter than that in *M. normani*, but similar to that of *M. johnsonianus*; the spermatophores are shorter than that in *M. normani* (insufficient data on *M. johnsonianus* to compare); and females have fewer and relatively larger ripe oocytes (large vitellogenic oocytes used as a proxy for *M. johnsonianus*) (Table 9) [26, 32, 90] [this study, section on *M. johnsonianus* below]. The funnel organ enables unambiguous differentiation of these species; in *M. normani* it is V V-shaped; and while it is similarly W-shaped in *M. johnsonianus*, with the marginal limbs of comparable length or slightly longer than the medial limbs, in *M. aegir* the medial limbs are usually slightly longer than the marginal, and the marginal limbs are broader than in *M. johnsonianus* (Figs. 3a–d, 11; Table 9) [26, 32] [this study, section on *M. johnsonianus*, below].

For differences from *Muusoctopus* sp. 1 from the northern Baffin Bay and Canadian Arctic Archipelago and *M. sibiricus* from the Siberian, Chukchi and Beaufort Seas, see Remarks sections for each species, below. *Muusoctopus leioderma* from the Chukchi Sea, adjacent to the Bering Strait 1) seems to be larger and to have a narrower mantle than *M. aegir*; 2) has a W-shaped funnel organ with medial limbs always longer than marginal limbs; 3) has stylets; 4) has more gill lamellae; 5) has a proportionally longer ligula and shorter calamus; 6) has different coloration; and 7) has a lateral skin fold and small papillae (Table 9) [65–67, 92].

#### *Muusoctopus* sp. 1

**Fig. 8:**
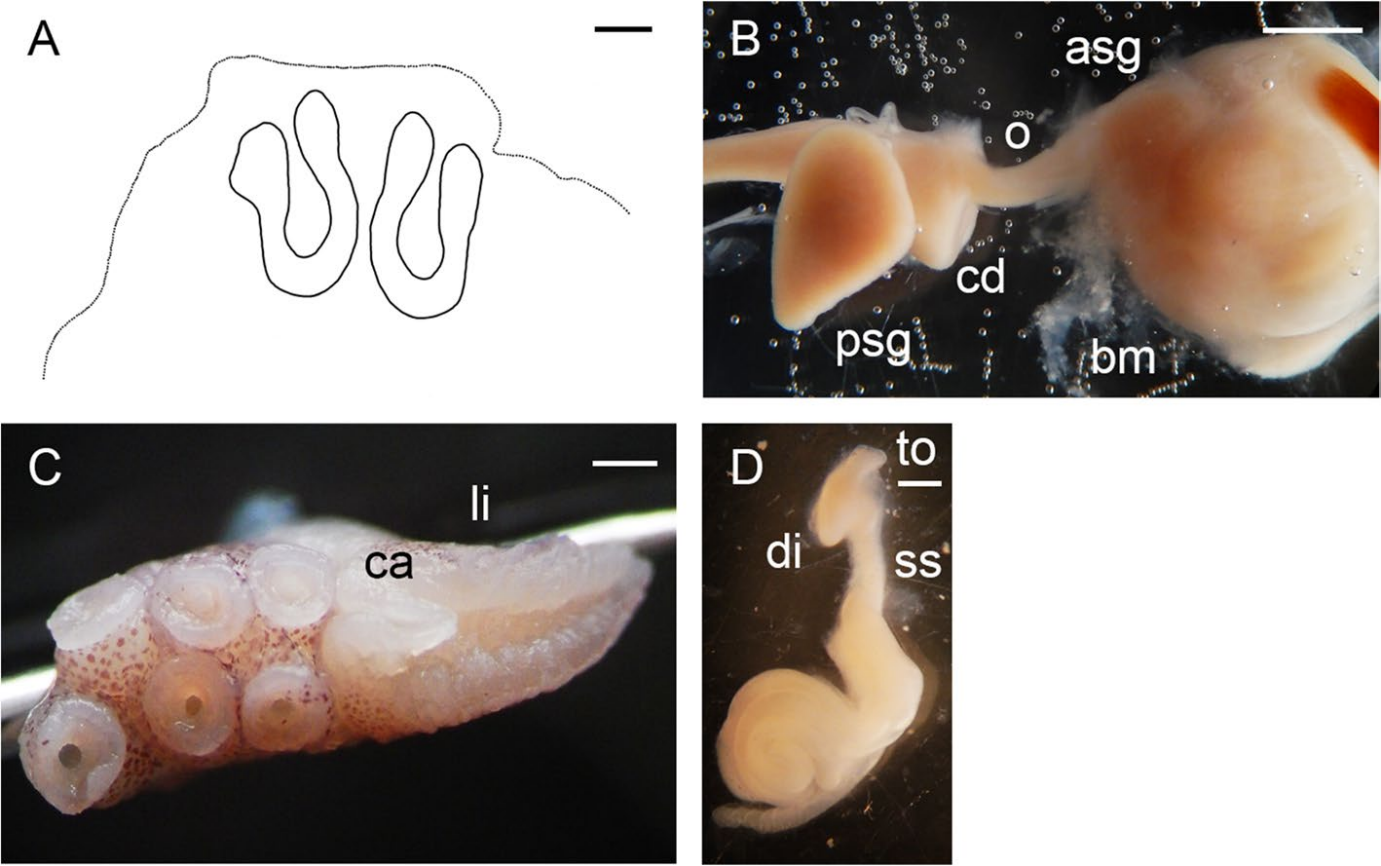
*Muusoctopus* sp. 1. General anatomy, hectocotylus and male reproductive anatomy. **a**, **d**, GRL-PA-7–20-2–2016 (early immature male, mantle length (ML) 23 mm, northern Baffin Bay): funnel organ (**a**) and spermatophoric complex (**d**), in situ, ventral view; **b**, **c**, GRL-PA-7–20-1–2016 (late immature male, ML 30 mm, northern Baffin Bay): anterior part of digestive tract (**b**) and hectocotylus (**c**). Scale bars: **a**, **c**, **d** = 1 mm, **b** = 5 mm. Abbreviations: asg, anterior salivary gland; bm, buccal mass; ca, calamus; cd, crop diverticulum; di, diverticulum; e, esophagus; li, ligula; psg, posterior salivary gland; ss, spermatophoric sac; to, terminal organ

**Fig. 9:**
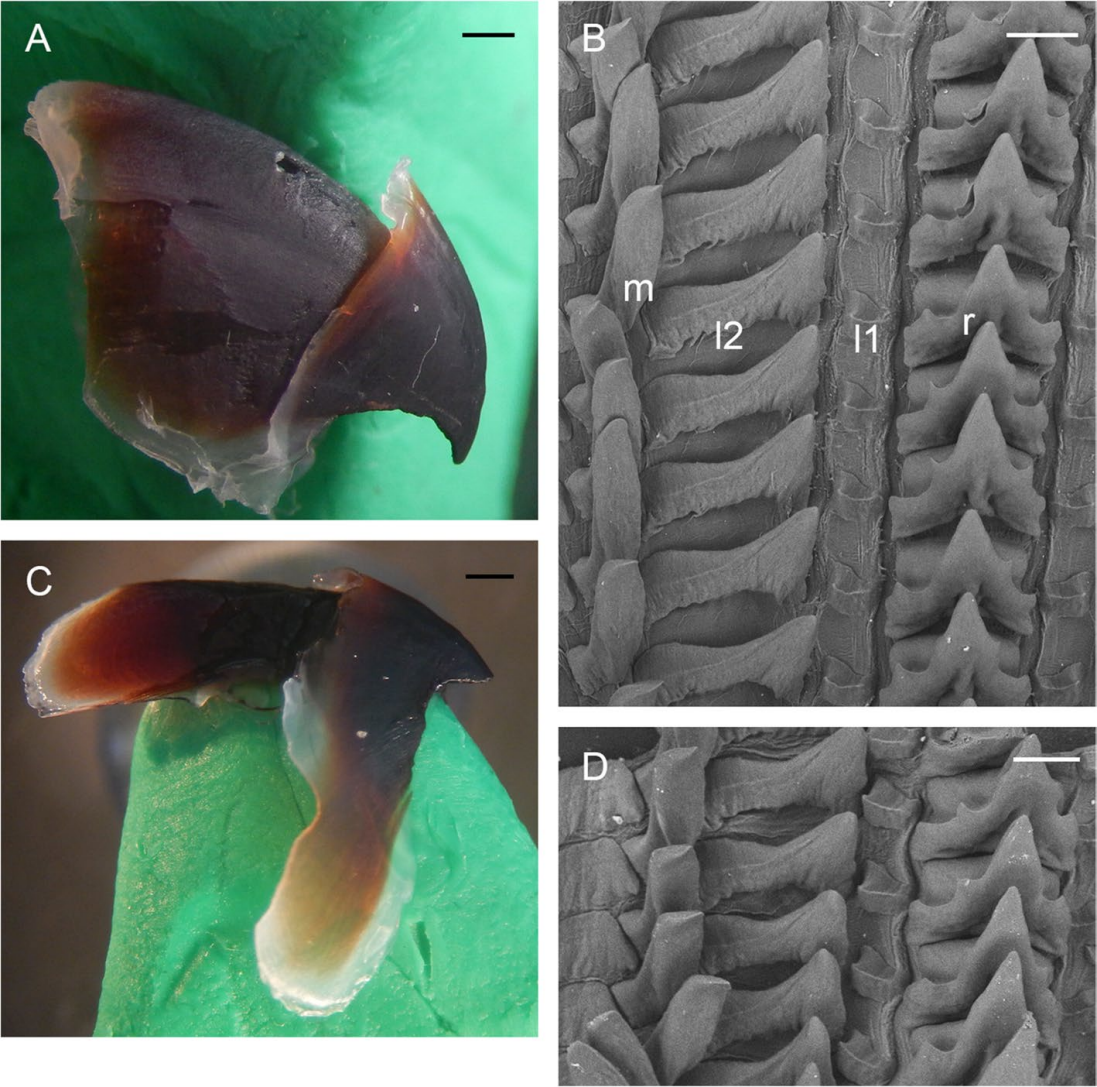
*Muusoctopus* sp. 1. Beak and radula. GRL-PA-7–20-1–2016 (late immature male, mantle length 30 mm, northern Baffin Bay): upper (**a**) and lower (**c**) beak, and unworn sections of radula (**b**, **d**). Scale bars: **a**, **c** = 1 mm, **b**, **d** = 100 µm. Abbreviations: l1, first lateral tooth; l2, second lateral tooth; m, marginal tooth; r, rachidian tooth

(Table 6; Figs. 7, 8 and 9).

**Table 6:** Data on individuals of *Muusoctopus* sp. 1

Individuals/ character	GRL-PA-7- 20–1-2016	GRL-PA-7- 20–2-2016	GRL-PA-7- 117–2016	USNM 574859^a^
Area	GRL	GRL	GRL	CAN
Sex	Male	Male	Female	Female
Maturity stage	Late immature (II)	Early immature (I)	Early immature (I)	Late immature (II) (?)
ML, mm	30	23	22	31
TL, mm	148	118	99	n/a
Ventral ML, mm	26	19	17	n/a
Mantle width, mm	32	20	23	34
Head length, mm	13	9	8	n/a
Head width, mm	26	19	17	n/a
Eye diameter, mm	8.5	7.0	6.0	n/a
Lens diameter, mm	3.0	2.0	2.0	n/a
Funnel length, mm	13.0	9.0	9.0	14.0
Free funnel length, mm	7.0	5.5	6.0	11.0
Web depth, mm (min – max)	20–27	12–16	13–17	13–21
Web formula	a > c > b > d > e	b > c > d > a > e	b > c = d > e > a	c = d > b > a > e
Arm length, mm (min – max)	79–105	69–86	54–69	80–85
Arm formula	1 > 2 = 4 > 3	1 > 2 > 3 > 4	1 > 2 > 3 > 4	3 > 1 = 2 = 4
Sucker count (min – max)	102–118	88–108	84–100	81
Sucker diameter (max), mm	3.0	2.0	2.0	3.0
Gill length, mm	13.5	9.6	8.6	n/a
Gill lamellae count, outer/inner	10/9	10/9	9/9	8/8
Hectocotylized arm length, mm	74	59	–	–
Hectocotylized arm sucker count	66	56	–	–
Ligula length, mm	4.8	2.7	–	–
Ligula width, mm	3.0	1.5	–	–
Calamus length, mm	1.8	1.8	–	–

*ML* mantle length, *TL* total length, *GRL* north Baffin Bay (West Greenland), *CAN* Canadian Arctic Archipelago; *n/a* not analyzed

^a^examined by A. L. Allcock (A. L. Allcock, unpubl. data)

##### Material examined

Baffin Bay (individuals no longer extant): ♂II, ML 30 mm, GRL-PA-7–20-1–2016 and ♂I, ML 23 mm, GRL-PA-7–20-2–2016, Stn 20, 74.11°N, 57.94°W, 450.5 m, BT 2.00 °C, 23 September 2016; ♀I, ML 22 mm, GRL-7–117-2016, Stn 117, 73.72°N, 58.39°W, 393 m, BT 1.97 °C, 30 September 2016.

Canadian Arctic Archipelago (USNM 574859, examined by Dr. A. Louise Allcock, with data provided to A.V.G.): ♀II, ML 31 mm, USNM 574859, 74.72°N, 94.70°W, 101 m, 8 August 1950.

##### Description

Counts and measurements for the species are given in Table 6, and indices are given in Table 9.

The following description is based on all individuals studied. Species small, ML 22–31 (26.5 ± 2.3) mm, TL 99–148 mm (121.7 ± 14.3 mm) mm; ventral ML 5 mm shorter than dorsal ML in Baffin Bay individuals. Mantle width and length similar (102.0% ± 5.1% ML); head width 79.5% ± 5.9% mantle width (Fig. 7). Eyes relatively prominent, of diameter 28.7% ± 0.9% ML (Fig. 7). Funnel of moderate length 42.1% ± 1.3% ML, tapered, free from ventral surface of head for ~ 50–80% its length. Funnel organ V V-shaped, with medial limbs slightly longer and broader than marginal limbs (Fig. 8a). Arms ~ 2.9 times ML (Fig. 7), of subequal length, and typically formula is 1.2.3.4. Suckers biserial, closely set from base of arms to arm tips, moderately sized (9.4% ± 0.3% ML). Suckers: number 81–118 (99.9 ± 5.3) per arm, none enlarged in either sex; with 81 (USNM 574859) and 84–110 (Baffin Bay individuals) on arm pair 3. Web relatively shallow, depth 20.1% ± 2.2% longest arm length;all web sectors are approximately subequal, with sectors C and B deepest, and A and E most shallow.

Gills very long (41.9% ± 1.7% ML), with 8–10 (mode: 10) outer and eight or nine (mode: 9) inner lamellae per demibranch. Presence of stylets not examined. Upper beak with hooked rostrum (Fig. 9a); lower beak with broad, straight rostrum (Fig. 9c); both typically *Muusoctopus*. Anterior salivary glands small (19.2%. ± 0.7% ML), discoid. Posterior salivary glands very large (31.4% ± 0.3% ML), approximately triangular. Crop diverticulum well developed (Fig. 8b). Presence of rectum loop not examined. Ink sac and well-developed ink duct absent (presence of vestigial ink duct not examined). Anal flaps absent. Radula with nine elements per transverse row; rachidian pentacuspid, the central largest, with asymmetrical lateral cusps with 4 or 5 seriation (Fig. 9b, d); marginal and lateral teeth unicuspid, marginal teeth curved. Marginal plates well developed (Fig. 9b, d).

Male third right arm hectocotylized (Fig. 7d), of length 246.7% and 256.5% ML (*n* = 2) and 79.7% and 93.7% the opposite arm, with 56–6 suckers. Ligula small, 4.6% and 6.5% of hectocotylized arm length, broad, 55.6% and 62.5% ligula length, tapering acutely. Ligula with distinct margins and well-marked shallow groove without transverse ridges, but with 12 or 13 low indistinct rugae; groove and margins of similar width for 2/3 of ligula length basally (Fig. 8c). Calamus very large, 37.5% or 66.7% ligula length, and pointed (Fig. 8c). Spermatophoric complex in males is translucent due to immaturity, but fully formed (Fig. 8d); not measured. Female reproductive tract not examined.

Skin smooth, without papillae (Fig. 7). Live color is violet-brown, paler ventrally, with no white area around the mouth (Fig. 7).

##### Distribution

Known from the Canadian Arctic Archipelago and northern Baffin Bay (Fig. 1), at 101–450.5 m (314.8 ± 108.2 m), with the associated bottom temperatures 1.97–2.00 °C.

##### Biology and ecology

Unknown.

##### Remarks

It is unknown whether the ranges of *M. aegir* and *Muusoctopus* sp. 1 overlap to the north of Greenland, or if the ranges of *M. sibiricus* and *Muusoctopus* sp. 1 overlap in the western marginal area of the Canadian Arctic Archipelago. *Muusoctopus* sp. 1 differs from *M. aegir* in having: 1) a slightly larger body size (immature individuals are larger than the respective maturity stages of *M. aegir*); 2) a relatively longer free funnel length; 3) funnel organ shape (V V- vs. W- in *M. aegir*); 4) larger sucker counts (sucker counts on immature individuals exceed those of mature *M. aegir*); 5) relatively longer gills with more gill lamellae (the values are overlapping though); 6) a pentacuspid rachidian with reduced seriation (4–5 vs. 4–6 in *M. aegir*); and 7) a ligula of different shape and size, judging by morphology of immature individuals of *Muusoctopus* sp. 1 (Table 9). Coloration also differs slightly: *Muusoctopus* sp. 1 is slightly darker than *M. aegir* and lacks a whitish area around the mouth (Figs. 2, 7).

*Muusoctopus* sp. 1 is differentiated from *M. sibiricus* in the remarks section for *M. sibiricus*, below. While limited data exist for both *Muusoctopus* sp. 1 and *M. leioderma* (see [65–67, 92] for *M. leioderma*), they differ in: 1) funnel organ shape (V V- vs. W- in *M. leioderma*); 2) *M. leioderma* has relatively shorter gills with more gill lamellae (non-overlapping values); 3) size and shape differences in ligula present, as can be judged from immature *Muusoctopus* sp. 1; and 4) *M. leioderma* has a different coloration, and a lateral skin fold and small papillae (Table 9).

North Atlantic species (*M. johnsonianus* and *M. normani*) differ from *Muusoctopus* sp. 1 in being larger, and in having a proportionally narrower mantle. *Muusoctopus* sp. 1 also differs from *M. normani* in having a proportionally shorter portion of free funnel, from *M. johnsonianus* in funnel organ shape, and from both *M. johnsonianus* and *M. normani* in having relatively shorter arms with fewer and larger suckers (including hectocotylized arms), and relatively longer gills (with more lamellae than *M. normani*, although values overlap); and size and shape differences in ligula present, as can be judged from immature *Muusoctopus* sp. 1 (Table 9) [26, 32] [the following section on *M. johnsonianus*, below].

#### *Muusoctopus johnsonianus* (Allcock, Strugnell, Ruggiero & Collins, 2006) [26]

**Fig. 10:**
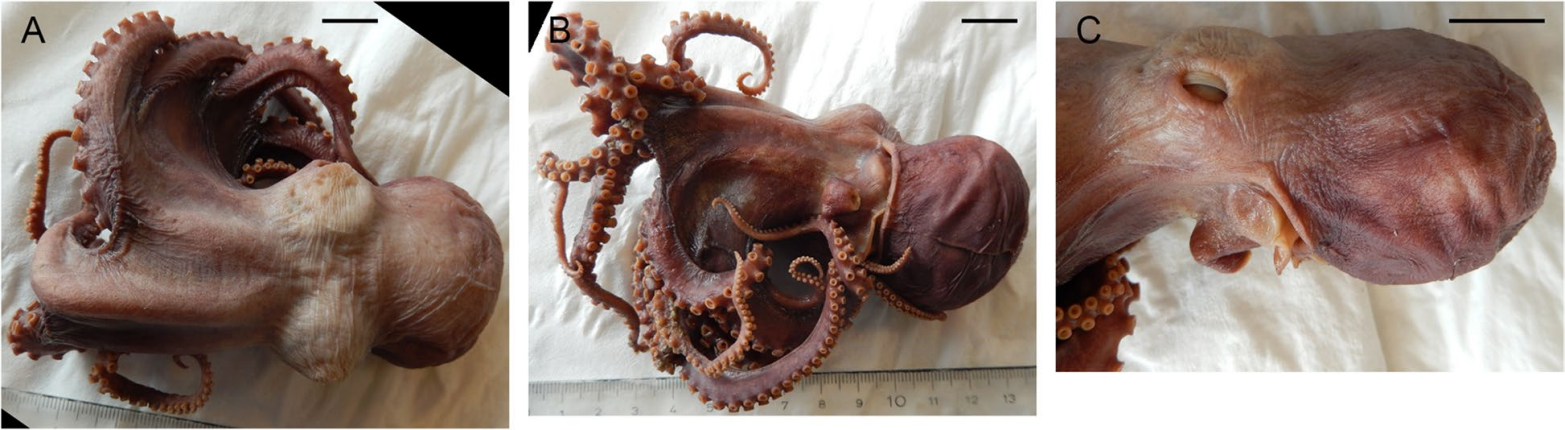
*Muusoctopus johnsonianus* (Allcock, Strugnell, Ruggiero & Collins, 2006) [26]. External view. BIOICE-3520–1 (late immature female, mantle length 41 mm, off Iceland, fixed): dorsal (**a**), ventral (**b**) and lateral (**c**) view. Scale bars = 10 mm

**Fig. 11:**
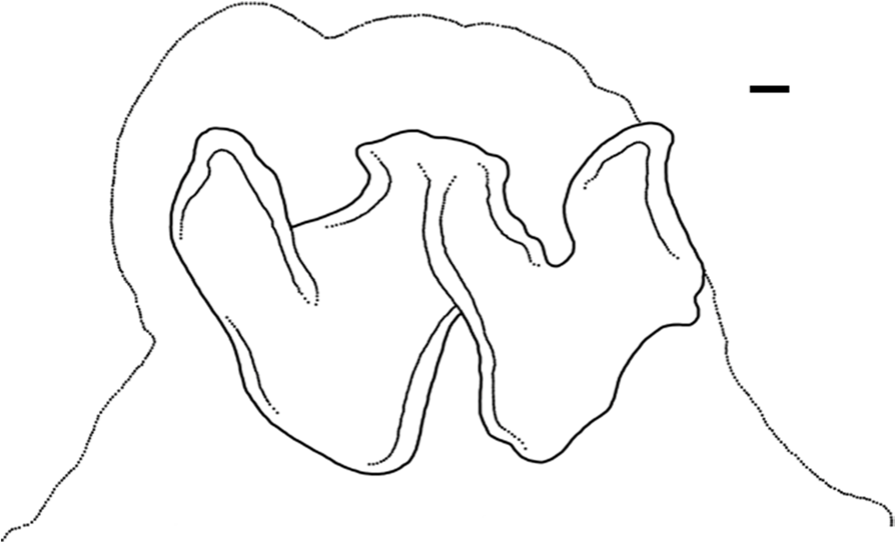
*Muusoctopus johnsonianus* (Allcock, Strugnell, Ruggiero & Collins, 2006) [26]. Funnel organ. BIOICE-3168 (early maturing male, mantle length 47 mm, off Iceland). Scale bar = 1 mm

**Fig. 12:**
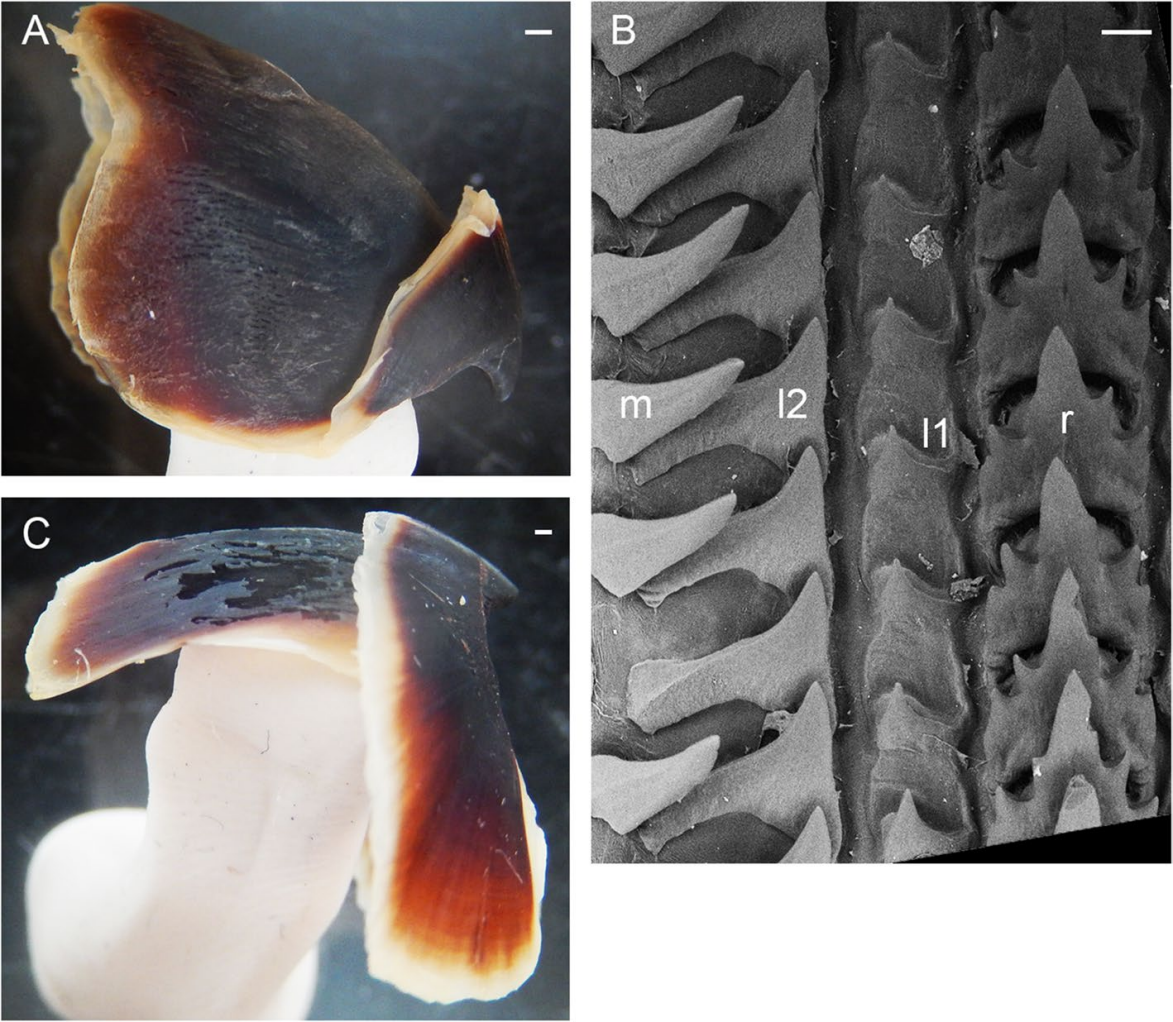
*Muusoctopus johnsonianus* (Allcock, Strugnell, Ruggiero & Collins, 2006) [26]. Beak and radula. BIOICE -3520–2 (late maturing female, mantle length 89 mm, off Iceland): upper (**a**) and lower (**c**) beak, and unworn section of radula (**b**). Scale bars: **a**, **c** = 1 mm, **b** = 100 µm. Abbreviations: l1, first lateral tooth; l2, second lateral tooth; m, marginal tooth; r, rachidian tooth

**Fig. 13:**
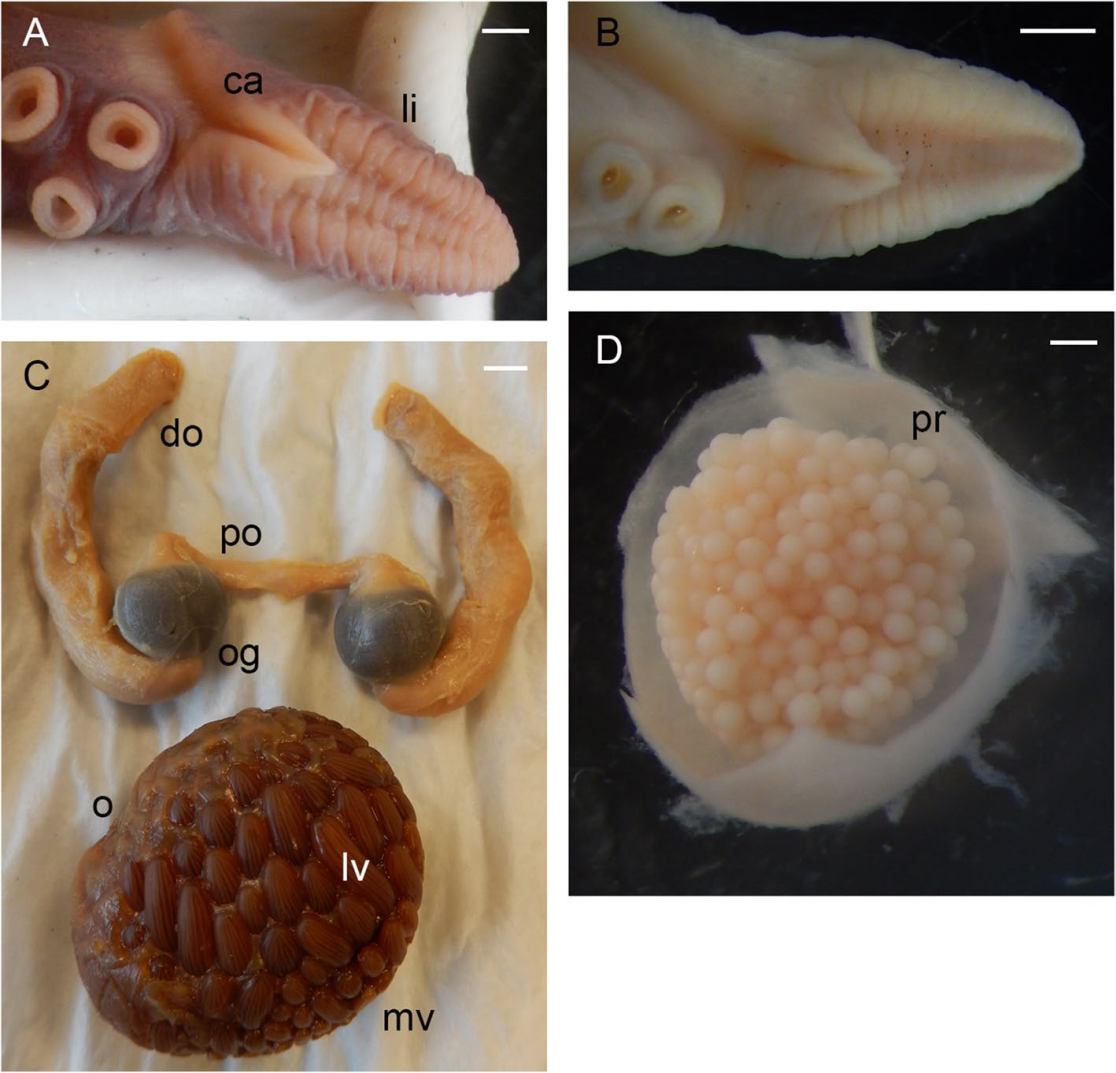
*Muusoctopus johnsonianus* (Allcock, Strugnell, Ruggiero & Collins, 2006) [26]. Hectocotylus and female reproductive anatomy. **a**, BIOICE-3524 (early maturing male, mantle length (ML) 49 mm, off Iceland): hectocotylus; **b**, BIOICE-3168 (early maturing male, ML 47 mm, off Iceland): hectocotylus; **c**, BIOICE -3520–2 (late maturing female, ML 89 mm, off Iceland): dissected female reproductive tract; **d**, BIOICE-3521 (late immature female, ML 36 mm, off Iceland): dissected ovary. Scale bars: **a**, **b** = 1 mm, **c** = 5 mm, **d** = 0.5 mm. Abbreviations: ca, calamus; do, distal oviduct; li, ligula; lv, late vitellogenic oocyte; mv, mid-vitellogenic oocyte; o, ovary; og, oviducal gland; po, proximal oviduct; pr, pre-vitellogenic oocyte

(Tables 4, 5, 7; SM.01 Table S3; Figs. 10, 11, 12 and 13).

**Table 7:** Data on maturing individuals of *Muusoctopus johnsonianus* (Allcock, Strugnell, Ruggiero & Collins, 2006) [26]. Immature individuals are detailed in SM.01 Table S3. All individuals are from Iceland

Individuals/character	BIOICE- 3524	BIOICE- 3168	BIOICE- 3520–1
Sex	Male	Male	Female
Maturity stage	Early maturing (III)	Early maturing (III)	Late maturing (IV)
ML, mm	49	47	89
TL, mm	256	255	425
Ventral ML, mm	41	40	66
Mantle width, mm	49	46	74
Head length, mm	29	29	33
Head width, mm	52	43	58
Eye diameter, mm	27.0	25.0	29.0
Lens diameter, mm	8.0	7.0	9.0
Funnel length, mm	23.0	26.0	39.0
Free funnel length, mm	14.0	16.0	28.0
Web depth, mm (min – max)	42–53	46–58	56 –85
Web formula	b > c = a > d > e	a = d > b > c > e	b > c > a > d > e
Arm length, mm (min – max)	154–178	148–179	242–303
Arm formula	2 > 1 > 3 > 4	2 > 1 > 3 > 4	1 > 2 > 3 > 4
Sucker count (min – max)	128–136	130–136	146–164
Sucker diameter (max), mm	4.0	4.0	8.0
Gill length, mm	14.5	15.0	28.0
Gill lamellae count, outer/inner	10/9	10/9	10/9
Hectocotylized arm length, mm	137	129	–
Hectocotylized arm sucker count	68	68	–
Ligula length, mm	7.0	5.0	–
Ligula width, mm	4.0	2.4	–
Calamus length, mm	2.0	2.0	–
Fecundity	–	–	227

*ML* mantle length, *TL* total length

##### Synonymy

*Benthoctopus johnsoniana* Allcock, Strugnell, Ruggiero & Collins 2006 [26]: 379, Figs. 6–9.

*Muusoctopus johnsonianus* (Allcock, Strugnell, Ruggiero & Collins, 2006) [26] – Gleadall, 2013 [32]: 113, fig. 3.

*Benthoctopus* sp. – Collins et al*.* 2001 [22]: 112; Barrat et al*.* 2007 [90]: 392.

##### Material examined

Iceland: IINH 37,816, ♂III, ML 49 mm, BIOICE Stn 3524, 62.64°N, 17.05°W, 1919.5 m, BT 2.37 °C, 7 September 2002; IINH 37,815, ♂III, ML 47 mm, BIOICE Stn 3168, 60.92°N, 22.78°W, 1899.5 m, BT 2.98 °C, 26 July 2000; IINH 37,829, ♂I, ML 8.5 mm, BIOICE Stn 2427, 63.16°N, 20.06°W, 778 m, BT 5.50 °C, 3 July 1993; IINH 38,040, ♀IV, ML 89 mm, BIOICE Stn 3520, 62.26°N, 17.54°W, 1957 m, BT 2.70 °C, 5 September 2002; IINH 37,817, ♀II, ML 49 mm, BIOICE Stn 3520, 62.26°N, 17.54°W, 1957 m, BT 2.70 °C, 5 September 2002; IINH 37,818, ♀II, ML 36 mm, BIOICE Stn 3521, 62.52°N, 17.17°W, 1937.5 m, BT 2.34 °C, 7 September 2002; IINH 37,826, ♀I, ML 13 mm, BIOICE Stn 2926, 65.86°N, 28.78°W, 540 m, 27 August 1996.

##### Additional material examined

See SM.01.

##### Description

Counts and measurements for the species are given in Table 7 and SM.01 Table S3, and indices are given in Table 9.

Description based on individuals of maturity stages III and IV (two males and one female), and reports only ‘what-is-new’ in relation to [26, 32]; reference to ‘combined data’ includes values from [26, 32]. Mantle from wide oval to rounded, of width to 100.0% ML. Head width occasionally exceeds mantle width, to 106.1% mantle width, mean 88.4% ± 4.8% mantle width (combined data). Eyes very prominent, their diameter 47.0% ± 7.2% ML (Fig. 10). Funnel organ W-shaped, with medial and marginal limbs of similar length, or with moderately broad marginal limbs slightly longer (Fig. 11). Arms relatively long; in our individuals ~ 350% ML compared with 400% ML in combined data, with arm formula typically 2.1.3.4 (combined data). Arms with 128–164 (140.3 ± 3.3) suckers. Web medium deep (combined data, 25.1% ± 1.3% longest arm length), with sectors B and C deepest, and A and E most shallow.

New material has 10 lamellae per outer and 9 lamellae per inner demibranch; in combined data, it is 8–11 (mode: 10) outer and 8–10 (mode: 9) inner lamellae per demibranch. Rostra of both upper and lower beaks broad, and upper one hooked (Fig. 12a, c). Rachidian pentacuspid, with large central cusp and smaller asymmetrical lateral cusps with 4 or 5 seriation (Fig. 12b).

Hectocotylized arm relatively long, 268.6% ± 12.9% ML, and 78.5% ± 4.2% opposite arm (combined data), with 68 suckers in new material (67–71 in combined data). Ligula of medium size, 3.9–10.6% (6.3% ± 1.0%) hectocotylized arm length (combined data), broad, 48.0% and 57.1% ligula length (*n* = 2), tapering gradually. Ligula with distinct margins and well-marked shallow groove without transverse ridges, but with 15 and 18 low indistinct rugae (*n* = 2); with groove and margins of the same width for a half of ligula length basally (Fig. 13a, b). Calamus large, 28.0–43.5% (36.9% ± 2.8%) ligula length (combined data), and pointed (Fig. 13a, b). Length of terminal organ with diverticulum 17.0% and 22.4% ML (*n* = 2). Spermatophores absent in both studied early maturing males. Oviducal glands dark, broader than long, large (length 12.4% ML and width 15.7% ML in late maturing female) (Fig. 13c). Fecundity 227–300 (259.0 ± 21.5) oocytes (Tables 4, 6; SM.01 Table S3). Large vitellogenic oocytes range 17.0–22.0 mm with 18–20 follicular folds (Fig. 13c; Table 4).

Skin smooth (Fig. 10). Animals reverse-countershaded when alive, paler dorsally, darker (violet-brown) ventrally (Fig. 10) [26]; two of seven fixed individuals uniformly violet-brown over all body surfaces.

##### Distribution

North Atlantic slope, 15–66°N, reaching the Canada–Greenland and Greenland–Iceland–Faroe Ridges [22, 24–26, 32, 55–57] [M. Vecchione, pers. comm.; this study]. While the northernmost record is Davis Strait (65.86°N) from this study, this species may reach the Greenland–Scotland Ridge, as other deep-sea cephalopods do in the area [86, 93]. This species is reported from 797–2540 m, but the associated bottom temperatures not reported [22, 25, 26, 55–57]; we report this species from 540–1957 m (1470.6 m ± 230.4 m) and 2.34–5.50 °C (3.18 ± 0.48 °C).

##### Biology and ecology

The most mature of the studied females is at the late maturing stage. It has no sperm in the oviducal glands. In late immature females, all oocytes are pre-vitellogenic (Fig. 13d). Late maturing female has similar proportions of small vitellogenic (47% of fecundity) and medium and large vitellogenic (53% of fecundity) oocytes (Table 4). No evidence for oocyte resorption is found.

The equations to estimate ML and body mass of *M. johnsonianus* from upper and lower beak hood length are provided in Table 5.

##### Remarks

Presence of vestigial ink duct, stated for *M. johnsonianus* in [32], but not in another study of the species [26], has not been examined in IINH individuals.

The distribution ranges of *M. johnsonianus* and *M. normani* are largely similar [22, 26]. *Muusoctopus normani* differs from *M. johnsonianus* in having: 1) a relatively narrower mantle and head; 2) a shorter funnel and free part of the funnel; 3) longer arms with more and smaller suckers; 4) a longer ligula and calamus; 5) V V-shaped funnel organ (as opposed to W-shaped); and 6) in coloration, being uniformly violet-brown, whereas *M. johnsonianus* is reverse countershaded (Table 9) [26, 32] [this study]. Suckers in *M. normani* being more widely spaced than in *M. johnsonianus* is reported as a diagnostic character in [26], but dismissed in [32]; we lack *M. normani* in our samples to examine this character. Additionally, *M. normani* may have relatively longer spermatophores and larger ripe oocytes, and lower female fecundity (Table 9) [26, 32, 90] [this study]; more studies needed to analyse these characters. The indistinct rugae on ligula are ignored by [26, 32] in both species, presumably with the assumption they are simply artifacts of the preservation process.

*Muusoctopus johnsonianus* is differentiated from *M. aegir*, *Muusoctopus* sp. 1 and *M. sibiricus* in ‘Remarks’ sections for these species. From *M. leioderma* (see [65–67, 92] for *M. leioderma*), *M. johnsonianus* differs in being larger and in having: 1) a broader mantle; 2) relatively longer arms with more, and smaller, suckers (including hectocotylized arms); 3) more gill lamellae; 4) different funnel organ morphology (*M. leioderma* has medial limbs of its funnel organ longer than broad marginal limbs); 5) a relatively shorter ligula with longer calamus; and 6) in coloration, and lacking of a lateral skin fold and papillae (Table 9) [26, 32] [this study].

#### *Muusoctopus sibiricus* (Løyning, 1930) [62]

**Fig. 14:**
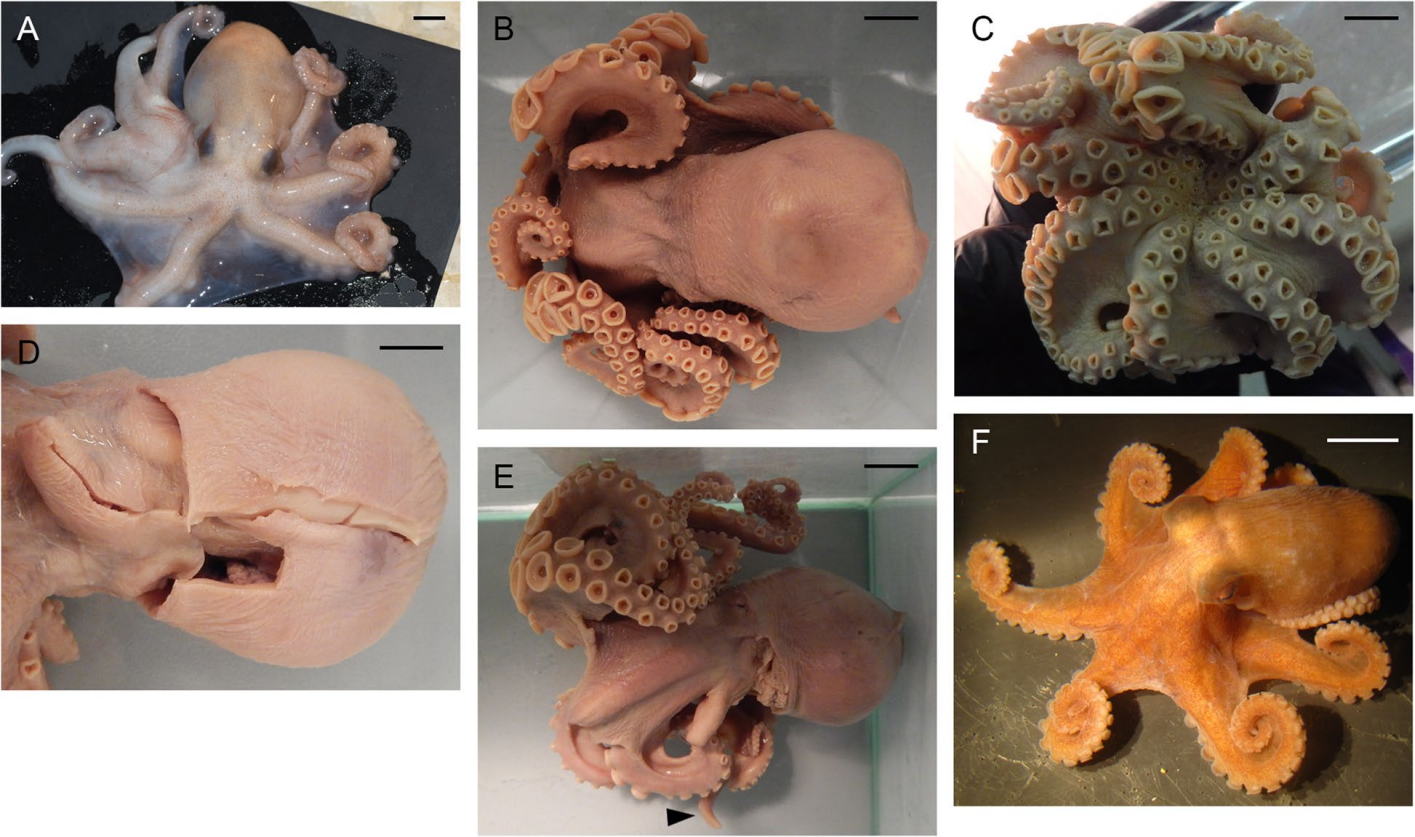
*Muusoctopus sibiricus* (Løyning, 1930) [62]. External view. **a**–**d**, LS-L-3 (pre-spent male, mantle length (ML) 38 mm, Laptev Sea, live (**a**) and fixed (**b**–**d**)): dorsal (**a**, **b**) and ventral (**c**, **d**) view, mantle cut and piece taken for molecular analysis in **d**; **e**, ESS-A-19 (mature male, ML 39 mm, the East-Siberian Sea, fixed): lateral view; **f**, LS-O-22 (not analyzed; Laptev Sea, live): dorsal view. Arrowhead indicates the hectocotylus in male. Scale bars = 10 mm

**Fig. 15:**
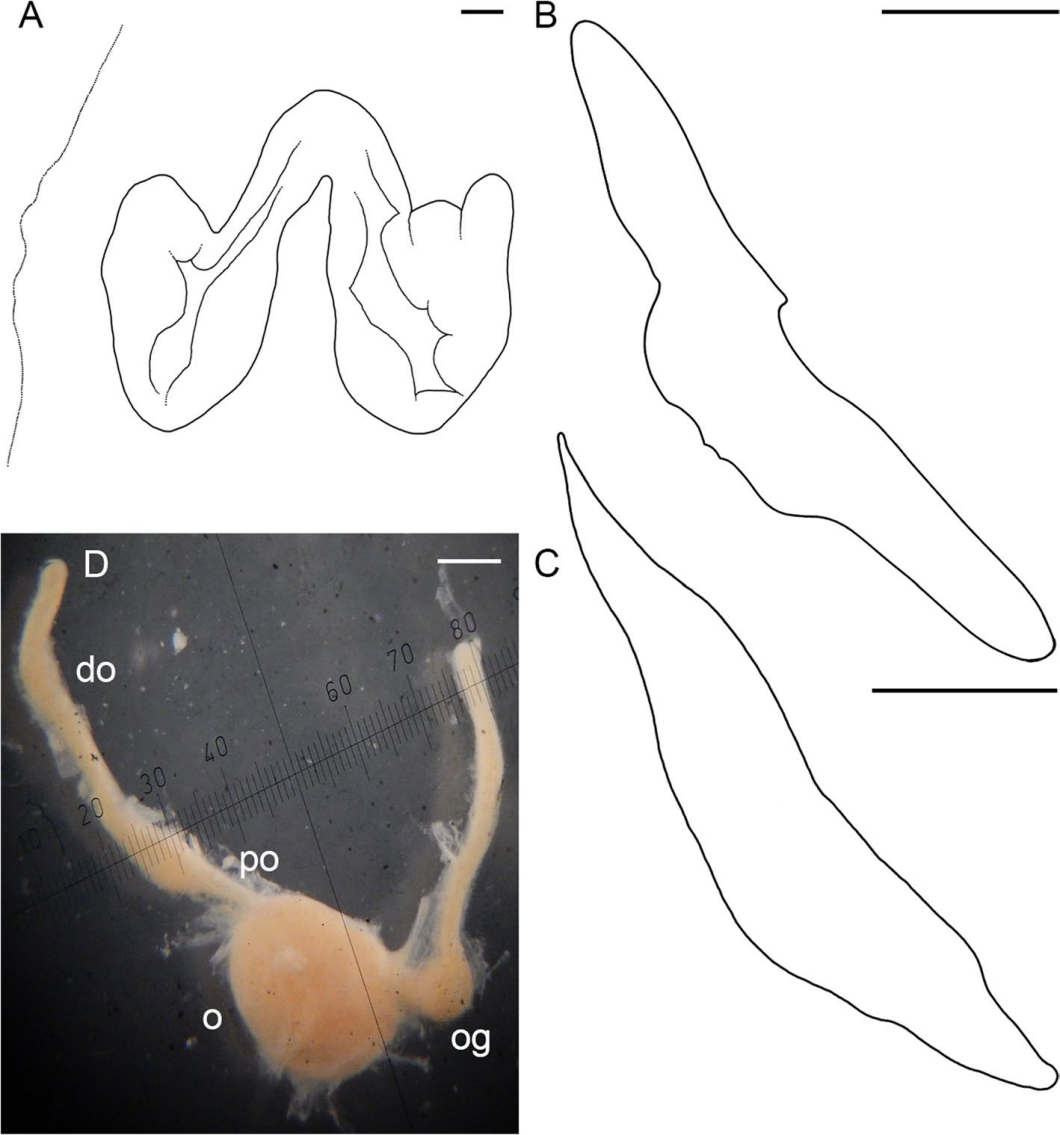
*Muusoctopus sibiricus* (Løyning, 1930) [62]. General anatomy and female reproductive anatomy. **a**, LS-L-3 (pre-spent male, mantle length (ML) 38 mm, the Laptev Sea): funnel organ (**a**) and stylet (**b**); **c**, ESS-A-19 (mature male, ML 39 mm, the East-Siberian Sea): stylet; **d**, ESS-A-51 (early immature female, ML 14 mm, the East-Siberian Sea): female reproductive tract. Scale bars = 1 mm. Abbreviations: do, distal oviduct; o, ovary; og, oviducal gland; po, proximal oviduct

**Fig. 16:**
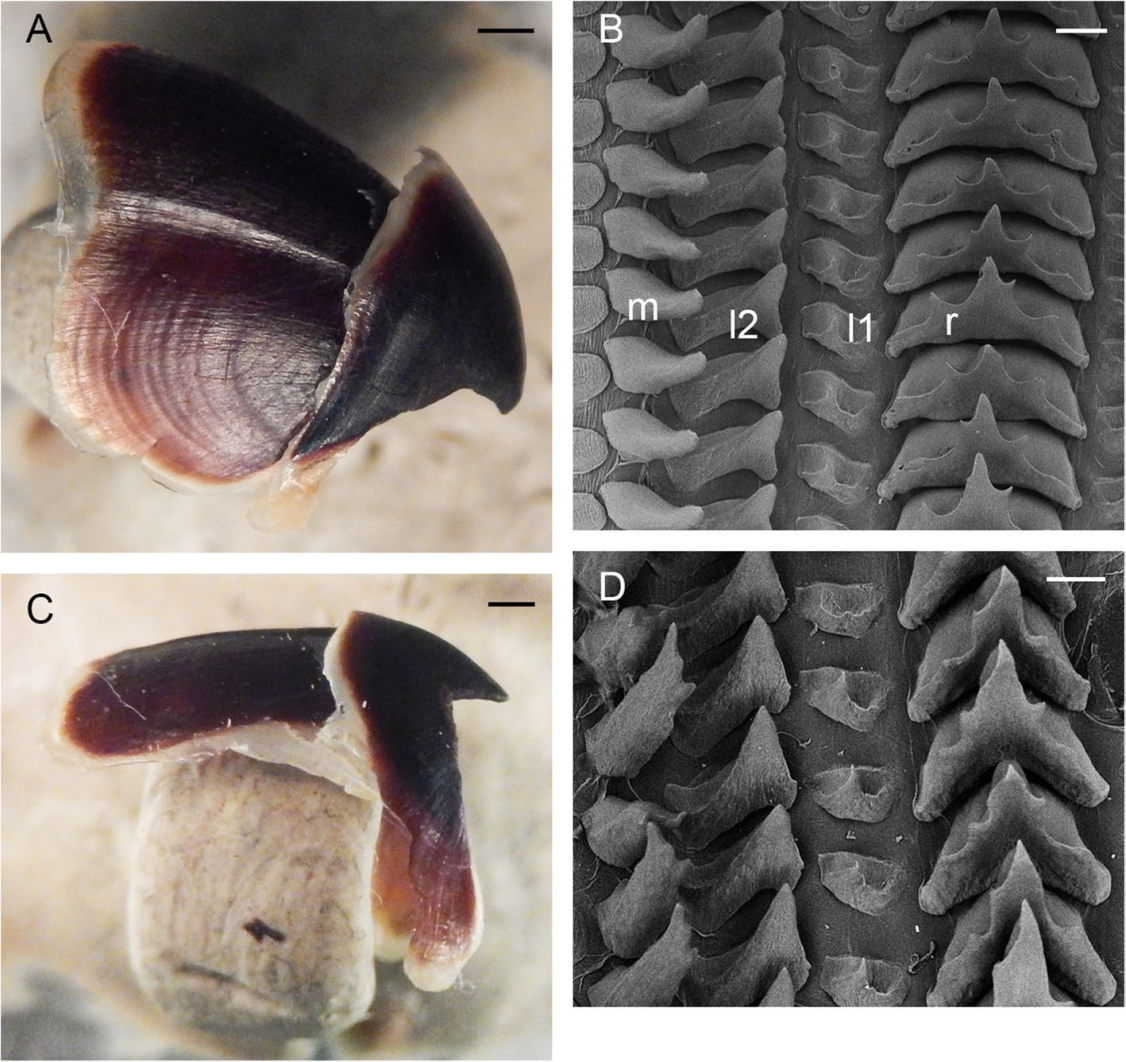
*Muusoctopus sibiricus* (Løyning, 1930) [62]. Beak and radula. **a**–**c**, LS-L-3 (pre-spent male, mantle length (ML) 38 mm, Laptev Sea): upper (**a**) and lower (**b**) beak, and unworn section of radula (**c**); **d**, ESS-A-19 (mature male, ML 39 mm, East-Siberian Sea): unworn section of radula. Scale bars: **a**, **c** = 1 mm, **b**, **d** = 100 µm. Abbreviations: l1, first lateral tooth; l2, second lateral tooth; m, marginal tooth; r, rachidian tooth

**Fig. 17:**
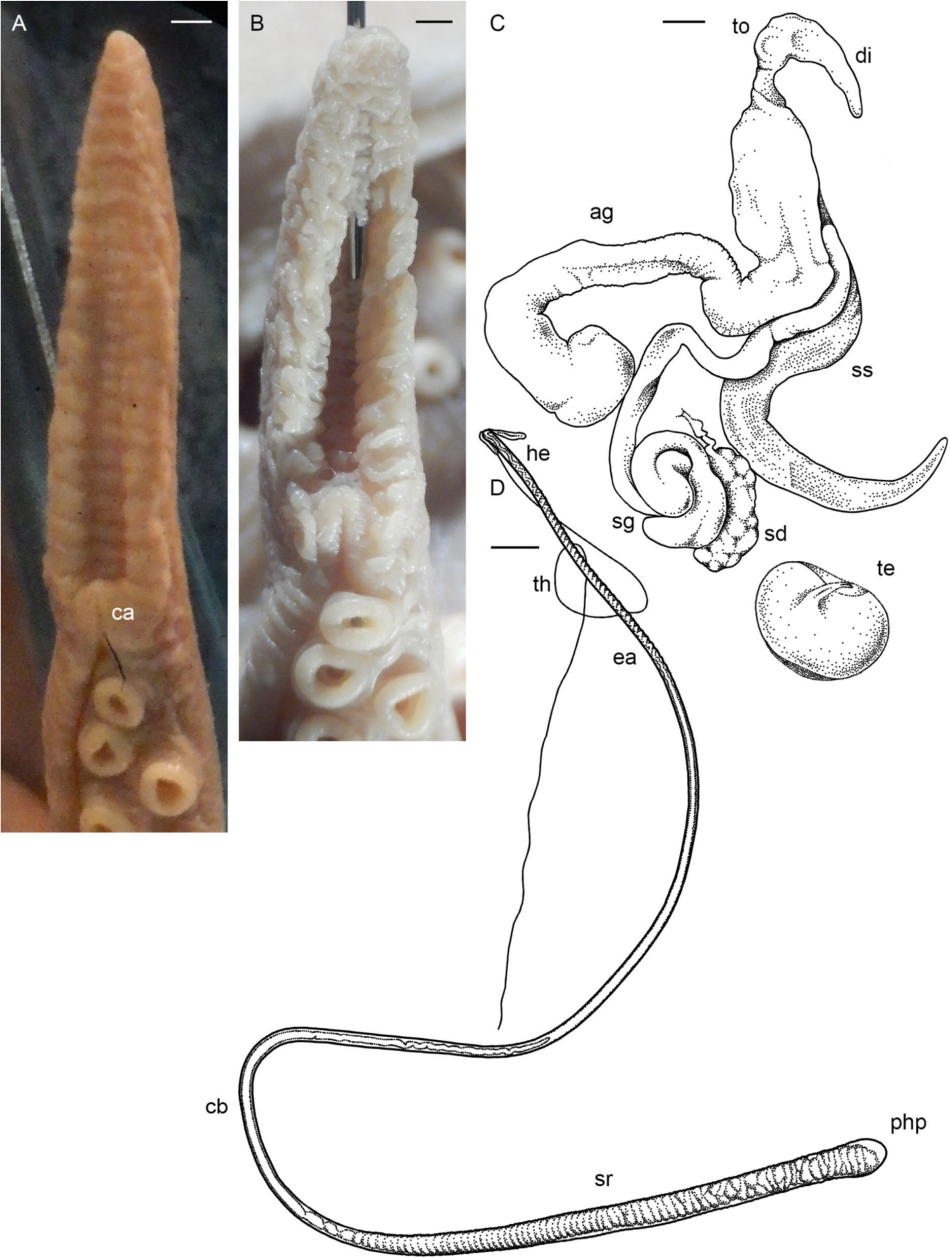
*Muusoctopus sibiricus* (Løyning, 1930) [62]. Hectocotylus and male reproductive anatomy. **a**, ESS-A-19 (mature male, mantle length (ML) 39 mm, the East-Siberian Sea): hectocotylus; **b**–**d**, LS-L-3 (pre-spent male, ML 38 mm, the Laptev Sea): hectocotylus (**b**), spermatophoric complex (**c**), dissected, ventral view, and spermatophore (**d**). Scale bars: **a**, **b**, **d** = 1 mm, **c** = 5 mm. Abbreviations: ag, accessory gland; ca, calamus; cb, cement body, di, diverticulum; ea, ejaculatory apparatus; he, head; sd, sperm duct; sg, spermatophoric glands; sr, seminal reservoir; ss, spermatophoric sac; te, testis; th, thread; to, terminal organ

(Tables 3, 4, 8; SM.01 Table S4; Figs. 14, 15, 16 and 17).

**Table 8:** Data on mature and pre-spent male individuals of *Muusoctopus sibiricus* (Løyning, 1930) [62]. Immature individuals are detailed in SM.01 Table S4. Both individuals are males

Individuals/character	LS-L-3	ESS-A-19
Area	LS	ESS
Maturity stage	Pre-spent (V_3_)	Mature (V_2_)
ML, mm	38	39
TL, mm	179	177
Ventral ML, mm	37	36
Mantle width, mm	40	35
Head length, mm	11	10
Head width, mm	30	22
Eye diameter, mm	8.0	9.0
Lens diameter, mm	2.0	2.4
Funnel length, mm	24.0	21.0
Free funnel length, mm	11.0	11.0
Web depth, mm (min – max)	26–38	26–44
Web formula	c > a = b > d > e	b > d > a = c > e
Arm length, mm (min – max)	122–130	112–128
Arm formula	2 > 1 = 3 > 4	1 > 2 > 3 > 4
Sucker count (min – max)	84–88	84–94
Normal sucker diameter (max), mm	4.0	4.0
Enlarged suckers, diameter (min – max), mm and location	6.0–9.0; 7–12 pairs	5.0–8.0; 8–12 pairs
Gill length, mm	16	19.0
Gill lamellae count, outer/inner	10/10	10/10
Gill lamellae count, inner	10	10
Hectocotylized arm length, mm	123	112
Hectocotylized arm sucker count	60	64
Ligula length, mm	14.5	15.0
Ligula width, mm	3.5	3.0
Calamus length, mm	2.5	2.3
Number of spermatophores	52	49
Spermatophore length, mm (min – max)	61.1–63.5	46.3–56.2

ML, mantle length; TL, total length; LS, Laptev Sea; ESS, East Siberian Sea

##### Synonymy

*Benthoctopus sibiricus* Løyning, 1930 [62]: 1, pls. I, II, text-figs 1, 2; Robson, 1932 [43]: 230; Grimpe, 1933 [45]: 496; Kondakov et al., 1981 [63]: 42, figs 1–3a; Nesis, 1987a [49]: 316, figs 84A, 84B; Nesis, 1987b [50]: 125; Nesis, 2001 [51]: 7.

*Muusoctopus sibiricus* (Løyning, 1930) [62] – Xavier et al*.*, 2018 [86]: 5.

Not *Benthoctopus sibiricus* Løyning, 1930 [62] – Bizikov, 2004 [94]: 35, Figs. 23A, 23B, 24, 25.

##### Material examined

Laptev Sea (LH KFU): ♂V_3_, ML 38 mm, LS-L-3, Stn L-3, 74.91°N, 130.28°E, 33 m, 9 September 2014, sequenced for *COI*.

East Siberian Sea (LH KFU): ♂V_2_, ML 39 mm, ESS-A-19, Stn A-19, 76.09°N, 174.69°E, 36 m, 21 August 2014; ♀I, ML 14 mm, ESS-A-51, Stn A-51, 77.35°N, 146.89°E, 38 m, BT -0.82 °C, 25 August 2014; ESS-A-27, juvenile, ML 10 mm, Stn A-27, 76.40°N, 143.80°E, 29 m, BT -1.00 °C, 30 August 2014 (ex stomach contents of *Gymnocanthus tricuspis* (Reinhardt, 1830) [95], length 175 mm, weight 72 g, age 6 + ; partially digested).

##### Additional material examined

See SM.01.

##### Diagnosis

Small (maximum ML 59 mm), brick-red or bright orange octopods. Smooth skin with minute folds dorsally on mantle and head, visible in both live and fixed individuals; with ovoid body, and arms ~ 3.0 times ML. Suckers large, biserial, closely set, with pairs 7–12 enlarged in mature males; with 84–104 suckers on unmodified arms, and 60–64 on hectocotylus. Hectocotylized arm as long as or longer than opposite arm. Ligula large, narrow, tapering gradually, without transverse ridges, but with 27–29 low rugae. Calamus small and pointed. Funnel long, free from ventral surface of head for about half its length. Funnel organ W-shaped, with medial limbs much longer than very broad marginal limbs. Gills very long, with 10 or 11 (mode 10) outer and 10 inner lamellae per demibranch. Stylets vestigial. Anal flaps and ink sac absent; ink duct vestigial. Rachidian with 4–5 asymmetrical cusps, with 3–6 seriation; second lateral occasionally with small secondary cusp. Very long and slender spermatophores, up to 52. Female with up to 136 oocytes.

##### Description

Counts and measurements for the species are given in Tables 8 and S4, and indices are given in Table 9.

**Table 9:** Comparison of the Arctic and northern North Atlantic species of *Muusoctopus* Gleadall, 2004 [20]. Immature individuals are not used (except for *Muusoctopus* sp. 1, where only immature individuals are known, and *M. leioderma* (Berry, 1911) [64], where it is impossible to separate them from the rest in Akimushkin (1965) [66], Hochberg (1998) [67] and Ibanez et al*.* (2016) [92]). Values are minimum to maximum (mean ± SE), where applicable (except for *M. leioderma* (Berry, 1911) [64], where values from Akimushkin (1965) [66], Hochberg (1998) [67] and Ibanez et al*.* (2016) [92] are impossible to recalculate due to lack of individuals’ details). ML, mantle length

Species/character		*Muusoctopus* *aegir* sp. nov.	*Muusoctopus* *johnsonianus*	*Muusoctopus* *leioderma*	*Muusoctopus* sp. 1	*Muusoctopus* *normani*	*Muusoctopus* *sibiricus*
**Habitat depth, m**	**Original data**	86–2442 (579.4 ± 52.4)	540–1957 (1470.6 ± 230.4)	No data	101–450.5 (314.8 ± 108.2)^a^	No data	29–255 (58.2 ± 16.6)
	**Literature data**	86–2000^b^	797–2540^c^	38–1760^d^	No data	500–1843^f^	30–220^ g^
**Habitat temperature,** **° C**	**Original data**	-1.31 to 6.90 (0.41 ± 0.30)	2.34–5.50 (3.18 ± 0.48)	No data	1.97–2.00^e^	No data	-1.74 to 0.28 (-1.23 ± 0.18)
	**Literature data**	-0.9^b^	No data	-1.0 to 4.9^d^	No data	No data	-1.4 to 1.6^ g^
**Sources of information on morphology**		This study (*n* = 25); Nesis (2001) (*n* = 2)	This study (*n* = 3); Allcock et al*.* (2006) (*n* = 3); Gleadall (2013) (*n* = 1)	Kondakov (1941) (*n* = 3; 2 = juv.); Akimushkin (1965) (*n* not stated); Nesis (1987) (*n* not stated); Hochberg (1998) (*n* not stated); Ibanez et al. (2016) (*n* not stated)	This study (*n* = 3); A. L. Allcock (unpubl. data) (*n* = 1)	Allcock et al. (2006) (*n* = 16); Barrat et al*.* (2007) (*n* = 5); Gleadall (2013) (*n* = 1)	This study (*n* = 2); Løyning (1930) (*n* = 1); MacGinitie GE (1955) + MacGinitie N (1959) (*n* = 1); Kondakov et al (1981) (*n* = 2); Nesis (2001) (*n* = 1)
**ML, mm**		20–52 (32.3 ± 1.8)	47–113 (78.6 ± 10.2)	42–100	22–31 (26.5 ± 2.3)	50–107 (73.1 ± 3.2)	26–59 (43.4 ± 6.0)
**TL, mm**		96–235 (141.6 ± 7.8)	255–510 (354.2 ± 49.8)	Up to 210	99–148 (121.7 ± 14.3)	320–648 (431.6 ± 21.8)	94–253 (187.6 ± 27.8)
**Ventral ML, mm**		17–49 (27.8 ± 1.9)	40–89 (64.7 ± 8.5)	No data	17–26 (20.7 ± 2.7)	43–61 (50.5 ± 2.1)	36–37^e^
**Mantle width, % ML**		87.5–140.0 (111.3 ± 3.4)	83.1–100.0 (91.0 ± 2.4)	63.0–89.0	87.8–109.7 (102.0 ± 5.1)	43.3–78.8 (69.7 ± 2.2)	54.0–105.3 (85.8 ± 11.1)
**Head width**	**% ML**	50.0–100.0 (78.7 ± 2.6)	63.7–106.1 (81.0 ± 6.1)	No data	74.2–86.7 (80.2 ± 2.8)	36.4–69.4 (56.4 ± 2.0)	54.0–78.9 (62.3 ± 5.7)
	**% mantle width**	48.0–85.7 (71.3 ± 2.2)	75.8–106.1 (88.4 ± 4.8)	78.1–105.0	67.6–95.0 (79.5 ± 5.9)	67.7–127.6 (81.8 ± 3.3)	62.9–75.0 (68.9 ± 6.1)
**Eye diameter, % ML**		26.4–40.7 (32.5 ± 0.8)	32.6–55.1 (47.0 ± 7.2)	No data	27.3–30.4 (28.7 ± 0.9)	No data	21.1–23.1^e^
**Lens diameter, % eye diameter**		20.0–38.9 (28.8 ± 1.3)	28.0–31.0 (29.6 ± 0.9)	No data	28.6–35.3 (32.4 ± 2.0)	No data	25.0–26.7^e^
**Funnel length, % ML**		28.8–56.7 (42.1 ± 1.6)	39.8–55.3 (46.8 ± 2.2)	30.0–58.0	39.1–45.2 (42.1 ± 1.3)	20.9–46.3 (35.1 ± 1.5)	53.8–63.2^e^
**Free funnel length,** **% funnel length**		45.5–57.1 (52.9 ± 0.7)	50.0–75.0 (64.3 ± 3.5)	No data	53.8–78.6 (65.0 ± 5.2)	19.2–66.7 (40.2 ± 2.9)	45.8–52.4^e^
**Funnel organ shape**		W; medial and marginal limbs of same length, or medial can be slightly longer; marginal limbs broad	W; medial and marginal limbs of same length, or marginal can be slightly longer; marginal limbs moderately broad	W; medial limbs longer than marginal limbs; marginal limbs broad	VV; medial limbs slightly longer than marginal limbs; marginal limbs relatively narrow	VV; medial and marginal limbs of same length, or medial can be slightly longer; marginal limbs relatively narrow	W; medial limbs much longer than marginal limbs; marginal limbs very broad
**Web depth, % longest arm**		10.4–35.6 (25.5 ± 1.1)	17.9–32.4 (25.1 ± 1.3)	14.0–24.0	14.0–25.7 (20.1 ± 2.2)	10.8–25.0 (17.7 ± 0.6)	20.0–34.4 (27.3 ± 1.1)
**Web formula**		b > c > a > d > e	b > c > d > a > e	b > a = c > d > e or c > b > d > a > e or a > b > c > d > e	c > b > d > a > e	c > b > a > d > e	b > c > a > d > e
**Arm length, % ML**		234.6–376.2 (313.7 ± 6.8)	271.9–456.9 (351.3 ± 14.4)	224.0–374.0	245.5–373.9 (293.2 ± 17.7)	328.1–541.6 (417.5 ± 12.6)	230.0–350.0 (303.6 ± 28.4)
**Arm formula**		1 > 2 > 3 > 4	2 > 1 > 3 > 4	1 > 2 > 3 > 4	1 > 2 > 3 > 4	1 > 2 > 3 > 4	2 > 1 > 4 > 3
**Sucker count**		84–120 (95.5 ± 1.5)	128–164 (140.3 ± 3.3)	80–102	81–118 (99.9 ± 5.3)	134–199 (170.0 ± 4.8)	84–104 (88.1 ± 1.4)
**Sucker diameter, % ML**		6.3–11.7 (8.9 ± 0.3)	7.1– 9.0 (8.4 ± 0.3)	7.0–11.0	8.7–10.0 (9.4 ± 0.3)	4.5–10.6 (7.0 ± 0.4)	7.8–16.7 (11.3 ± 1.9)
**Enlarged suckers, diameter** **(% ML) and location**		Enlarged suckers absent	Enlarged suckers absent	Enlarged suckers absent	No data (as only immature individuals are known)	Enlarged suckers absent	7–12 pairs; 20.5–25.0 (23.1 ± 1.3)
**Gill length, % ML**		21.9–44.0 (34.4 ± 1.3)	25.8–31.9 (29.4 ± 0.8)	27.0	39.1–45.0 (41.9 ± 1.7)	20.9–32.0 (25.3 ± 0.7)	42.1–48.7^e^
**Gill lamellae, outer/inner**		8–9 (mode 8)/ 7–8 (mode 7)	8–11 (mode 10)/ 8–11 (mode 9)	11–12/? (whole gill 16–20)	8–10 (mode 10)/ 8–9 (mode 9)	7–10 (mode 9)/ 6–9 (mode 8)	10–11 (mode 10)/10
**Stylets**		Absent	Present	Present (vestigial?)	No data	Present	Vestigial
**Vestigial ink duct**		Absent	Present	Present	No data	Present	Present
**Hectocotylized arm length**	**% ML**	177.8–282.6 (233.8 ± 6.5)	208.0–299.0 (268.6 ± 12.9)	127.0–139.0	246.7–256.5^e^	189.0–261.2 (220.3 ± 6.3)	287.2–323.7^e^
	**% opposite arm**	55.2–80.7 (71.5 ± 2.2)	65.3–88.4 (78.5 ± 4.2)	69.0–89.0	79.7–93.7^e^	51.0–63.7 (58.0 ± 1.7)	91.1–123.4 (102.6 ± 7.2)
**Hectocotylized arm** **sucker count**		46–56 (52.0 ± 1.1)	67–71 (68.3 ± 0.6)	47–61	56–66^e^	61–72 (65.8 ± 1.3)	60–64 (62.7 ± 1.3)
**Ligula length, % hectocotylized** **arm length**		8.0–14.1 (10.3 ± 0.5)	3.9–10.6 (6.3 ± 1.0)	11.0–18.0	4.6–6.5^e^	6.3–11.1 (8.6 ± 0.4)	9.0–13.4 (11.5 ± 0.9)
**Ligula width, % ligula length**		45.0–63.3 (55.4 ± 2.1)	48.0–57.1^e^	No data	55.6–62.5^e^	No data	20.0–24.1^e^
**Calamus length, % ligula length**		27.5–43.3 (36.8 ± 1.4)	28.0–43.5 (36.9 ± 2.8)	12.0–20.0	37.5–66.7^e^	26.7–60.0 (42.3 ± 2.9)	14.3–17.2 (15.6 ± 0.9)
**Number of spermatophores**		5–22 (12.5 ± 2.4)	4–19^e^	No data	No data	3–25 (13.1 ± 2.3)	49–52^e^
**Spermatophore length**	**mm**	27.9–48.0 (39.8 ± 0.5)	104.0–120.0^e^	No data	No data	75.0–119.0 (92.8 ± 4.4)	46.3–63.5 (57.9 ± 1.1)
	**% ML**	78.0–135.9 (108.1 ± 1.8)	92.0–114.3^e^	No data	No data	97.5–142.2 (121.0 ± 5.9)	118.7–167.1 (150.6 ± 3.3)
**Fecundity of females**		65–168 (99.5 ± 6.8)	227–300 (259.0 ± 21.5)	No data	No data	70–342 (218.9 ± 34.2)	136
**Ripe oocyte length**	**mm**	12.5–13.0^e^	No data; large vitellogenic oocytes are 17.0–22.0 (mean 20.0) mm	14.0–17.0	No data	15.0–22.0 (18.0 ± 0.7)	No data
	**% ML**	52.1–54.2^e^	No data; large vitellogenic oocytes 19.1–24.7 (mean 22.5)	No data	No data	21.1–40.7 (27.8 ± 2.3)	No data

^a^data for individual USNM 574859 provided by A. L. Allcock (A. L. Allcock, unpubl. data) included; ^b^Hoyle 1886 [34], Appelløf 1893 [36], Russell 1909 [41], 1922 [42], Grieg 1933 [44], Nesis 2001 [51], Taite et al*.* 2023 [57]; ^c^Collins et al*.* 2001 [22], Allcock et al*.* 2006 [26], Strugnell et al*.* 2009 [56], Gleadall 2013 [32], Luna et al*.* 2021 [55], Pratt et al*.* 2021 [25], Taite et al*.* 2023 [57]; ^d^Kondakov 1941 [65], Akimushkin 1965 [66], Nesis 1987 [49], Hochberg 1998 [67]; ^e^*n* = 2; ^f^Collins et al*.* 2001 [22], Allcock et al*.* 2006 [26], Strugnell et al*.* 2009 [56], Gleadall 2013 [32], Taite et al*.* 2023 [57]; ^g^Løyning 1930 [62], MacGninitie GE 1955 [70], MacGinitie N 1959 [71], Kondakov et al*.* 1981 [63], Nesis 2001 [51], Bluhm et al*.* 2004, Furuya 2010 [72]

The following description is based on two studied males (mature and pre-spent), and maturing to mature individuals from Løyning [62] (*n* = 1), MacGinitie GE [70] and MacGinitie N [71] (*n* = 1; the same individual), Kondakov et al*.* [63] (*n* = 2) and Nesis [51] (*n* = 1). Species small, ML 26–55 mm (39.5 ± 6.0 mm), TL 94–253 mm (187.6 ± 27.8 mm) (Fig. 14; Tables 8, 9). Ventral ML 1 and 3 mm shorter than dorsal ML (*n* = 2). Mantle ovoid, width 85.8% ± 11.1% ML. Head width 68.9% ± 6.1% mantle width (Fig. 14). Eyes less prominent than in other North Atlantic and Arctic *Muusoctopus;* their diameter 21.1% and 23.1% ML (*n* = 2) (Fig. 14). Funnel long (53.8% and 63.2% ML; *n* = 2), strongly tapered, free from ventral surface of head for almost half its length (45.8% and 52.4% funnel length; *n* = 2). Funnel organ W-shaped, with medial limbs much longer than marginal limbs, and marginal limbs very broad (Fig. 15a). Arms ~ 3.0 times ML (Fig. 7), subequal in length, with formula 2.1.4.3. Suckers (84–104 (88.1 ± 1.4)) biserial to arm tip, large (11.3% ± 1.9%, to 16.7% ML), closely set (Fig. 14); pairs 7–12 enlarged in mature and pre-spent males: 23.1% ± 1.3% ML (Fig. 14); enlarged suckers absent in maturing female [63]. Web deep (27.3% ± 1.1% longest arm length), with sectors B and C deepest, and D and E most shallow.

Gills very long (42.1% and 48.7% ML; *n* = 2), with 10 or 11 (mode: 10) outer and 10 inner lamellae per demibranch. Stylets non-calcareous, vestigial (Fig. 15b, c), of length 4.4 and 4.6 mm (12.2% and 12.4% ML), width 0.8 and 0.9 mm (2.2% and 2.4% ML) (*n* = 2). Upper beak with hooked, broad rostrum (Fig. 16a); lower beak with straight and relatively small rostrum (Fig. 16c); both typically *Muusoctopus*. Anterior salivary glands medium-sized (21.1% and 22.1% ML; *n* = 2), discoid. Posterior salivary glands large (28.0% and 28.6% ML; *n* = 2), approximately triangular. Crop diverticulum well developed. Presence of rectum loop not examined. Ink sac absent; vestigial ink duct present on ventral surface of digestive gland, connects to distalmost rectum. Anal flaps absent. Radula with nine elements per transverse row. Rachidian with 4–5 cusps, the central largest; lateral cusps asymmetrical, with 4–6 seriation (Fig. 16b, d) (3–4 according to [63]). Marginal and lateral teeth mostly unicuspid: small second cusp found in one individual on second right lateral, and the same is known in one individual on second left lateral in Løyning [62]; marginal teeth curved. Marginal plates well developed (Fig. 16b, d).

Male third right arm hectocotylized (Fig. 14e), of length 287.2% and 323.7% ML (*n* = 2) or 102.6% ± 7.2% opposite arm, with 60 to 64 (62.7 ± 1.3) suckers. Ligula large, 9.0–13.4% (11.5% ± 0.9%) of arm length, narrow, 20.0% and 24.1% ligula length (*n* = 2), tapering gradually. Ligula with distinct margins, well-marked shallow groove without transverse ridges, but with 27 or 29 low rugae; with groove and margins basally of comparable width (Fig. 17a, b). Calamus small, 14.3–17.2% (15.6% ± 0.9%) ligula length, and pointed (Fig. 17a, b). Spermatophoric complex accessory gland longer than spermatophoric sac (Fig. 17c), both longer than ML (accessory gland > 2 × ML). Length of terminal organ with diverticulum 40% and 45% ML (*n* = 2). Spermatophores 49 (in mature male) and 52 (in pre-spent male) (Tables 3, 8), very long, 118.7–167.1% (150.6% ± 3.3%) ML; slender, width 0.6–0.8 (0.7 ± 0.03) mm (Fig. 17d). Sperm cord width 0.15–0.20 mm, forming 59–92 (79.2 ± 5.8) whorls. Seminal reservoir length 25.5–37.4% (29.5% ± 2.3%) spermatophore length (Table 3); ejaculatory tube comprises longest part of spermatophore (Fig. 17d; Table 3). Immature female reproductive system (Fig. 15d) with 136 oocytes (Table 4; SM.01 Table S4).

Skin smooth, with minute folds on live individuals dorsally on mantle and head, more apparent when fixed (Fig. 14). Live animal color from brick-red or bright orange with small whitish spots (Fig. 14f) (also see [63]) to uniformly pale (Fig. 14a). Fixed individuals light violet-brown (Fig. 14) (also see [63]).

##### *COI* barcode

The sequence for individual LS-L-3 is deposited in GenBank, accession number OM791385.

##### Distribution

The Laptev, East Siberian, Chukchi, and Beaufort Seas (Fig. 1), east of 105.63°E (southwestern Laptev Sea); how far east this species occurs in the Beaufort Sea, and whether it reaches the western margin of the Canadian Arctic Archipelago, are unknown [49–52, 63] [this study]. Habitat depth is 30–220 m judging from literature, with the associated bottom temperatures –1.4–1.6 °C [51, 62, 63, 70–72, 74], and 29–255 m (58.2 ± 16.6 m) and –1.74–0.28 °C (–1.23 ± 0.18 °C), respectively, according to our data.

##### Biology and ecology

Mature male with 49 spermatophores, and pre-spent male with 52 spermatophores. An ontogenetic increase in the spermatophore size observed in mature male: older spermatophores (in the terminal organ) were shorter than younger spermatophores (in the spermatophoric sac) by 21.4%, while spermatophore width remained the same. Among spermatophore parts, the seminal reservoir increases most (46.7%), followed by the ejaculatory apparatus, 6.1%, with the head and posterior hollow part remaining similarly sized; the cement body decreases by 13.2%. Seminal reservoir width does not show an ontegenetic increase, and seminal reservoir volume increases by 78.6%.

##### Remarks

Reports of *M. sibiricus* by Bizikov [94] from the Bering Sea continental slope are in fact referable to another Pacific *Muusoctopus* species, but not to *M. sibiricus*: these individuals are larger than *M. sibiricus* and *M. leioderma* (Table 9) [63, 65–67, 92] [this study]; and their stylets differ from those of *M. sibiricus* in shape [94] [this study]. The stylets of *M. sibiricus* in this study are considered vestigial following Bizikov [94] [p. 39], because they are very small, and the left and right stylets are highly variable within the same individual and among the individuals.

A diagnosis of *M. sibricus* was absent in the literature prior to this study. *Muusoctopus sibiricus* differs from other Arctic and North Atlantic *Muusoctopus* species, excepting *M. leioderma*, in having skin folds in live animals, and otherwise in: 1) funnel organ morphology; 2) a presence of enlarged suckers, and generally larger suckers than other regional species; 3) proportionally longer gills (values are overlapping with *M. aegir* and *Muusoctopus* sp. 1) with 10 or 11 (mode 10) outer and 10 inner lamellae per demibranch; 4) vestigial stylets; 5) occasionally having a bicuspid second lateral and rachidian with 3–4 cusps; 6) proportionally the longest hectocotylized arm relative to ML and opposite arm, with a very long and narrow ligula and short calamus; and 6) more and larger spermatophores (although data are lacking on spermatophore number in *M. leioderma* and *Muusoctopus* sp. 1), of different proportions to those of *M. aegir* (Table 9) [26, 32, 62, 63, 65–67, 92] [this study].

### DNA barcoding

There were no sequences of *M. sibiricus* in either GenBank (https://www.ncbi.nlm.nih.gov/genbank) or BOLD (https://www.boldsystems.org/) databases prior to this study. The analyses of available sequences of *Muusoctopus*, *Benthoctopus* and *Vulcanoctopus* González & Guerra, 1998 (in González et al. 1998) [96] from these databases vs. our sequence of *M. sibiricus* support recognizing it as a distinct species (SM.01 Fig. S1).

## Discussion

### Species identification

The main characters used to identify the Arctic and northern North Atlantic species of *Muusoctopus* are provided in Table 9, with the differences among species reported in the Remarks sections for each species. The most frequently cited characters in species descriptions are relative mantle width; sucker diameter; arm, hectocotylized arm, opposite arm, gill, ligula and calamus lengths; funnel organ morphology; sucker (normal and hectocotylized arms) and gill lamellae counts; and stylet morphology (present/vestigial/absent). *COI* sequences may differentiate species, and these are currently available for *M. aegir* (as *Muusoctopus* sp. [57]; see Remarks section for this species for explanation of why we think it is *M. aegir*), *M. johnsonianus* [26, 57, 97], *M. normani* [26, 57] and *M. sibiricus* [this study].

### Biogeography and phylogeography

Eleven species of cephalopods complete their entire lifecycle in the Arctic: the squid *Gonatus fabricii* (Lichtenstein, 1818) [98], sepiolids *Rossia palpebrosa* Owen, 1835 [99], *R. moelleri* Steenstrup, 1856 [100] and *R. megaptera* Verrill, 1881 [87], and octopods *Cirroteuthis muelleri* Eschricht, 1836 [101], *B. arcticus*, *B. bairdii*, *B. pugniger*, *M. sibiricus*, *M. leioderma* and *Muusoctopus* sp., which is described here as *M. aegir* [75, 86, 93, 102]. Here, we also report *Muusoctopus* sp. 1, apparently new species from the Canadian Arctic Archipelago and Baffin Bay. Of these species, *R. moelleri* and *M. sibiricus* are the most shallow and cold-water species among their respective genera [50, 51, 86, 93]. Little is known of the depth distribution of *Muusoctopus* sp. 1, but *M. aegir* ascends from deeper to shallower depths towards the pole, frequenting mean depths of 951 m in Icelandic waters, 518 m in the Barents Sea, and 403 m in the Kara Sea (polar emergence). Both *B. arcticus* and *C. muelleri* also manifest such polar emergence [23, 50, 51, 103].

Of the 12-now recognized Arctic cephalopod taxa, seven are incirrates, and four of them of *Muusoctopus* taxa; only polar emergence is present [23, 50, 51, 86, 93, 103]. In contrast, of the 54 cephalopod taxa reported from Antarctic waters, 27 are incirrates, of which 17 manifest either polar emergence or submergence (the opposite trend) [86, 97, 104]. *Muusoctopus* manifest polar emergence at both poles [51, 97] [this study].

Results from non-molecular biogeography methods suggest that *M. sibiricus* originated in the North Pacific, and *M. aegir* (at the time as *Be. piscatorum*) in the North Atlantic [50–52]. These indicate that *Muusoctopus* entered the Arctic independently from the Atlantic and Pacific. Molecular methods suggest that the origins of the *Muusoctopus* is either from the North Pacific [32, 97] or North Atlantic [92], with different dispersal thereafter. No molecular study has included Arctic material. However, independent appearances of *Muusoctopus* species from the Atlantic and Pacific Arctic are congruent with molecular genetics results [32, 92, 97].

It is known that the ink sac was lost independently within deep-sea Incirrata [15]. In *Sasakiopus salebrosus* (Jorgensen et al*.*, 2010) [105], there is both a vestigial functional ink sac and ink duct [105]. In Arctic *M. sibiricus*, Arctic and northern North Pacific *M. leioderma*, North Atlantic *M. johnsonianus* and *M. normani*, and South Atlantic *M. bizikovi* Gleadall, Guerrero-Kommritz, Hochberg & Laptikhovsky, 2010 [31], there is no ink sac, but a non-functional vestige of the ink duct is present [31, 32, 67] [this study]. Stylets have seldom been reported in *Muusoctopus* species descriptions [29, 43, 77, 106], however, vestigial stylets in East Arctic *M. sibiricus* and North Pacific *Muusoctopus* sp., and the absence of stylets in the West Arctic *M. aegir* and the well-developed stylets in several of the Atlantic and Pacific species [26, 31, 94, 107] [this study] suggest an independent reduction of stylets in ancestral *Muusoctopus* taxa. Shared common vestigial absences of a particular character among many closely related species are more likely explanation than multiple losses [108], which is supposed to be the case for ink sac, but not stylets, in *Muusoctopus*.

### Reproductive biology and ecology

The spermatophore morphology of *Muusoctopus* [26, 29, 31, 107] [this study] differs from that of *Bathypolypus* [23]. In some species of *Muusoctopus*, the ejaculatory apparatus is the longest part of the spermatophore, and in others it is the seminal reservoir [26, 29, 31, 107] [this study]. An ontogenetic increase in the spermatophore size (with spermatophores produced later during ontogenesis being larger than those produced earlier), and production of the tentative spermatophores prior to the onset of normal spermatophorogenesis are known for squids and sepiolids [80, 109–117], and for cirrate octopods [118]. Herein we report an ontogenetic increase in the spermatophore size for incirrate octopods (in *M. aegir* and *M. sibiricus*), in addition to production of the tentative spermatophores prior to the onset of normal spermatophorogenesis (in *M. aegir*).

The spermatophore number in *Muusoctopus* (typically to 20–25) is greater than in *Bathypolypus* (to 6) [23, 26, 32] [this study]. In *M. sibiricus*, we report up to 52 spermatophores, which are relatively longer than in other species of *Muusoctopus* [26, 32] [this study], surpassed in length only by some *Enteroctopus* Rochebrune & Mabille, 1889 [119] [120].

Although sperm in the oviducal glands is considered the typical mechanism of incirrate fertilization [121], it has not been recorded in *Muusoctopus*. Here, it is reported for the first time in this genus (in *M. aegir*). Synchronous maturation of the oocytes with their further division into two portions, of which only one undergoes consecutive development, supposedly occurs for the most of deep-sea and Antarctic incirrates, with realized fecundity ranging ~ 40–100% in these species [122–124]. We regard the latter value to be an overestimation because of sample conditions, rendering post-ovulatory follicles similar to resorbed oocytes and vice versa: the realized fecundity is ~ 24–90% in deep-sea and polar squids [125]. Still, low fecundity and large ripe oocytes in deep-sea North Atlantic and Arctic *Muusoctopus* species [26, 90] [this study] conform with known data on the reproductive biology of deep-sea and Antarctic octopods, as does the realized fecundity of *M. aegir* [122–124].

## Conclusions

Two new species of deep-sea octopods of the genus *Muusoctopus* are reported, and a diagnosis for *M. sibiricus* is provided. The key characters and metrics are given in a table to identify these octopods in the northern North Atlantic and Arctic. This resolves a long-standing issue with Arctic non-*Bathypolypus* deep-sea octopods erroneously reported as ‘*Be. piscatorum*’ and otherwise ignored. The unusually large sample size for a deep-sea species (*n* = 37) spanning the area off Iceland to the Kara Sea enabled analysis of biology and ecology of the new species, *M. aegir*. Reproductive biology of *Muusoctopus* octopods is reported, including the original data on realized fecundity and fertilization. Equations for estimating octopod size from beak measurements are provided, which are an invaluable tool in analyses of predator diets.

### Supplementary Information

**Additional file 1.** 

## Data Availability

All data generated or analyzed during this study are included in this published article [and its supplementary information files]. New species nomenclatural acts are registered in ZooBank. Genetic data are deposited in GenBank, NCBI. This article has been registered at Zoobank (urn:lsid:zoobank.org:pub:18E04F52-1AFB-452D-8FD4-936CEF228E6F).
