# Supplementary material for: A review of the genus Muusoctopus (Cephalopoda: Octopoda) from Arctic waters
Source: Zoological Lett. 2023 Nov 16;9:21. doi: 10.1186/s40851-023-00220-x (PMC10655294; doi:10.1186/s40851-023-00220-x)

**SM.01.** Supporting information for ‘A review of the genus *Muusoctopus* (Cephalopoda: Octopoda) from Arctic waters’

ALEXEY V. GOLIKOV*, GUDMUNDUR GUDMUNDSSON, MARTIN E. BLICHER, LIS L. JØRGENSEN, EKATERINA I. KORNEEVA, STEINUNN H. OLAFSDOTTIR, ELENA I. SHAGIMARDANOVA, LEYLA H. SHIGAPOVA, DENIS V. ZAKHAROV, OLGA L. ZIMINA and RUSHAN M. SABIROV

*Corresponding author. E-mail: golikov.ksu@gmail.com

Additional material examined for *Muusoctopus aegir* Golikov, Gudmundsson & Sabirov, **sp. nov.**, *M. johnsonianus* (Allcock, Strugnell, Ruggiero & Collins, 2006) and *M. sibiricus* (Løyning, 1930).

The following individuals are identified from photographs provided to the senior author, and the respective stations are used to estimate depth and habitat temperature ranges for the species.

*Muusoctopus aegir* Golikov, Gudmundsson & Sabirov, **sp. nov.** Iceland: ICL-A11-640-2016, Stn 640, 66.75°N, 12.83°W, 932.5 m, bottom temperature (BT) -0.50°C, 24 October 2016; ICL-A11-580-2016, Stn 580, 66.99°N, 24.92°W, 2442 m, bottom temperature -0.20°C, 18 October 2016; ICL-A11-579-2016, Stn 579, 66.79°N, 24.95°W, 2165 m, BT -0.40°C, 18 October 2016.

Barents Sea: BS-HH-2053-2014, Stn 2053, 78.59°N, 8.33°E, 1021 m, BT 6.90°C, 2 September 2014; BS-HH-2042-2014, Stn 2042, 79.05°N, 8.60°E, 322 m, BT 6.50°C, 1 September 2014; BS-HH-2027-2014, Stn 2027, 80.28°N, 16.70°E, 322.5 m, BT 0.20°C, 27 August 2014; BS-HH-2011-2014, Stn 2011, 79.68°N, 6.65°E, 1010.5 m, BT 4.90°C, 22 August 2014; BS-HH-2009-2014, Stn 2009, 79.68°N, 7.49°E, 802 m, BT 6.90°C, 22 August 2014; BS-348-2013, Stn 348, 82.49°N, 47.24°E, 439.5 m, BT 1.63°C, 16 October 2013; BS-313-2-12, Stn 313, 81.84°N, 36.79°E, 653 m, 21 September 2012; BS-HH-220-2011, Stn 220, 79.92°N, 6.54°E, 844 m, BT -0.70°C, 13 August 2011; BS-HH-218-2011, Stn 218, 79.86°N, 7.28°E, 757 m, BT 0.04°C, 13 August 2011; BS-JH-346-2011, Stn 346, 72.63°N, 18.73°E, 364 m, BT 4.07°C, 3 September 2011; BS-299-2011, Stn 299, 80.26°N, 44.75°E, 332.5 m, BT -0.54°C, 23 September 2011; BS-286-2011, Stn 286, 79.75°N, 40.06°E, 313.5 m, BT 0.03°C, 21 September 2011; BS-JM-56-2010, Stn 56, 73.95°N, 21.43°E, 382 m, BT 1.46°C, 4 September 2010; BS-256-2010, Stn 256, 81.14°N, 44.33°E, 217.5 m, BT 0.12°C, 19 September 2010.

Kara Sea: KS-376-2013, Stn 376, 79.47°N, 73.90°E, 405.5 m, BT -0.12°C, 23 October 2013; KS-257-2011, Stn 257, 82.04°N, 73.68°E, 622.5 m, BT -0.50° C, 14 September 2010; KS-255-2011, Stn 255, 81.28°N, 74.02°E, 397 m, BT 0.70°C, 14 September 2010; KS-252-2011, Stn 252, 80.82°N, 67.51°E, 541 m, BT -0.64°C, 13 September 2011; KS-251-2011, Stn 251, 80.76°N, 69.90°E, 568.5 m, BT -0.64°C, 13 September 2011; KS-249-2011, Stn 249, 80.74°N, 74.14°E, 161 m, BT -1.18°C, 13 September 2011; KS-242-2011, Stn 242, 79.25°N, 71.52°E, 489 m, BT -0.53°C, 11 September 2011; KS-240-2011, Stn 240, 78.28°N, 74.05°E, 385 m, BT -0.47°C, 11 September 2011; KS-239-2011, Stn 239, 78.26°N, 69.91°E, 463 m, BT -0.54°C, 11 September 2011; KS-188-2010, Stn 188, 81.54°N, 74.38°E, 440.5 m, BT -1.31°C, 9 September 2010; KS-34-2007, Stn 34, 72.99°N, 63.15°E, 86 m, BT -1.00°C, 25 September 2007.

*Muusoctopus johnsonianus* (Allcock, Strugnell, Ruggiero & Collins, 2006). East Greenland: GRL-PA-4-28-2016, Stn 28, 62.11°N, 40.20°W, 1262.5 m, BT 3.17°C, 27 July 2016.

*Muusoctopus sibiricus* (Løyning, 1930). Laptev Sea: LS-A-112, Stn A-112, 78.36°N, 136.21°E, 255 m, BT -1.05°C, 1 September 2014; LS-A-82, Stn A-82, 77.06°N, 132.07°E, 42 m, BT -1.70°C, 7 September 2014; LS-A-66, Stn A-66, 77.21°N, 137.07°E, 33 m, BT -1.05°C, 5 September 2014; LS-L-11, Stn L-11, 76.34°N, 129.92°E, 51 m, BT -1.74°C, 8 September 2014; LS-O-36, Stn O-36, 75.33°N, 120.38°E, 40 m, BT -1.53°C, 2 October 2014; LS-O-22, Stn O-22, 76.01°N, 122.75°E, 53 m, BT -1.67°C, 30 September 2014; LS-O-20, Stn O-20, 75.65°N, 121.55°E, 58 m, BT -1.66°C, 2 October 2014; LS-O-15, Stn O-15, 76.01°N, 120.48°E, 55 m, BT -1.56°C, 2 October 2014.

East Siberian Sea: ESS-A-68, Stn A-68, 77.46°N, 139.73°E, 34 m, BT 0.28° C, 31 August 2014.

**Table S1** Data on the studied immature individuals of *Muusoctopus aegir* Golikov, Gudmundsson & Sabirov, **sp. nov.** ML, mantle length; TL, total length; ICL, Iceland; BS, Barents Sea; KS, Kara Sea; n/a, not analysed

| **Individual/**  **character** | **BIOICE-**  **2369** | **BIOICE-**  **3124** | **BIOICE-**  **3242** | **BIOICE-**  **3659-1** | **BIOICE-**  **3659-2** | **BS-HH-**  **259-2012-2** | **BS-HH-**  **269-2012** | **KS-27-**  **2-2007** | **KS-25-**  **2007** | **KS-15-**  **2-2007** | **KS-27-**  **2-2007** | **KS-16-**  **2007** |
| --- | --- | --- | --- | --- | --- | --- | --- | --- | --- | --- | --- | --- |
| **Area** | ICL | ICL | ICL | ICL | ICL | BS | BS | KS | KS | KS | KS | KS |
| **Sex** | Male | Female | Female | Female | Juvenile | Female | Female | Male | Male | Male | Female | Female |
| **Maturity stage** | Early  immature  (I) | Late  immature  (II) | Late  immature  (II) | Early  immature  (I) | Juvenile  (0) | Late  immature  (II) | Late  immature  (II) | Late  immature  (II) | Late  immature  (II) | Late  immature  (II) | Late  immature  (II) | Early  immature  (I) |
| **ML, mm** | 12 | 19 | 16 | 8 | 4.5 | 31 | 31 | 29 | 27 | 19 | 21 | 17 |
| **TL, mm** | 65 | 103 | 69 | 37 | n/a | n/a | n/a | 140 | 120 | 98 | 104 | 85 |
| **Ventral ML, mm** | 10 | 16 | 15 | 6.5 | n/a | n/a | n/a | 27 | 24 | 18 | 19 | 16 |
| **Mantle width, mm** | 10 | 24 | 16 | 8.5 | n/a | n/a | n/a | 23 | 28 | 24 | 26 | 20 |
| **Head length, mm** | 6 | 9 | 8 | 3.5 | n/a | n/a | n/a | 12 | 10 | 8 | 10 | 7 |
| **Head width, mm** | 9 | 19 | 15 | 7.5 | n/a | n/a | n/a | 17 | 21 | 18 | 26 | 16 |
| **Eye diameter, mm** | 5.0 | 6.5 | 5.5 | 2.5 | n/a | n/a | n/a | 8.0 | 75 | 7.0 | 7.0 | 6.0 |
| **Lens diameter, mm** | 0.8 | 1.1 | 1.1 | 0.4 | n/a | n/a | n/a | 2.4 | 2.1 | 2.3 | 2.4 | 1.4 |
| **Funnel length, mm** | 5.0 | 7.5 | 3.0 | 2.7 | n/a | n/a | n/a | 11.0 | 10.0 | 9.0 | 10.0 | 8.0 |
| **Free funnel**  **length, mm** | 2.0 | 3.9 | 5.0 | 1.1 | n/a | n/a | n/a | 6.0 | 5.6 | 4.7 | 5.4 | 3.5 |
| **Web depth, mm**  **(min – max)** | 7–12 | n/a | 9–12 | 6–7 | n/a | n/a | n/a | 16–26 | 16–20 | 18–22 | 22–25 | 10–16 |
| **Web formula** | b>c=d>a>e | n/a | n/a | a=b=c=d>e | n/a | n/a | n/a | a>b=c>d>e | b=c>d>a>e | b>c=d>a>e | b>a>c=d>e | b=c>a>d>e |
| **Arm length, mm**  **(min – max)** | 39–47 | 69–75 | 44–51 | 22–25.5 | n/a | n/a | n/a | 92–99 | 72–83 | 61–71 | 67–71 | 56–61 |
| **Arm formula** | 1=2=3>4 | 2>1>3=4 | 2=3>1>4 | 1>2>3>4 | n/a | n/a | n/a | 1>2=3>4 | 1=2=3>4 | 1>2=3>4 | 2>1=3>4 | 1>2>3=4 |
| **Sucker count**  **(min – max)** | 76–80 | 94–100 | 78–90 | 70–74 | n/a | n/a | n/a | 84–88 | 82–98 | 92–94 | 84–92 | 84–90 |
| **Sucker diameter**  **(max), mm** | 1.1 | 2.0 | 1.4 | 0.7 | n/a | n/a | n/a | 2.3 | 2.5 | 1.9 | 1.8 | 1.3 |
| **Gill length, mm** | 5.0 | 5.5 | 5.0 | 1.8 | n/a | n/a | n/a | 9.0 | 11.0 | 8.0 | 8.0 | 7.5 |
| **Gill lamellae count,**  **outer/inner** | 8/7 | 8/7 | 9/8 | 8/7 | n/a | n/a | n/a | 8/7 | 8/7 | 8/7 | 8/7 | 8/7 |
| **Hectocotylized arm**  **length, mm** | 47^a^ | – | – | – | – | – | – | 74 | 59 | 52 | – | – |
| **Hectocotylized arm**  **sucker count** | 80^a^ | – | – | – | – | – | – | 50 | 50 | 50 | – | – |
| **Ligula length, mm** | –^a^ | – | – | – | – | – | – | 5.2 | 5.0 | 4.3 | – | – |
| **Ligula width, mm** | –^a^ | – | – | – | – | – | – | 2.2 | 2.2 | 1.3 | – | – |
| **Calamus length, mm** | –^a^ | – | – | – | – | – | – | 1.9 | 1.4 | 1.9 | – | – |
| **Fecundity** | – | 130 | n/a | n/a | – | n/a | n/a | – | – | – | 98 | 96 |

^a^hectocotylus not developed.

**Table S2** Measurements, indices and counts of *Muusoctopus aegir* Golikov, Gudmundsson & Sabirov, **sp. nov.** related to mantle length. Significant *p*-values are in **bold**. *n*, number of individuals; *r*^2^, determination coefficient; ML, mantle length

| **Measurement,**  **index or count** | **Equation** | **Measurement,**  **index or count** | **Equation** |
| --- | --- | --- | --- |
| **Mantle width, mm** | Mantle width = 0.94ML + 4.18  *n* = 30; *r*^2^ = 0.81; ***p* <0.0001** | **Sucker diameter, mm** | Sucker diameter = 0.08ML + 0.26  *n* = 30; *r*^2^ = 0.80; ***p* <0.0001** |
| **Mantle width, % ML** | Mantle width = –0.37ML + 120.01  *n* = 30; *r*^2^ = 0.05; *p* = 0.24 | **Sucker diameter, % ML** | Sucker diameter = –0.02ML + 9.31  *n* = 30; *r*^2^ = 0.01; *p* = 0.56 |
| **Head length, mm** | Head length = 0.35ML + 1.82  *n* = 30; *r*^2^ = 0.84; ***p* <0.0001** | **Gill length, mm** | Gill length = 0.27ML + 1.87  *n* = 30; *r*^2^ = 0.69; ***p* <0.0001** |
| **Head length, % ML** | Head length = –0.25ML + 49.51  *n* = 30; *r*^2^ = 0.19; ***p* = 0.0177** | **Gill length, % ML** | Gill length = –0.17ML + 39.35  *n* = 30; *r*^2^ = 0.06; *p* = 0.18 |
| **Head width, mm** | Head width = 0.57ML + 6.25  *n* = 30; *r*^2^ = 0.74; ***p* <0.0001** | **Hectocotylized arm length, mm** | Arm length = 2.45ML – 2.85  *n* = 14; *r*^2^ = 0.86; ***p* <0.0001** |
| **Head width, % ML** | Head width = –0.76ML + 102.55  *n* = 30; *r*^2^ = 0.74; ***p* = 0.0060** | **Hectocotylized arm length, % ML** | Arm length = –0.06ML + 238.36  *n* = 14; *r*^2^ = 0.0004; *p* = 0.95 |
| **Head width,**  **% mantle width** | Head width = –0.44ML + 86.99  *n* = 30; *r*^2^ = 0.14; ***p* = 0.0383** | **Opposite arm index, %**^a^ | Opposite arm index = –0.06ML + 74.36  *n* = 14; *r*^2^ = 0.0071; *p* = 0.79 |
| **Eye diameter, mm** | Eye diameter = 0.30ML + 0.70  *n* = 30; *r*^2^ = 0.88; ***p* <0.0001** | **Hectocotylized arm**  **sucker count** | Sucker count = 0.14ML + 47.42  *n* = 12; *r*^2^ = 0.11; *p* = 0.30 |
| **Eye diameter, % ML** | Eye diameter = –0.12ML + 36.07  *n* = 30; *r*^2^ = 0.09; *p* = 0.12 | **Ligula length, mm** | Ligula length = 0.26ML – 1.03  *n* = 14; *r*^2^ = 0.86; ***p* <0.0001** |
| **Lens diameter, mm** | Lens diameter = 0.11ML – 0.44  *n* = 30; *r*^2^ = 0.83; ***p* <0.0001** | **Ligula length,**  **% hectocotylized arm length** | Ligula length = 0.05ML + 8.09  *n* = 14; *r*^2^ = 0.07; *p* = 0.37 |
| **Lens diameter,**  **% eye diameter** | Lens diameter = 0.34ML + 18.13  *n* = 30; *r*^2^ = 0.25; ***p* = 0.0049** | **Ligula width, mm** | Ligula width = 0.07ML + 1.53  *n* = 13; *r*^2^ = 0.14; *p* = 0.19 |
| **Funnel length, mm** | Funnel length = 0.29ML + 3.28  *n* = 30; *r*^2^ = 0.71; ***p* <0.0001** | **Ligula width,**  **% ligula length** | Ligula width = 0.29ML + 42.19  *n* = 13; *r*^2^ = 0.07; *p* = 0.35 |
| **Funnel length, % ML** | Funnel length = –0.30ML + 50.50  *n* = 30; *r*^2^ = 0.16; ***p* = 0.0285** | **Calamus length, mm** | Calamus length = 0.05ML + 0.98  *n* = 13; *r*^2^ = 0.16; *p* = 0.17 |
| **Free funnel length, mm** | Free funnel length = 0.18ML + 1.06  *n* = 30; *r*^2^ = 0.73; ***p* <0.0001** | **Calamus length,**  **% ligula length** | Calamus length = 0.23ML + 29.05  *n* = 13; *r*^2^ = 0.14; *p* = 0.18 |
| **Free funnel length,**  **% funnel length** | Free funnel length = 0.30ML + 43.77  *n* = 30; *r*^2^ = 0.41; ***p* = 0.0001** | **Spermatopore number** | Spermatophore number = 0.36ML – 1.01  *n* = 8; *r*^2^ = 0.23; *p* = 0.23 |
| **Arm length, mm** | Arm length = 2.84ML + 8.79  *n* = 30; *r*^2^ = 0.89; ***p* <0.0001** | **Spermatophore length, mm** | Spermatophore length = 0.35ML + 25.32  *n* = 8; *r*^2^ = 0.51; ***p* = 0.0440** |
| **Arm length, % ML** | Arm length = –1.07ML + 349.09  *n* = 33; *r*^2^ = 0.12; *p* = 0.0529 | **Spermatophore length, % ML** | Spermatophore length = –1.80ML + 173.32  *n* = 8; *r*^2^ = 0.78; ***p* = 0.0031** |
| **Sucker count** | Sucker count = 0.56ML + 77.83  *n* = 31; *r*^2^ = 0.45; ***p* <0.0001** | **Fecundity** | Fecundity = –0.15ML + 103.85  *n* = 16; *r*^2^ = 0.0028; *p* = 0.85 |

^a^length of hectocotylized arm (third left) to length of third right arm, %.

**Table S3** Data on the studied immature individuals of *Muusoctopus johnsonianus* (Allcock, Strugnell, Ruggiero & Collins, 2006). All individuals are from Iceland. ML, mantle length; TL, total length; n/a, not analysed

| **Individual/**  **character** | **BIOICE-**  **2427** | **BIOICE-**  **3520-2** | **BIOICE-**  **3521** | **BIOICE-**  **2926** |
| --- | --- | --- | --- | --- |
| **Sex** | Male | Female | Female | Female |
| **Maturity stage** | Early  immature  (I) | Late  immature  (II) | Late  immature  (II) | Early  immature  (I) |
| **ML, mm** | 8.5 | 41 | 36 | 13 |
| **TL, mm** | 41.5 | 226 | 190 | 54 |
| **Ventral ML, mm** | 7 | 35 | 34 | 12 |
| **Mantle width, mm** | 9 | 42 | 37 | 16 |
| **Head length, mm** | 4 | 22 | 20 | 6 |
| **Head width, mm** | 8.5 | 45 | 42 | 12 |
| **Eye diameter, mm** | 3.5 | 21.0 | 19.0 | 4.3 |
| **Lens diameter, mm** | 1.3 | 5.5 | 7.0 | 0.7 |
| **Funnel length, mm** | 3.6 | 20.0 | 16.0 | 6.0 |
| **Free funnel**  **length, mm** | 1.8 | 11.0 | 8.0 | 3.0 |
| **Web depth, mm**  **(min – max)** | 4–7 | 27–35 | 26–39 | n/a |
| **Web formula** | a=b>c>d>e | b=c>a>d>e | a>b>c=d>e | n/a |
| **Arm length, mm**  **(min – max)** | 26–29 | 132–163 | 111–134 | 33–35 |
| **Arm formula** | 1=2>3>4 | 1>2>3=4 | 2>1>4>3 | 1>2>3=4 |
| **Sucker count**  **(min – max)** | 82–88 | 112–130 | 112–120 | 68–72 |
| **Sucker diameter**  **(max), mm** | 0.6 | 3.0 | 3.0 | 1.1 |
| **Gill length, mm** | 2.5 | 13.0 | 12.0 | 4.5 |
| **Gill lamellae count,**  **outer/inner** | 9/8 | 11/10 | 11/10 | 10/9 |
| **Hectocotylized arm**  **length, mm** | 21^a^ | – | – | – |
| **Hectocotylized arm**  **sucker count** | 68^a^ | – | – | – |
| **Ligula length, mm** | –^a^ | – | – | – |
| **Ligula width, mm** | –^a^ | – | – | – |
| **Calamus length, mm** | –^a^ | – | – | – |
| **Fecundity** | – | 250 | 300 | n/a |

^a^hectocotylus not developed.

**Table S4** Data on the studied immature individuals of *Muusoctopus sibiricus* (Løyning, 1930). Both individuals are from East Siberian Sea. ML, mantle length; TL, total length; n/a, not analysed

| **Individual/**  **character** | **ESS-A-51** | **ESS-A-27^a^** |
| --- | --- | --- |
| **Sex** | Female | Female |
| **Maturity stage** | Early  immature  (I) | Early  immature  (I) |
| **ML, mm** | 14 | 10 |
| **TL, mm** | 48 | n/a |
| **Ventral ML, mm** | 13 | n/a |
| **Mantle width, mm** | 13 | n/a |
| **Head length, mm** | 4 | n/a |
| **Head width, mm** | 11 | n/a |
| **Eye diameter, mm** | 4.0 | n/a |
| **Lens diameter, mm** | 1.1 | n/a |
| **Funnel length, mm** | 6.5 | n/a |
| **Free funnel**  **length, mm** | 3.2 | n/a |
| **Web depth, mm**  **(min – max)** | 8–11 | n/a |
| **Web formula** | c>b>d>a=e | n/a |
| **Arm length, mm**  **(min – max)** | 29–30 | n/a |
| **Arm formula** | 1=2>3=4 | n/a |
| **Sucker count**  **(min – max)** | 70–76 | n/a |
| **Normal sucker diameter**  **(max), mm** | 1.0 | n/a |
| **Enlarged suckers,**  **diameter (min – max), mm**  **and location** | Enlarged  suckers  absent | n/a |
| **Gill length, mm** | 8.0 | n/a |
| **Gill lamellae count,**  **outer/inner** | 10/10 | n/a |
| **Fecundity** | 136 | – |

^a^found in stomach of *Gymnocanthus tricuspis* (Reinhardt, 1830) (Osteichthyes), heavily digested.

**Fig. S1** Bootstrapped neighbor-joining Cytochrome *c* oxidase subunit I (*COI*) molecular taxonomic cladogram including all species of *Muusoctopus*, *Benthoctopus* and *Vulcanoctopus* with available *COI* sequence in open access in GenBank (<https://www.ncbi.nlm.nih.gov/genbank/>) and BOLD (<https://www.boldsystems.org/>) databases on 18 August 2023. Values on the phylogenetic tree represent bootstrap percentages. Node values only show probabilities above 50. Based on the Tamura-Nei substitution model (TN93) with gamma distribution. Rooted using *Octopus vulgaris* Cuvier, 1797, MW560654, as the outgroup. Our sequence of *M. sibiricus* (Løyning, 1930), individual LS-L-3 from the Laptev Sea, is in **bold red**. GenBank accession numbers are shown for all sequences


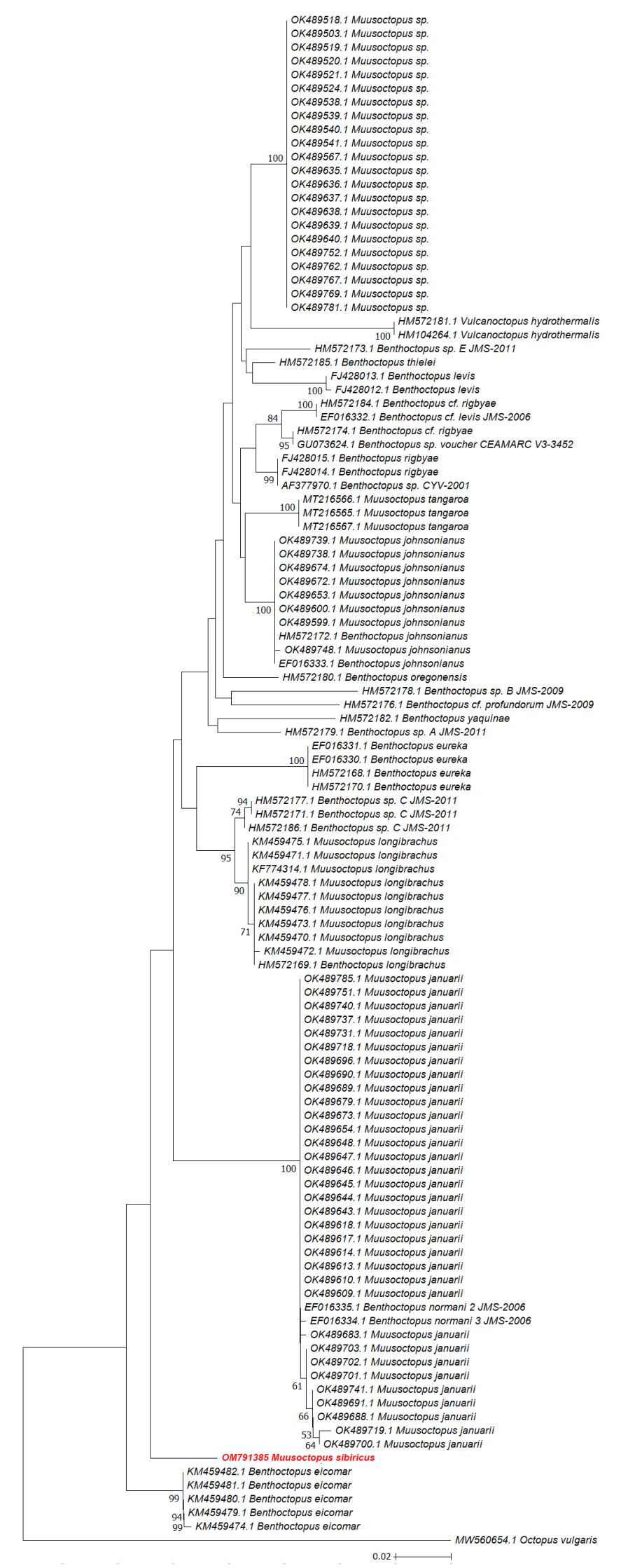


**Fig. S2** Sampling locations and *Muusoctopus* records from BIOICE program, *Muusoctopus* records from Taite *et al.* (2023) and ‘historical’ records of ‘*Benthoctopus piscatorum*’ in the Faroe–Shetland Channel


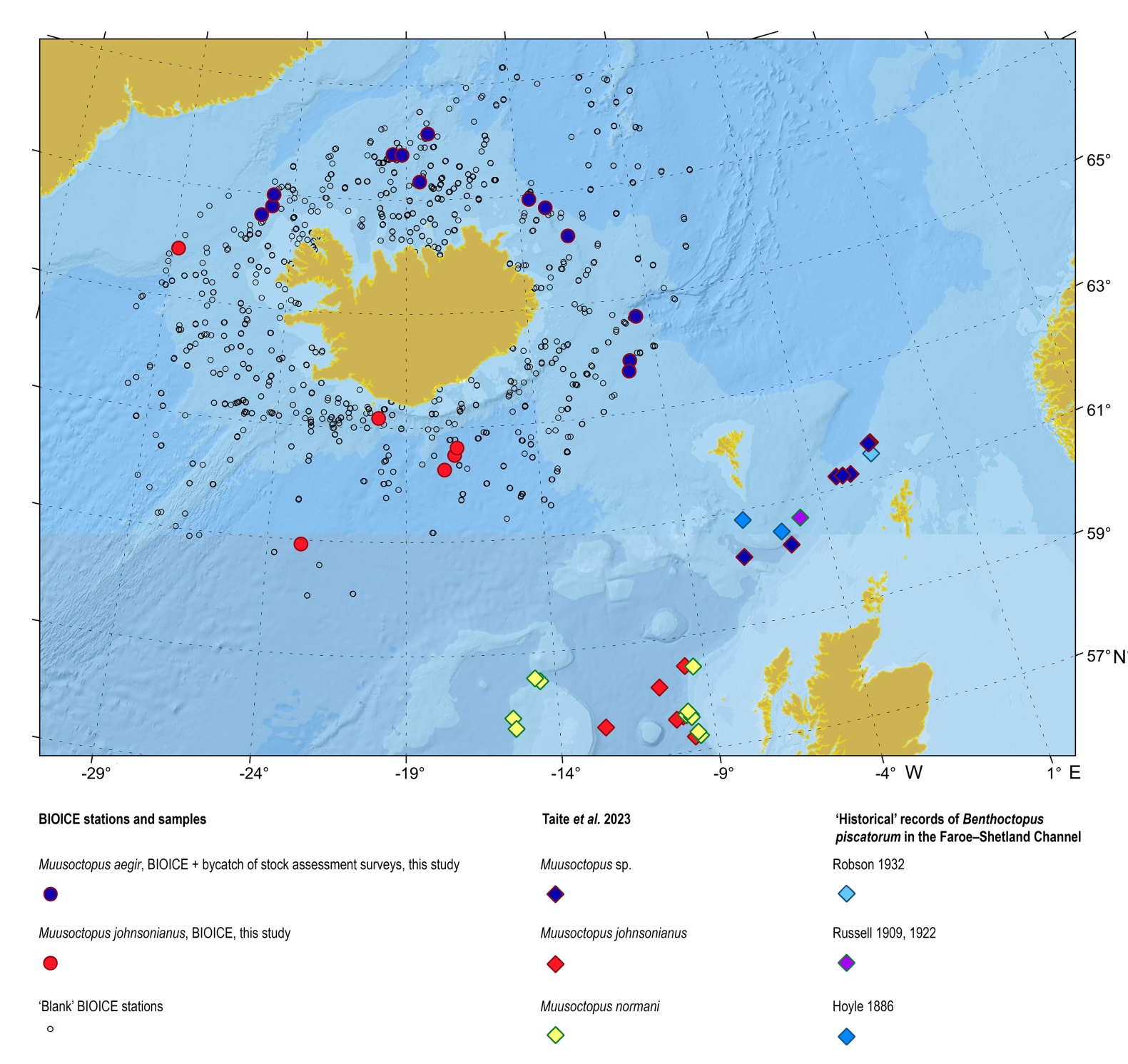

Supplement: Supplementary file 1 — Additional file 1. [file 40851_2023_220_MOESM1_ESM.docx]
